# The Effect of Natural Pozzolanic Coated Waste Tire Aggregates on the Mechanical, Transport and Durability Properties of Fiber-Reinforced and One-Part Hybrid Geopolymer Composites

**DOI:** 10.3390/polym18162014

**Published:** 2026-08-19

**Authors:** Wiam Abdelmagid Taher Elabade, Oğuzhan Yavuz Bayraktar, Halil Oğuzhan Kara, İhsan Kasım Karataş, Mehmet Uğur Yılmazoğlu, Adem Ahıskalı, Mohamed A. Salem Elmekahal, Gökhan Kaplan

**Affiliations:** 1 Department of Civil Engineering, Institute of Graduate Education, Kastamonu University, Kastamonu 37150, Türkiye; 221094004@ogr.kastamonu.edu.tr (W.A.T.E.); 181062818@ogr.kastamonu.edu.tr (M.A.S.E.); 2 Department of Civil Engineering, Kastamonu University, Kastamonu 37150, Türkiye; obayraktar@kastamonu.edu.tr (O.Y.B.); myilmazoglu@kastamonu.edu.tr (M.U.Y.); ahiskali@kastamonu.edu.tr (A.A.); 3 Department of Construction Technology, Kastamonu Vocational School, Kastamonu University, Kastamonu 37150, Türkiye; ikkaratas@kastamonu.edu.tr; 4 Department of Civil Engineering, Atatürk University, Erzurum 25240, Türkiye; gkaplan@atauni.edu.tr

**Keywords:** one-part geopolymer, hybrid geopolymer composite, waste tire aggregate, pumice coating, polypropylene fiber, durability

## Abstract

This study examined the effects of coating waste tire aggregates (WTAs) with pumice, perlite, or diatomite, combined with polypropylene (PP) fiber addition, on the fresh, mechanical, transport, and durability properties of one-part hybrid geopolymer composites. Sixteen mixtures were produced using a Taguchi L16 design with a binder system of fly ash, CEM II/B-S cement, and sodium metasilicate powder. Coating type, WTA ratio, and PP fiber content were the key performance factors. Pumice coating performed best overall by improving the interfacial transition zone: 28-day compressive strength reached 15.5 MPa and flexural strength 1.60 MPa, while porosity and capillary water absorption decreased significantly. Among the studied WTA levels, 10% WTA yielded the most positive direct responses in compressive strength, flexural strength, toughness, and capillary water absorption, whereas higher contents weakened matrix continuity. The effect of PP fiber was response-dependent: 0.5% fiber maximized compressive strength and durability-related responses, while 2% fiber gave the greatest flexural strength and toughness; no single dosage was universally optimal. The pumice-coated series was also the most stable under high temperature, freeze–thaw, MgSO_4_, and H_2_SO_4_ exposure. Overall, waste tire aggregates can be technically incorporated into one-part hybrid geopolymer composites; a dedicated life-cycle assessment is nevertheless required to quantify the net environmental benefit.

## 1. Introduction

The decarbonization of construction materials has become a key focus due to climate change, resource depletion, and the rising demand for circular production systems. Among these materials, concrete remains essential for its versatility, affordability, and widespread availability; however, its environmental impact is primarily driven by ordinary Portland cement (OPC), which entails high-temperature clinker production, heavy fuel consumption, and significant CO_2_ emissions [1,2,3]. As a result, the cement industry is recognized as a major contributor in life-cycle assessments of buildings and infrastructure [4,5]. In this context, the development of low-carbon binders that can reduce greenhouse gas emissions and raw material use has attracted ongoing scientific research [6,7].

Alkali-activated materials and geopolymers are emerging as promising options during this transition, mainly because they allow the conversion of aluminosilicate-rich wastes and natural pozzolans into high-performance binders [8,9,10]. Their reaction mechanisms generally involve dissolution, reorganization, and polycondensation, resulting in three-dimensional binding networks such as N-A-S-H, C-(A)-S-H, or hybrid gels, depending on the precursor composition and the presence of calcium [11,12]. Compared to OPC systems, geopolymer-based materials often have lower embodied carbon, better chemical resistance, good thermal stability, and competitive mechanical properties [13]. Nonetheless, these advantages are sometimes overshadowed by practical challenges in large-scale use, particularly with traditional two-part geopolymer systems that rely on corrosive liquid activators. This reliance causes issues with storage, transportation, worker safety, and on-site mixing [14].

To address these limitations, one-part or “just-add-water” geopolymer systems have gained increasing attention. In such systems, solid precursors and solid activators are dry-blended in advance, and geopolymerization is triggered only upon the addition of water, making the process more compatible with traditional cement-based construction practices [15,16]. This operational simplification is especially relevant from a cleaner production standpoint because it enhances constructability while maintaining the sustainability benefits of alkali activation. Hybrid one-part formulations, in particular, show strong potential, as the coexistence of cement hydration and alkali-activation reactions can produce a denser matrix by simultaneously developing geopolymeric and calcium-rich reaction products [17,18]. In this study, this hybrid framework was established using fly ash, CEM II/B-S cement, and powdered sodium metasilicate. At the same time, the selected coating minerals also offered silica-rich surfaces, with measured SiO_2_ contents of 67.8% for pumice, 72.7% for perlite, and 82.5% for diatomite.

In addition to innovations in binders, the reuse of problematic waste streams has become a crucial aspect of sustainable material design. End-of-life vehicle tires are among the most persistent types of solid waste due to their non-biodegradability, large volume, fire hazards, and long-term environmental impacts when improperly disposed of [19,20,21]. Their integration into cementitious composites is a common approach to waste valorization and reducing reliance on natural aggregates [22,23,24]. In both OPC-based and alkali-activated systems, rubber aggregates have been shown to decrease unit weight, enhance deformability, increase damping capacity, and improve impact resistance and energy absorption [25,26]. For instance, replacing all natural sand with crumb rubber in geopolymer mortar can decrease density and thermal conductivity by approximately 42% and 79%, respectively [22]. These qualities make rubber-modified binders appealing for applications such as lightweight panels, insulation units, vibration-damping layers, and non-structural components.

However, the main drawback of rubberized cementitious materials is the ongoing loss of mechanical strength and matrix stability [24,25,26]. This issue mainly stems from the weak interfacial transition zone (ITZ) between hydrophobic rubber particles and the surrounding mineral matrix. Waste tire rubber typically has a smooth, chemically inert, and water-repellent surface, which hinders wetting, lowers interfacial adhesion, and promotes void formation and stress concentration [27,28,29]. Previous studies have shown that increasing rubber size or content enlarges ITZ microcracks and weakens stress transfer between the aggregate and the matrix [30,31]. Park et al. [32] reported that replacing sand with crumb rubber in fly ash-based geopolymer concrete reduced compressive strength by up to 35%. Additionally, other research has found that rubberized geopolymers tend to have poor compaction unless the interface is deliberately modified [26,33]. Therefore, the environmental benefits of tire recycling in geopolymer production are often negated by structural issues arising from interfacial incompatibility.

Various pretreatment techniques have been proposed to improve rubber–matrix adhesion, including alkaline washing, silane treatment, latex modification, oxidizing treatments, and mineral or polymer coatings [27,34,35]. Although these methods can partially restore compressive and flexural properties, they often require additional chemicals, multiple processing steps, or higher energy consumption, thereby affecting eco-efficiency and scalability [28,36,37]. From a practical production standpoint, a preferable solution would be a simple, low-toxicity, mineral-based interface engineering technique that enhances compatibility without sacrificing the environmental advantages of waste reuse.

Natural pozzolans such as pumice, perlite, and diatomite appear particularly promising. These abundant, lightweight, silica-rich materials possess rough, porous structures that can enhance mechanical interlocking and encourage secondary reaction products in alkaline conditions [38,39,40]. Perlite has been shown to improve the transport properties of cementitious and geopolymer systems when used at optimal levels. Simultaneously, diatomite and diatomaceous earth are regarded as effective silica sources for alkali-activated matrices [41,42]. Pumice, similarly, has long been recognized as a reactive volcanic material that can contribute to matrix densification and greater durability [18]. Although these materials are studied as supplementary cementitious or lightweight components, their use as surface-coating agents for waste tire aggregate in one-part hybrid geopolymer composites remains very limited. This is significant because a mineral shell around the rubber could transform the ITZ from a weak discontinuity into a chemically and physically active transition zone.

The significance of fiber reinforcement should also be recognized in such a multi-phase system. Geopolymer matrices usually have high compressive strength but limited tensile ductility, tending to behave in a brittle way after cracking [43,44,45]. To improve crack bridging, toughness, and load transfer after peak stress is reached in alkali-activated composites, polypropylene (PP) fibers are frequently employed [43,44,45]. Their success is often attributed to microcrack control, stress redistribution, and energy dissipation via fiber pull-out, as though excessive fiber can impair workability [46,47]. In rubberized composites, this role is especially critical because rubber particles increase deformability but cannot effectively bridge cracks on their own. Thus, combining PP fibers with mineral-coated WTA offers a multiscale toughening strategy, in which the coating enhances the interfacial transition zone (ITZ) quality, and the fibers bolster post-cracking performance.

This study addresses a notable gap in research on waste-valorizing, one-part hybrid geopolymer composites. While prior work has explored individual aspects such as one-part geopolymer binders, waste-tire aggregates, fiber reinforcement, and rubber surface treatments, the use of natural pozzolanic coatings as an interface-engineering method for waste-tire aggregates in such systems has been largely overlooked. Specifically, the individual (main) effects of pumice-, perlite-, and diatomite-based surface coatings and of polypropylene fiber reinforcement on rubber–matrix adhesion and overall composite performance have not been thoroughly investigated. Therefore, this research aims to develop a simple, just-add-water hybrid geopolymer composite in which waste tire aggregates are functionally modified with natural pozzolans and the brittle matrix is reinforced with PP fibers. Its originality stems from combining reduced-clinker hybrid binder design, waste tire valorization, mineral-based surface functionalization, and fiber-reinforced toughening into a single material solution, offering a resource-efficient and technically viable approach to overcoming interfacial challenges in rubberized geopolymer composites.

The necessity of this research is threefold. First, although rubberized geopolymer/cement composites have been widely studied, the strength loss caused by the hydrophobic rubber–matrix interface remains the principal barrier to their practical use, and existing pretreatments (chemical washing, silane coupling, polymer modification) are either costly, toxic, or difficult to scale. Second, one-part (‘just-add-water’) hybrid geopolymer systems are far more compatible with conventional construction practice than two-part systems, yet no study has systematically addressed how a mineral-based surface coating on waste tire aggregates performs within such one-part hybrid matrices. Third, the combined use of three natural pozzolans with distinct morphologies (pumice, perlite, diatomite) as functional aggregate coatings—rather than as binder replacements—introduces a novel interface-engineering route whose systematic factor screening and response-specific assessment using a Taguchi L16 design has not been reported to date. The novelty of this work therefore lies not in the individual constituents, but in demonstrating and optimizing a scalable, mineral-based coating strategy that converts the rubber surface from the weakest link into a reactive interface within a one-part hybrid geopolymer composite.

## 2. Materials and Methods

### 2.1. Raw Materials

**Figure 1:**
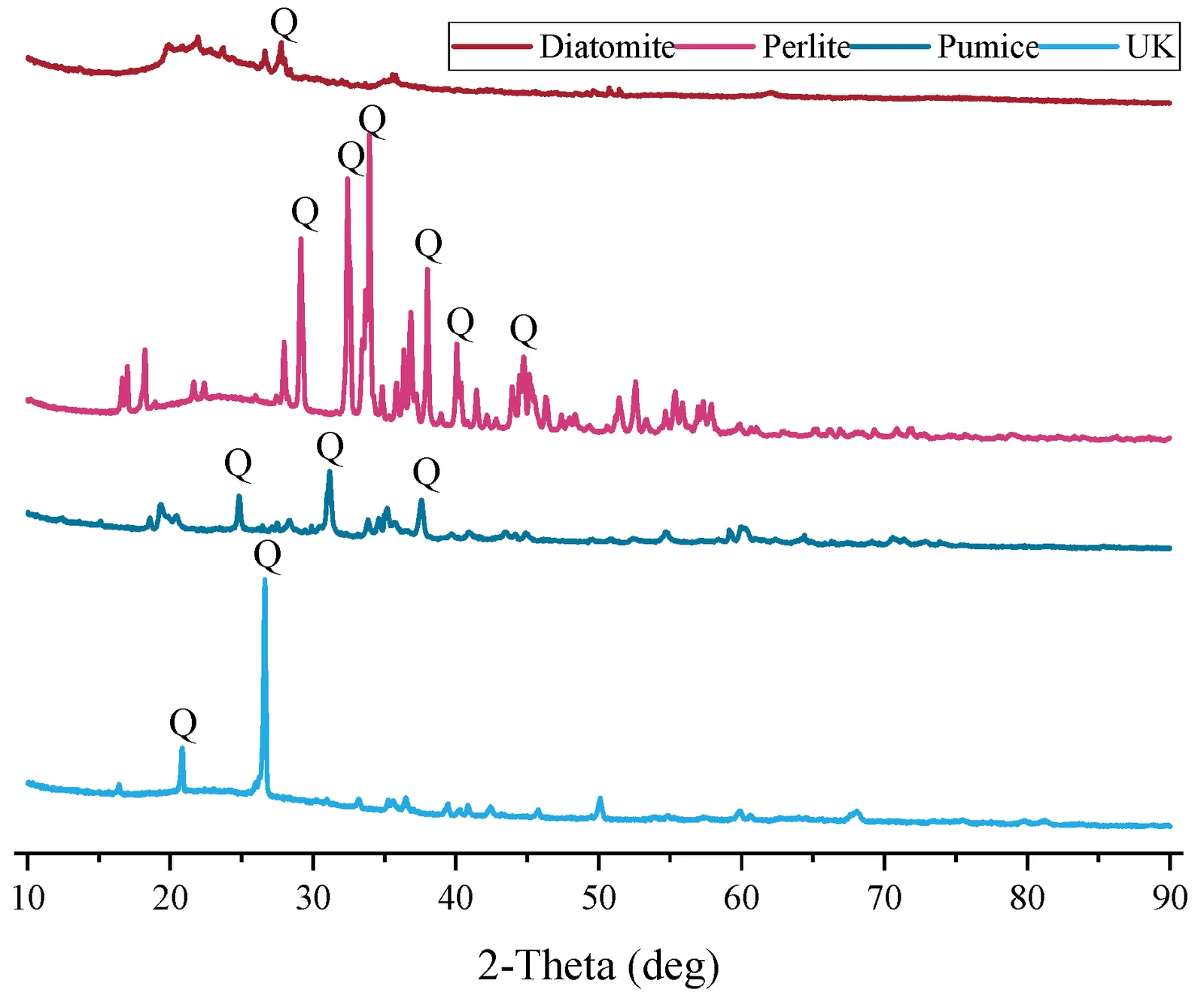
Characterization of binding and coating materials. (Q: Quartz).

**Figure 2:**
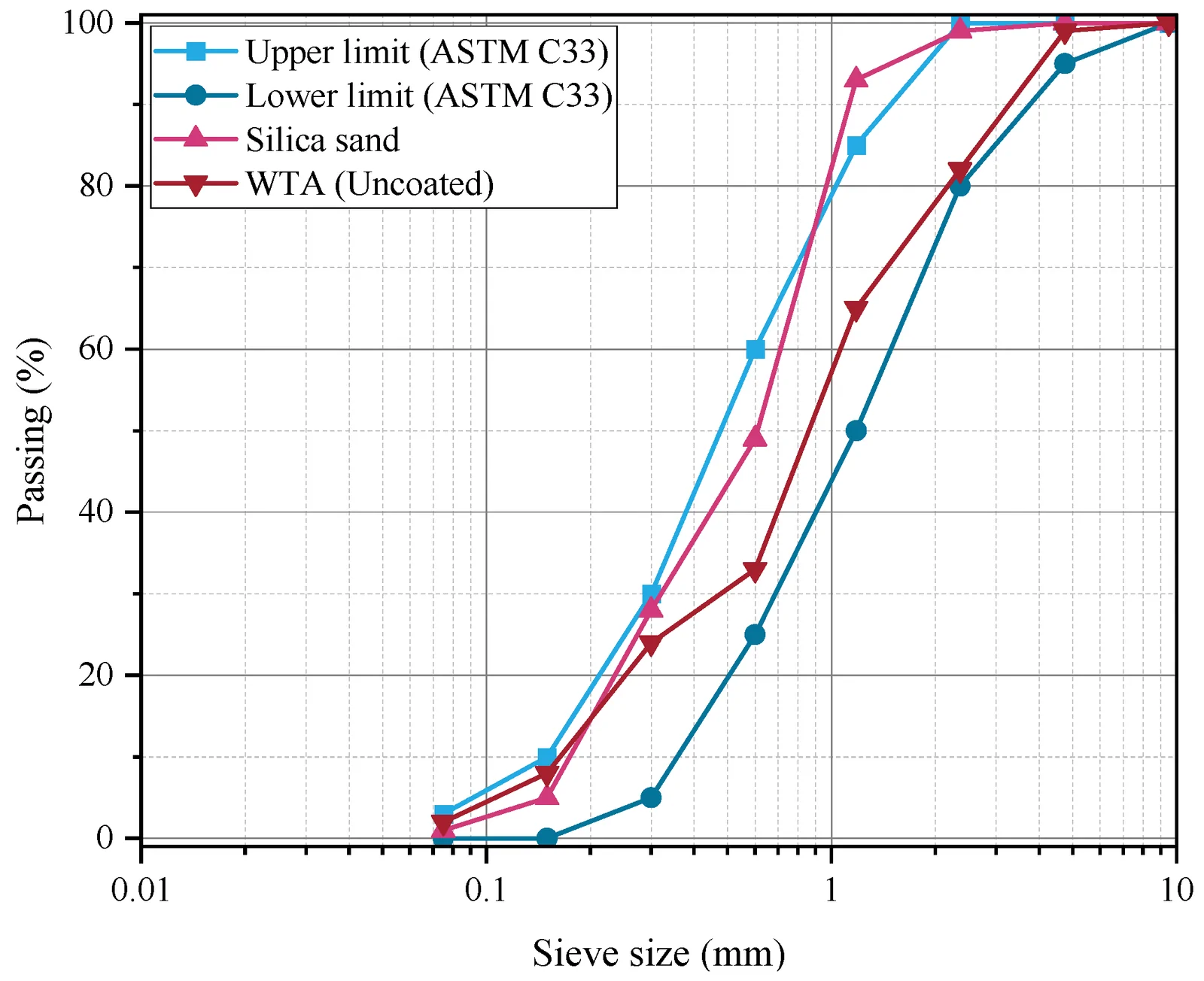
Particle size distribution of silica sand and WTA.

Fly ash (FA) and CEM II/B-S 42.5 N Portland cement served as the primary binding components in the production of hybrid geopolymer composites. The selected fly ash was a low-calcium aluminosilicate. Chemical analysis showed it mainly contains high levels of amorphous silica (SiO_2_) and aluminum oxide (Al_2_O_3_). Due to these properties, it proved to be an appropriate precursor for the formation of N-A-S-H-type gels during alkali activation. To create a hybrid binder system in the mixtures, Portland slag cement of type CEM II/B-S 42.5 N, compliant with EN 197-1 [48], was used. This type of cement, due to its high content of oven-granulated slag in a certain proportion, combined with the clinker composition, contributed to the formation of C-S-H structures through hydration reactions and also supported the development of secondary binder phases (C-A-S-H) in an alkaline environment. The CaO content in the cement’s chemical structure has been identified as a critical factor in early-age strength development and microstructural densification. The chemical properties of UK and cement are given in Table 1. The mineralogical properties of the UK are shown in Figure 1, and the particle-size distribution in Figure 2.

Natural pozzolans, such as pumice, perlite, and diatomite, were used to enhance the surface properties of aggregates derived from waste tires and to promote pozzolanic activity within the hybrid aggregate system. The pumice employed in this research is a volcanic-origin, silica-rich material with a porous, lightweight structure. Its high amorphous silica content makes it reactive in alkaline settings, while its light weight helps lower the composite’s unit volume weight. Perlite is valued for its low thermal conductivity and high porosity. In this study, perlite was selected to boost the system’s thermal performance and improve the interface compatibility between rubber aggregates and the binder matrix. Diatomite is a biogenic siliceous material formed by the fossilization of the frustules of diatoms (unicellular algae), characterized by a high amorphous silica content, a very fine pore network, and a very high specific surface area. Its large specific surface area accelerates gelation reactions and increases matrix density during alkali activation. The chemical composition of these pozzolans is provided in Table 1, and their mineralogical and physical properties are shown in Figure 1.

X-ray diffraction (XRD) analysis of the binding and coating materials was performed using a Bruker D8 Advance diffractometer with Cu Kα radiation (λ = 1.5406 Å), operated at 40 kV and 40 mA, over a 2θ range of 5–70° with a step size of 0.02° (standard instrument settings). Phase identification was carried out using the ICDD PDF database. The particle size distributions of silica sand, WTA and the ground natural pozzolans were determined by sieve analysis; the ground pozzolans were verified to pass at least 90% through a 90 µm sieve.

Natural silica sand with a particle size of 0–2 mm was used as a fine aggregate to enhance workability and maintain matrix continuity in the mixtures. Selected for its high silica content and durable particles, this aggregate has a specific gravity of 2.70 and absorbs 1.12% water over 24 h. In the study, waste tire aggregates (WTA) with a sieve size of 0–4 mm, produced by mechanically shredding end-of-life vehicle tires, were used as the primary component of the lightweight aggregate system. The low specific gravity (1.09) and elastic nature of WTA were examined to lower the unit volume weight of geopolymer composites and enhance their damping capacity. However, due to the hydrophobic surfaces and smooth textures of the rubber particles, a surface treatment with pozzolanic powders was applied to improve the interfacial bond with the matrix. The WTA’s water absorption rate was very low at 1.96%, helping maintain the water/binder ratio of the fresh mixture. The particle size distributions of silica sand and WTA, as determined by sieve analysis, are presented in Figure 2.

Sodium metasilicate (Na_2_SiO_3_) powder served as an alkaline activator to initiate geopolymerization. Using the powdered form facilitated the creation of user-friendly “one-part” geopolymer systems and reduced mixing challenges associated with liquid activators. The sodium metasilicate comprised high-purity silica (SiO_2_) and sodium oxide (Na_2_O), with its low moisture content aiding uniform distribution during dry mixing. Its chemical properties are summarized in Table 2.

To improve the workability of fresh geopolymer mortars while maintaining matrix density by keeping the water/binder ratio constant, a new polycarboxylate ether (PCE)- based superplasticizer was employed. PCE’s high-molecular-weight polymer structure markedly increased the system’s fluidity by activating steric hindrance and electrostatic repulsion at the binder particles. This additive, selected to counteract the rise in viscosity caused by waste rubber and pozzolanic dust, ensured proper spread values even at low dosages and facilitated mold placement during casting. PCE has a specific gravity of 1.06 ± 0.02 and a solid content of 38%.

High-performance 12 mm PP fibers were incorporated to enhance the ductility of the hybrid geopolymer matrix and reduce microcracks caused by drying shrinkage. PP fibers were chosen mainly for their high chemical resistance in alkaline conditions, low specific gravity, and ability to be evenly dispersed within the matrix. Their high tensile strength and elastic modulus significantly improved the composite’s crack bridging under mechanical stress. The technical specifications of PPF’s given in Table 3. Although PP fibers are inherently hydrophobic and non-polar, their high tensile strength and elastic modulus provide effective crack bridging under mechanical stress through mechanical interlocking with the geopolymer matrix.

### 2.2. Surface Coating and Improvement Process

Natural pozzolans, sourced directly from quarries in their raw form, were initially classified by particle size into the 0–4 mm range. To enhance their pozzolanic reactivity and improve their ability to penetrate the surfaces of rubber particles, they were ground in a ball mill for 90 min to achieve micronization. The resulting particle size distribution and morphological features of these powders significantly influenced their bonding capacity within the composite system. It was found that 90% of the ground pozzolans could pass through a 90 μm sieve.

**Figure 3:**
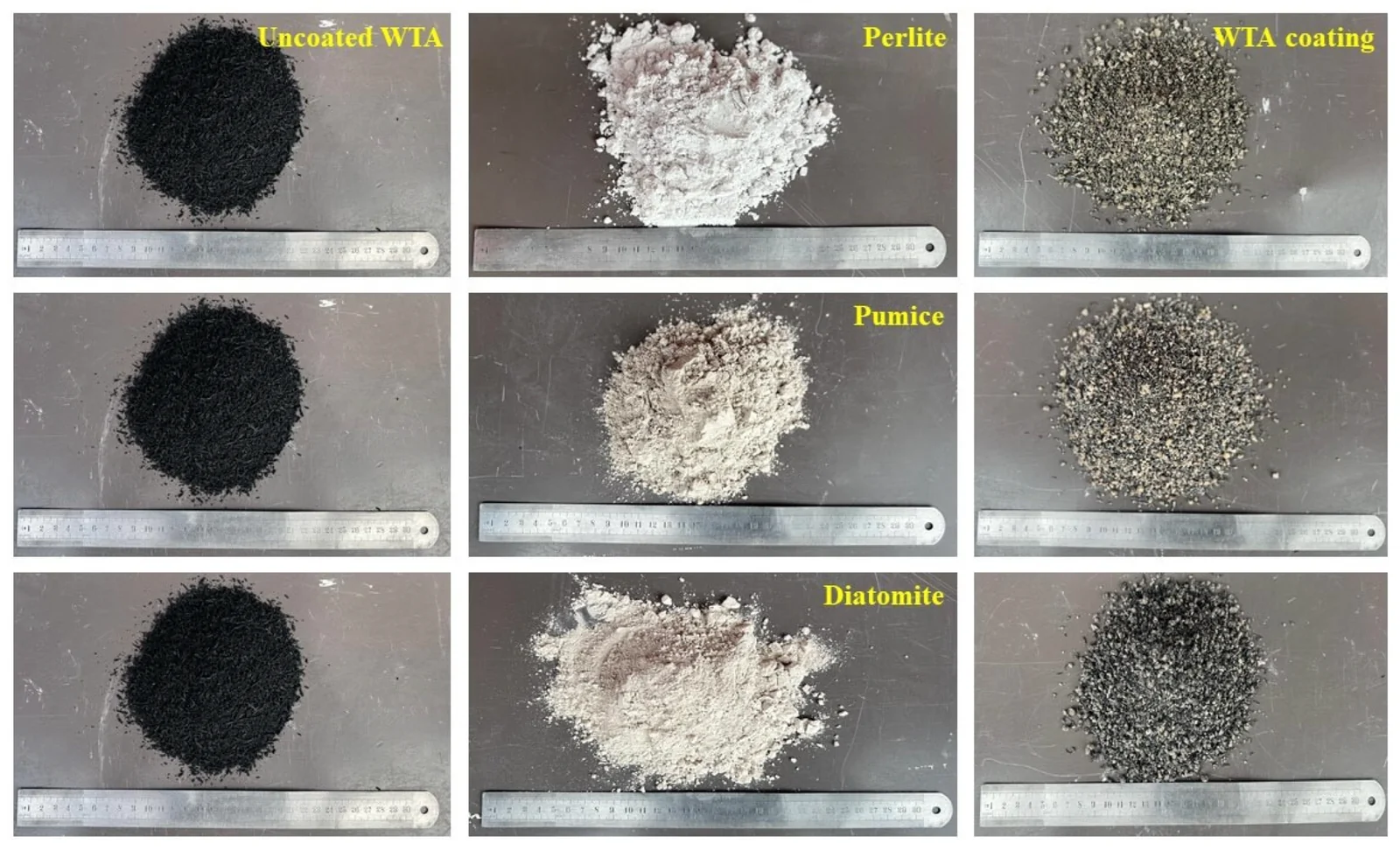
WTA samples coated and uncoated with different natural pozzolans.

**Figure 4:**
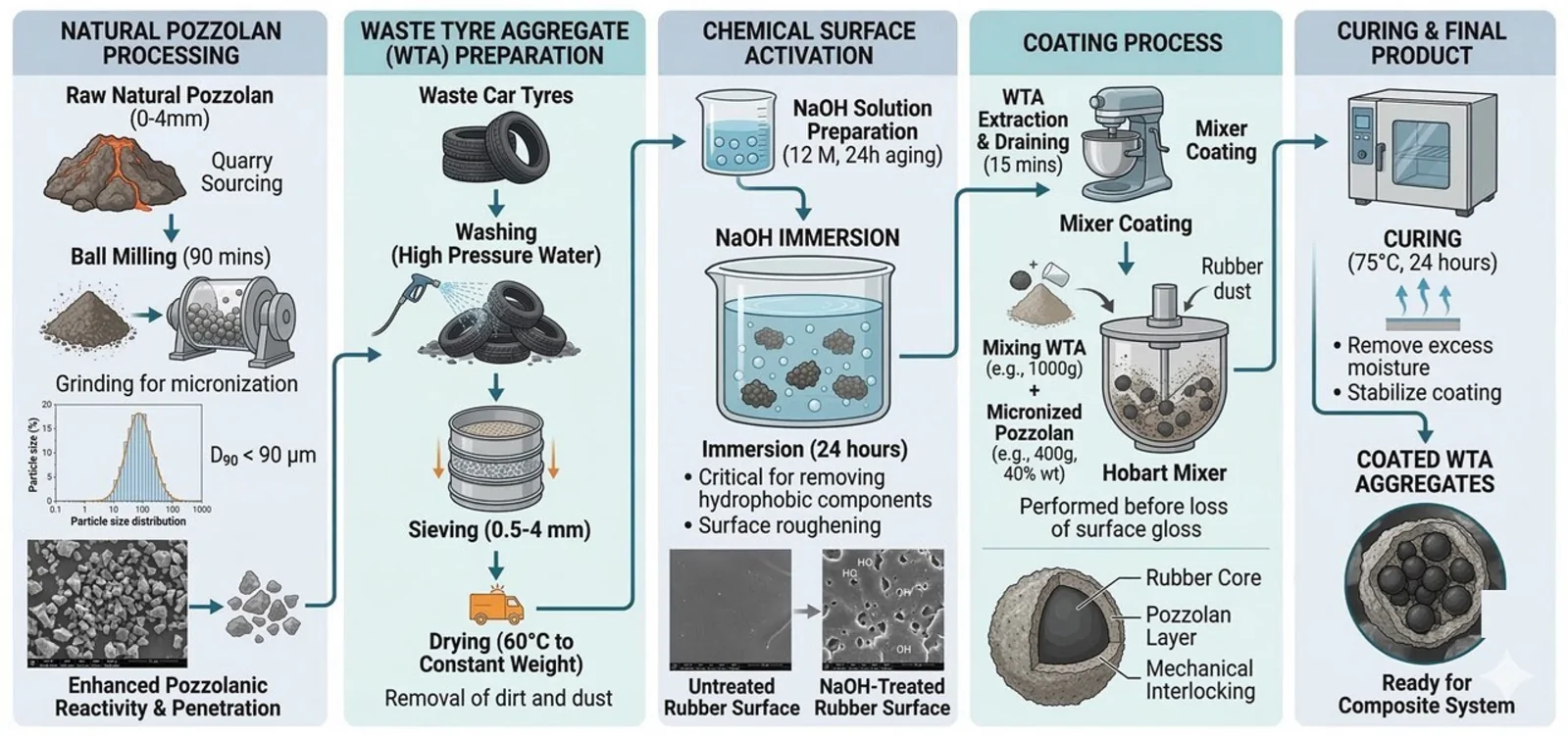
The process of coating WTA with natural pozzolans.

Subsequently, waste tire aggregates obtained from waste car tires were first washed with water (under pressure) to remove dirt and dust, then dried at 60 °C until they reached a constant weight. The WTA particles were classified using a 0.5–4 mm sieve and then immersed in a NaOH solution. The NaOH solution was prepared to a concentration of 12 M 24 h before immersion. The solution volume was adjusted to fully cover all rubber aggregates and provide a sufficient reaction environment. The rubber aggregates were left in the NaOH solution for 24 h. This period is critical for removing hydrophobic components and roughening the surface. The excess NaOH solution on the WTA surface was allowed to drain off for approximately 15 min. This duration was determined based on preliminary experiments. The coating process with natural pozzolans was carried out before the gloss on the WTA surface disappeared. The surface coating process was performed by mixing WTA and natural pozzolan (e.g., 1000 g of WTA and 400 g of natural pozzolan), equivalent to 40% of the WTA weight, using a Hobart mixer. After the coating process, it was cured at 75 °C for 24 h in the WTA to remove excess moisture and stabilize the coating. Figure 3 shows WTA particles coated with natural pozzolans. Figure 4 summarizes the WTA acquisition and coating processes.

It should be emphasized that all WTA particles—including those used in the uncoated control series (M1–M4)—underwent the identical pretreatment protocol, i.e., pressurized water washing, oven-drying at 60 °C to constant mass, 24 h immersion in 12 M NaOH solution, and 24 h post-treatment conditioning at 75 °C. The only procedural difference between the control and coated series was the subsequent dry-coating step with natural pozzolan powder. Therefore, any performance differences between the uncoated and coated series can be attributed solely to the presence and type of the pozzolanic coating layer, and not to differences in alkaline pretreatment or thermal history.

The effectiveness of the dry-coating procedure was verified by mass balance: the coated WTA batches were weighed before and after the coating and conditioning steps, confirming the uptake of the pozzolanic powder fraction by the rubber particles. The coated aggregates exhibited a visually uniform and continuous powder layer over the entire particle surface (Figure 3), with no observable agglomerated loose powder after the 75 °C conditioning, indicating stable adhesion of the coating to the alkali-activated rubber surface. It is acknowledged that SEM imaging and EDS elemental mapping of the individual coated particles, which would allow direct quantification of the coating layer thickness and homogeneity, were not performed; this is recognized as a limitation of the present study (Section 4). The successful performance improvements and the densified interfacial zones observed in the composite SEM analyses (Section 3.7) nevertheless provide indirect evidence of the presence and integrity of the coating layer within the composites.

### 2.3. Mix Design, Sample Production and Curing Process

This study involved designing 16 different mixtures for a one-part alkali-activated hybrid binder system using the Taguchi L16 orthogonal array. The experimental setup focused on three key parameters: (i) the surface condition or coating type of waste rubber aggregate, (ii) the replacement rate of waste rubber aggregate (WTA, %), and (iii) the PP fiber content. The coating types included four levels: uncoated, pumice-coated, perlite-coated, and diatomite-coated waste tire aggregates. The WTA ratio was tested at 10%, 25%, 50%, and 75%, while the PP fiber ratio was varied at 0%, 0.5%, 1%, and 2%. Using the Taguchi L16 method enabled efficient investigation of variable effects without the extensive number of experiments required by a full factorial design, providing a more systematic and rational approach. The specific levels used in the experimental design are detailed in Table 4.

Since the L16 orthogonal array is balanced, each factor level appears in four experimental runs and combines equally with other factors’ levels. Consequently, the factor-level values shown in the main-effect plots are calculated as the averages of the four corresponding formulation means. Each of these means was derived from three specimens tested at different ages for each property. Therefore, the plotted coating-, WTA-, and PP-fiber-level values are marginal main-effect means and should not be seen as responses from a single M1–M16 formulation. The error bars indicate the standard deviation of the four formulation means.

Analysis of the mixture compositions showed that the primary components of the binder matrix remained consistent across all test series. Specifically, each mixture included UK at 400 kg/m^3^, cement at 400 kg/m^3^, water at 280 kg/m^3^, PCE at 40 kg/m^3^, and metasilicate at 120 kg/m^3^. The alkali activator was added at 15% of the total binder (UK + CEM II B-S), while PCE was reintroduced at 5% by weight of the binder. In essence, the binder system and activator composition were held constant across all tests, with experimental variation introduced only by adjustments to aggregate and fiber contents. This methodology is crucial for directly correlating differences in mechanical, physical, and durability performance to factors such as coating type, tire waste replacement rate, and PP fiber contribution. The fixed matrix design enables more precise identification of cause-and-effect relationships between microstructure–interface behavior and overall composite performance. The material mixture ratios and material quantities for the experimental study are given in Table 5.

For the coated series, the reported WTA quantities include the adhered pozzolanic coating layer, as the coated aggregates were batched as a single aggregate component after the 75 °C conditioning step.

The reference mix uses only silica sand as the fine aggregate, with no waste tire aggregate or PP fiber added. Its silica sand content was 931.0 kg/m^3^. In the L16 test series, the amount of silica sand was gradually reduced in accordance with the specified WTA ratio, while the amount of waste tire aggregate increased. The table clearly shows that as the WTA ratio rises, silica sand decreases and waste tire aggregate increases. For instance, in the uncoated series, silica sand dropped from 789.0 kg/m^3^ in mixture M1 to 219.1 kg/m^3^ in M4, while waste rubber rose from 32.0 kg/m^3^ to 241.7 kg/m^3^. Similar patterns appear in other series involving pumice-, perlite-, and diatomite-coated aggregates. This demonstrates a characteristic substitution behavior that reduces the mixture’s total mass, owing to the low unit volume weight of the waste rubber aggregate.

When assessed by group, the mixtures include waste rubber aggregates with specific coatings: the M1–M4 series are uncoated; M5–M8 are pumice-coated; M9–M12 are perlite-coated; and M13–M16 are diatomite-coated. Within each group, the WTA ratio and PP fiber content were varied using the Taguchi design to explore their effects. The study aimed to analyze the main effects of waste rubber content, mineral-based coatings, and fiber reinforcement on composite behavior. It is anticipated that porous, siliceous, and natural mineral-based coatings such as pumice, perlite, and diatomite will alter the hydrophobicity of the waste tire surface, improve adhesion between the binder matrix and aggregate, strengthen the interfacial transition zone, and promote a more uniform microstructure. Additionally, PP fibers are expected to reduce brittle failure by bridging cracks and maintaining composite integrity. In conclusion, this mixture design goes beyond simply specifying material ratios; it offers a controlled experimental platform with a fixed binder matrix. This setup facilitates a comparative evaluation of how coating type, waste-tire replacement level, and PP fiber content affect the performance of one-part alkali-activated composites. Utilizing the Taguchi L16 experimental design minimizes the number of experiments while enabling a statistically robust, practically relevant analysis of the multivariable system.

The mixtures are prepared according to the “just add water” approach for one-part alkali-activated systems. In this production approach, liquid alkali solutions are not used; instead, precursor binders and solid activators are first homogenized in dry form, and the reaction begins with the addition of water. Therefore, the mixture procedure has been designed to account for both the mechanical mixing logic of classic mortar preparation standards and the reactive, dry-mixture character of one-part geopolymers. Furthermore, the literature emphasizes that mixing, pre-blending, and curing conditions in one-part alkali-activated materials are decisive for fresh and hardened properties.

Initially, in this phase, a laboratory-type planetary mixer was used to blend the UK cement, solid sodium metasilicate, and silica sand at low speed to form the binding phase. Mixing speeds were set at 140 ± 5 rpm and 285 ± 10 rpm, within the low- and medium-speed ranges specified in ASTM C305 [49] for mortar mixes. First, all dry binder materials and fine aggregate were combined at 140 rpm for 2 min to create a uniform dry blend. Subsequently, waste rubber aggregates coated with pumice, perlite, or diatomite—tailored to each test series—were incorporated into the dry mix and blended for an additional minute at the same speed. This initial dry mixing step aimed to ensure a more even distribution of the solid activator, uniform dispersion of coated rubber particles, and to prevent local clumping or irregular reaction zones when water was added.

In the second phase, the fluidized water was first homogenized in a container, then carefully added to the dry mixture. During water addition, the mixer operated at a low speed, and the liquid was introduced over approximately 30–60 s. Afterward, the mixture was briefly homogenized at low speed, then mixed at 285 rpm for 2 min. The mixing was then halted, and any material stuck to the container and paddle surfaces was scraped off. The mixture was allowed to rest for about 90 s, with manual scraping for the first 15 s, after which the remainder was left undisturbed. This process aligns with the intermediate resting-and-scraping approach outlined in ASTM C305 [49], promoting proper particle wetting and rheological stability, particularly in one-part systems with solid activators.

PP fibers were introduced at the final stage of mixing to prevent fiber matting and uneven distribution. The literature on fiber-reinforced mortar suggests that gradually adding fibers helps minimize agglomeration. Therefore, PP fibers were not added all at once but sprinkled slowly over about 30–45 s, then mixed for 1 min at 140 rpm, followed by another minute at 285 rpm. The total mixing time was approximately 7–8 min. This approach promotes a more uniform fiber distribution within the matrix and reduces heterogeneity caused by waste-rubber aggregates. Since rubber-based aggregates tend to form weaker interfaces with the cement/binder matrix, controlled mixing and thorough homogenization are vital for these composites. The mixing process has been refined to maintain a homogeneous one-part geopolymer matrix with a solid activator, ensure the uniform distribution of coated waste-tire aggregates, and enable the incorporation of PP fibers without clumping. This approach aims to ensure that any performance variations between experimental series are due to the primary parameters changed in the mixture design.

The specimens underwent a gentle thermal curing process at 45 °C for 24 h immediately after molding. This process was selected to accelerate the water-induced dissolution and gelation reactions in one-part alkali-activated systems, facilitating early matrix formation. The 45 °C temperature effectively enhances reaction kinetics without risking excessive water loss, microstructural damage, or unwanted thermal effects. As a result, a 24 h cure at this temperature was chosen as the optimal, controlled initial curing method. It helps promote early strength development while maintaining the material’s structural stability over time.

### 2.4. Test Methods

To assess the workability of the mixtures in their fresh state, a spread diameter test was performed on a flow table in accordance with ASTM C1437 [50]. The freshly prepared mortar was placed in two layers into a flow mold at the center of a clean, dry flow table. After placing the first layer, about 25 mm thick, it was tamped 20 times; the second layer was then tamped 20 times. The mold’s top surface was smoothed with a trowel. After mixing, the mold was lifted vertically within 1 min, and the table was dropped 25 times over 15 s. The spread diameter was then measured in four directions on the table, and the average of these measurements was noted as the mixture’s spread diameter in millimeters.

The apparent porosity, water absorption, and oven-dried density were measured on cubic specimens (50 × 50 × 50 mm) in accordance with ASTM C642 [51]. After a 24 h thermal cure at 45 °C, the specimens were kept under laboratory conditions for up to 28 days. Their dry masses were then determined by oven-drying to constant weight. The specimens were saturated with water, and their saturated-surface-dry mass and suspended water mass were recorded. Using these measurements, the water absorption rate, apparent porosity, and oven-dry density were calculated. These parameters helped assess the pore structure, matrix density, and water resistance of the one-part geopolymer composites.

To evaluate the mechanical performance of the mixtures, flexural and compressive strength tests were performed in accordance with ASTM C348 [52] and ASTM C349 [53]. Prismatic specimens measuring 40 × 40 × 160 mm were prepared and subjected to a 24 h mild thermal cure at 45 °C, then maintained under laboratory conditions until the designated test ages. Mechanical properties were assessed on days 7, 28, 90, and 180. Flexural strength was measured using a three-point bending test on prismatic specimens, with load applied at the center and both ends supported. The broken halves from the flexural test were used for compressive strength testing, adhering to the same standard approach. By analyzing the flexural and compressive strengths from the same specimen, we systematically investigated the short- and long-term mechanical behavior of one-part geopolymer composites. This approach was selected to optimize specimen use and reliably monitor strength development over time.

The fracture parameters of the 28-day-old specimens were determined using a three-point bending test on prismatic samples measuring 25 × 60 × 300 mm, following the methodology of previous studies. Tests were conducted under displacement control at a loading rate of 0.1 mm/min. During loading, both the applied load and mid-span deformation were recorded simultaneously; the test was terminated when the post-peak load had decreased by approximately 10% from the maximum value, i.e., to about 90% of the peak load. The load–deformation curves were used to extract the peak load, peak deformation, and toughness. The peak load is the maximum load the specimen can withstand; the peak deformation is the mid-span displacement at this load; and toughness is the energy absorbed, calculated as the area under the load–deformation curve up to the defined termination point. It is acknowledged that this termination criterion captures only the early post-peak portion of the material’s behavior; the reported toughness values therefore represent a defined, consistently applied comparative measure of early post-cracking energy absorption rather than the total fracture energy. As the same criterion was applied to all mixtures, the comparative evaluation and ranking of the mixtures remain valid, while the absolute toughness values should be interpreted as lower-bound estimates. This is acknowledged as a limitation in Section 4. This approach evaluates strength while also offering insights into crack propagation, deformation capacity, and post-failure energy dissipation in the composite.

To assess capillary water absorption, tests were conducted on 28-day-old cubic specimens (50 × 50 × 50 mm) in accordance with TS EN 1015-18 [54]. Before testing, specimens were oven-dried at 50 ± 5 °C until a stable weight was reached, then cooled, and their initial dry masses were recorded. Only the bottom surface, which contacts water, was left exposed; all other surfaces were sealed to direct capillary flow in one direction. The specimens’ lower surfaces were immersed in water, maintained at about 5–10 mm. At set intervals, they were removed, surface water was wiped off, and the mass increase was measured. The contact surface area was used to normalize the mass changes and determine capillary water absorption. This procedure helped evaluate pore connectivity, capillary pore structure, and water-migration tendencies in one-part geopolymer composites.

To assess high-temperature performance, cubic specimens measuring 50 × 50 × 50 mm were subjected to 200, 400, and 600 °C after the standard 28-day curing period. The specimens were placed in a muffle furnace under an air atmosphere, heated at 5 °C/min to the target temperature, then maintained at that temperature for 2 h to ensure even thermal effects across the cross-section. After heating, they were naturally cooled to room temperature to prevent thermal shock damage. Once cooled, their final masses were measured and compared to initial weights to determine mass loss. The compressive strength was then tested to evaluate the effects of high-temperature exposure on both mass loss and mechanical properties. This method aligns with standard procedures in the literature for studying the thermal resistance of alkali-activated and geopolymer composites, enabling comparison of matrix degradation, water loss, microcracking, and changes in load capacity after high-temperature treatment.

Freeze–thaw resistance was evaluated using a modified freeze–thaw procedure adapted from the principles of ASTM C666 [55]. It should be noted that the applied regime—a 12 h cycle consisting of a 7 h hold at −20 °C and a 5 h hold at +4 °C, applied for up to 90 cycles—deviates from the rapid cycling conditions defined for Procedures A and B of ASTM C666, and the test is therefore reported as a modified protocol rather than a standard ASTM C666 test. After the standard 28-day curing period, specimens were placed in a freeze–thaw chamber immersed in water. Testing was interrupted after 30, 60, and 90 cycles; at each interval, specimens were removed, surface moisture was wiped off, and masses were recorded. Mass loss was calculated from the initial and post-cycle masses, and the residual compressive strength was determined at each interval. The relative dynamic modulus of elasticity and the durability factor, as defined in ASTM C666, were not measured; instead, mass loss and residual compressive strength were adopted as damage indicators, an approach widely employed in freeze–thaw studies of alkali-activated and geopolymer composites.

To assess chemical resistance against MgSO_4_ and H_2_SO_4_, 50 × 50 × 50 mm cubic specimens were immersed in 5 wt% MgSO_4_ and 5 wt% H_2_SO_4_ solutions (corresponding to approximately 0.42 mol/L and 0.51 mol/L, respectively) for 120 days following the 28-day standard curing period, using a protocol adapted from ASTM C267 [56]. For each solution, the specimens were fully immersed in polyethylene containers (450 × 300 × 250 mm) filled with 23 L of test solution, with the solution level maintained at least 10 mm above the specimen surfaces, and the containers were kept at laboratory ambient temperature (20–25 °C). The solutions were renewed weekly to maintain a stable concentration throughout the exposure period. The pH of the solutions was not monitored during exposure; based on the nominal concentrations, the initial pH of the H_2_SO_4_ solution is expected to be below 1, whereas that of the MgSO_4_ solution is expected to be near-neutral to slightly acidic. Before immersion, the initial masses of the specimens were recorded. At the end of the 120-day exposure, the specimens were removed from the solutions, rinsed gently with tap water, surface-dried with a damp cloth, and their final masses were measured to determine the mass change. Compressive strength tests were then performed on the exposed specimens. To separate chemical degradation from the ongoing strength development due to continued curing, the residual strengths after exposure were evaluated relative to the strengths of age-matched companion specimens cured under standard conditions for the same total duration, rather than relative to the 28-day values. Unlike ASTM C1012 [57], which focuses on dimensional changes, sulfate and acid effects were assessed in this study through strength and mass change.

It should be noted that the coating powder applied at 40 wt% of the rubber mass introduces additional pozzolanic solids into the composite in proportion to the WTA content (theoretically up to ~130 kg/m^3^ in the 75% WTA series). However, three points should be considered: (i) the coating layer adheres firmly to the rubber surface after the 75 °C conditioning and functions as an integral part of the aggregate particle at the interfacial transition zone, rather than as free binder; (ii) any loosely bound fraction detached during mixing acts as reactive aluminosilicate fines that are chemically compatible with the hybrid binder system; and (iii) the base binder proportions (FA, cement, sodium metasilicate, water and PCE) were kept strictly identical in all 17 mixtures. Even under the conservative assumption that the entire coating fraction behaves as additional reactive solids, the effective water-to-solids ratio ranges only between 0.266 and 0.304 across all mixtures—remaining within the typical range for one-part alkali-activated systems. The scaling of the coating-derived powder fraction with WTA content is nevertheless acknowledged as an inherent feature of the proposed approach and is addressed as a limitation in Section 4.

All mixtures were cast and compacted using the identical procedure (identical mold-filling and vibration steps and durations) to ensure comparable compaction across the series, and the workability of every mixture was verified by the flow-table test (Section 3.1). Fresh densities and air contents of the mixtures were not systematically measured; this is acknowledged as a limitation (Section 4), given the differences in flowability and in the water absorption characteristics of the coating materials. It should also be noted that the oven-dried coated aggregates absorb part of the mixing water through their porous pozzolanic coating layer—whereas the rubber particles themselves are non-absorbent—so that the effective water available for the binder is somewhat lower than the nominal mixing water in the coated series. This effect is inherently coupled to the coating approach and is addressed together with the water-to-solids discussion in Section 2.3.

For each formulation and test condition, three specimens were tested for every destructive property, and the results were expressed as the mean ± standard deviation at the formulation level. Each formulation was independently produced in a total of (45 pieces) batches. In the Taguchi factor-level plots, each point shows the mean of the four L16 formulation means at that factor level. The error bars depict the standard deviation among these four means, indicating the variability of the formulations at each level; they do not show standard error or the within-formulation specimen standard deviation. Flexural and compressive strength tests were carried out using a UTEST testing system with 15 kN flexural and 250 kN compressive loading capacities, at the loading rates specified in ASTM C348 [52] and ASTM C349 [53], respectively. The three-point bending fracture tests were performed using a Shimadzu Autograph AGS-X universal testing machine under displacement control at a rate of 0.1 mm/min. High-temperature exposure was applied in a PROTHERM PLF160/9 high-temperature furnace, and freeze–thaw cycling was performed in a custom-designed freeze–thaw chamber.

## 3. Results and Discussion

### 3.1. Fresh Properties

The workability tests showed that the spread diameter of the reference mixture was 202 mm, with noticeable differences depending on whether WTA was used and on the type of pozzolanic surface treatment. Mixtures with uncoated WTA had a 12.38% smaller spread, measuring 177 mm, due to higher internal friction from the rough, hydrophobic surface of rubber aggregates produced by mechanical fragmentation [58,59]. When assessing how natural pozzolanic coatings affect fresh-mix performance, the pumice-modified series showed a spread diameter of 193 mm, only 4.46% less than the reference, indicating the best workability among the coated groups. This beneficial physical effect from pumice can be attributed to the coating process, which partly reduces friction on the rubber surface and enhances flowability by creating a ball-bearing effect. Conversely, the perlite-coated series experienced a 17.82% reduction compared to the reference, while the diatomite-coated series saw the most significant decrease of 39.11%, with a spread diameter of 123 mm. The increase in viscosity is mainly due to diatomite’s biogenic origin, its highly porous structure, and very high specific surface area [60]. This high specific surface area rapidly absorbed the free water required for the system’s polymerization reaction, thereby significantly reducing the flowability of the single-component geopolymer matrix.

**Figure 5:**
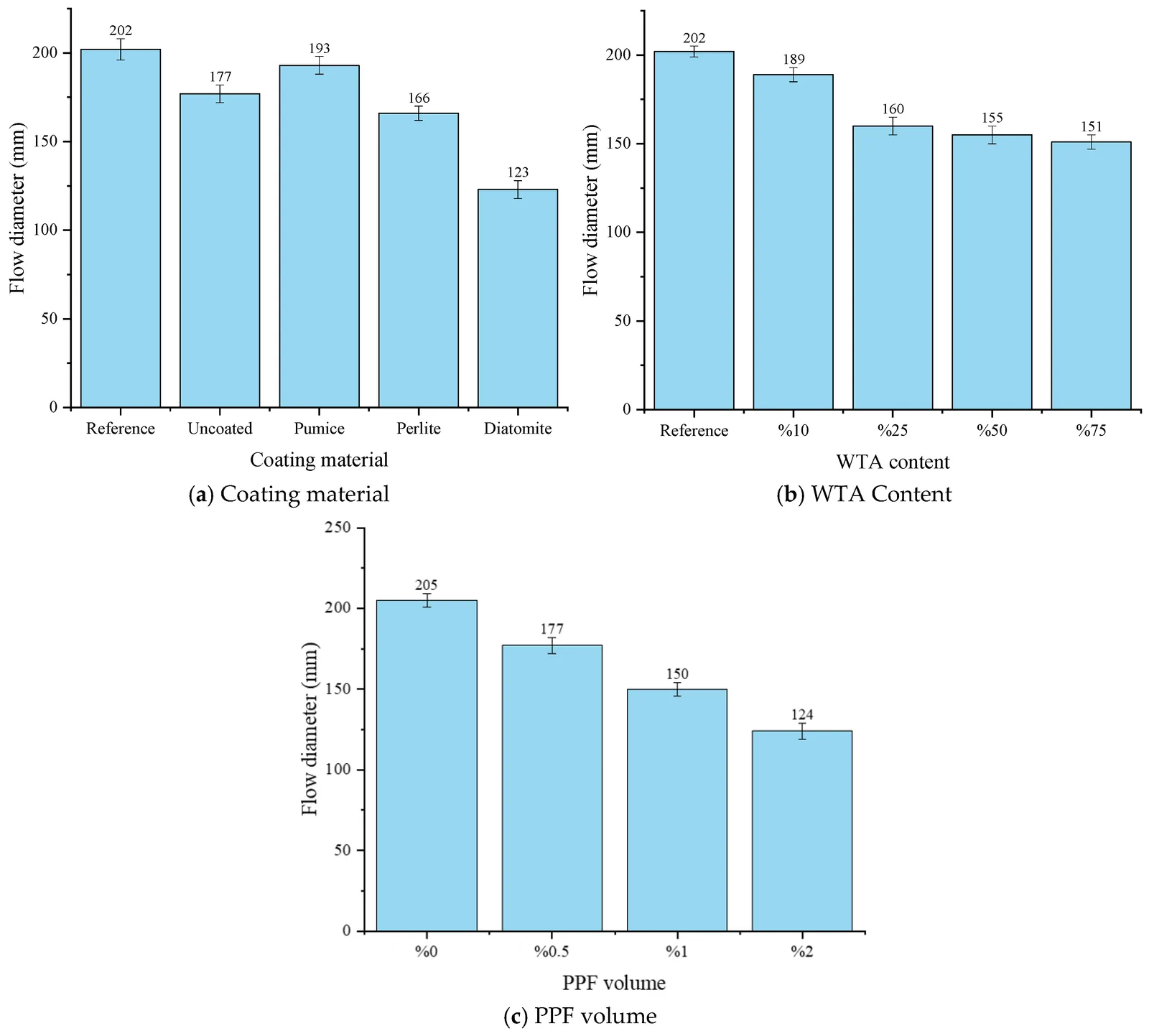
Fresh state properties of hybrid geopolymer composites.

Replacing silica sand with WTA in mixtures, especially at higher levels, systematically reduces the spreadability of the fresh hybrid matrix (Figure 5b). The spread diameter was 202 mm in the reference mixture. Still, it decreased by 6.44% to 189 mm at 10% WTA substitution. When the ratio increased to 75%, the diameter decreased further to 151 mm, a significant 25.25% reduction from the reference. Comparing the lowest and highest waste additions within the same Taguchi series, mixtures with 75% WTA showed 20.11% lower fluidity than those with only 10% WTA. This increased cohesion in the fresh mortar is due to WTA’s lower specific gravity (1.09), which is much lower than that of silica sand. The expansion of the total particle surface area created by the lighter rubber aggregates within the system during the same volumetric displacement directly increased interparticle friction and restricted the mixture’s ability to spread under its own weight [61]. Furthermore, the hydrophobic nature of the waste rubber particles, which creates an air-entraining effect in fresh mortar and hinders the free flow of the matrix, is fully consistent with findings reported in the relevant literature [62]. The increased demand for specific surface area, coupled with the rising rubber volume, thickened the fresh geopolymer mortar, thereby increasing the mechanical energy required for homogeneous settlement.

It has been found that the PP fibers added to the system to enhance toughness, energy absorption, and crack bridging in the developed hybrid composites significantly reduce the material’s fresh workability. In the experimental design, the average crack propagation diameter, recorded at 205 mm in the fiber-free group (0% PPF), decreased progressively and notably with the incorporation of PP fibers at 0.5%, 1%, and 2%. Compared to the fiber-free matrix, adding 0.5% PP fibers reduced the spread diameter by 13.66% to 177 mm, while adding 2% PP fibers reduced it by 39.51% to 124 mm. This physical behavior is likely due to PP fibers randomly distributed within the fresh mortar, forming a three-dimensional rigid network that significantly increases the mixture’s yield stress [63,64]. Unlike hydrophilic natural fibers, PP fibers do not absorb mixing water; however, their high specific surface area and the rigid three-dimensional network they form within the fresh mortar increase the yield stress and reduce the free water available for flow, thereby decreasing workability. Research also confirms that the high cohesion and tendency of organic polymer fibers to agglomerate within the fresh matrix are the main factors contributing to reduced flowability [65]. In single-component systems with a solid-activator formulation, the combined effect of increasing fiber content and waste rubber aggregate has led the hybrid geopolymer matrix to reach its maximum viscosity, resulting in a sharp reduction in the spread diameter.

### 3.2. Physical Properties

**Figure 6:**
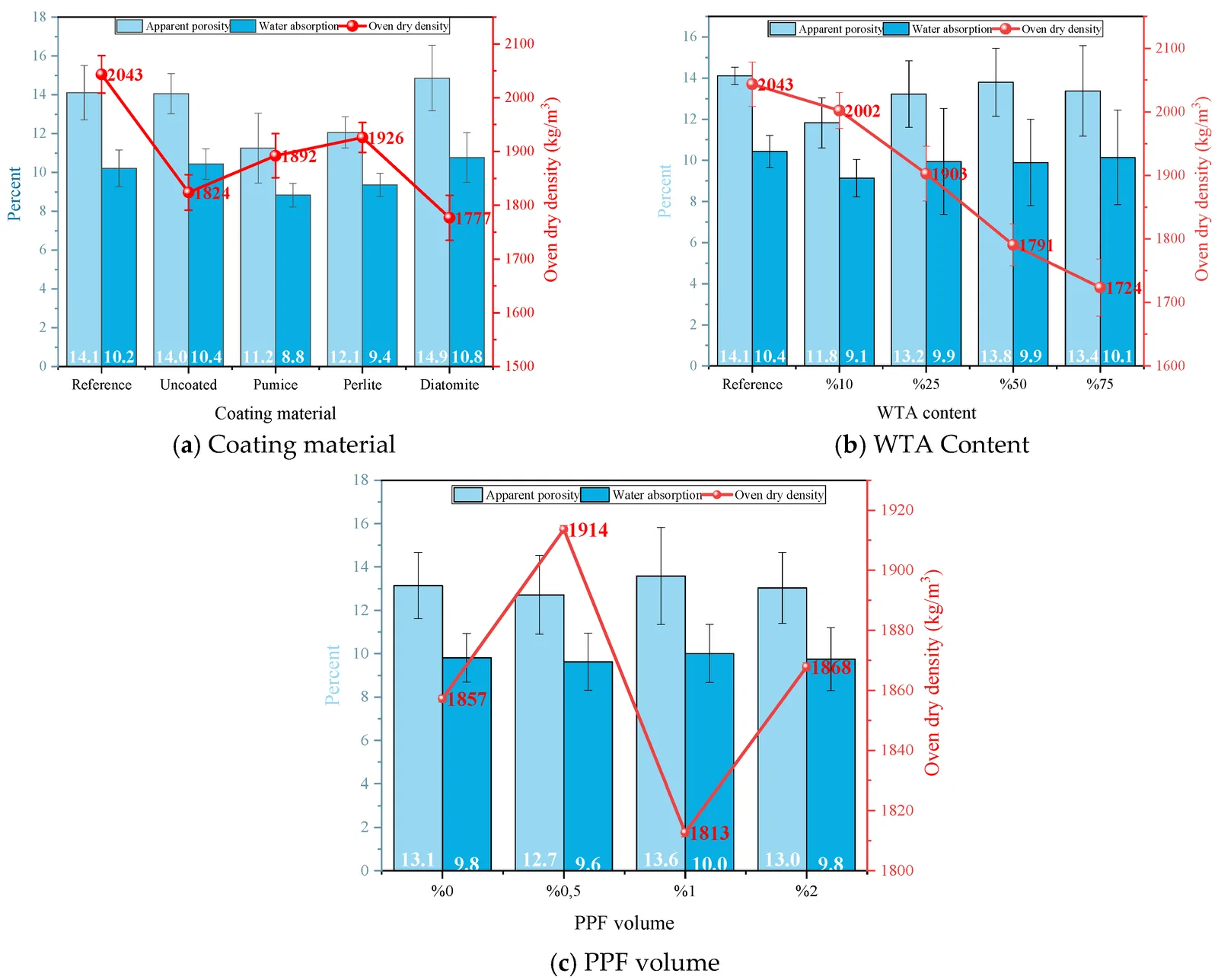
Physical properties of hybrid geopolymer composites.

Evaluation revealed that the apparent porosity and water absorption values in the reference mixture, at 14.1% and 10.2%, respectively, remained nearly the same at 14.0% and 10.4% with uncoated WTA, indicating no significant difference (Figure 6a). However, in the uncoated series, the oven-dry density dropped by 10.72% from the reference, reaching 1824 kg/m^3^. When examining the impact of surface modifications on the microstructure, the pumice-coated series showed the best performance, with apparent porosity decreasing by 20.57% to 11.2% and water absorption decreasing by 13.73% to 8.8%. This physical improvement is attributed to natural pozzolanic powders like pumice and perlite, which are rich in amorphous silica and possibly densify the ITZ through secondary reaction products consistent with N-A-S-H- and C-(A)-S-H-type gels during alkali activation. Conversely, the diatomite-coated series showed a 5.67% increase in apparent porosity to 14.9%, while density sharply declined by 13.02% to 1777 kg/m^3^. Consistent with the existing literature, this increased porosity stems from diatomite’s inherent high porosity and its rapid water absorption during polymerization, which disrupts reaction kinetics and traps air voids within the matrix due to settlement difficulties [22,66].

When examining the systematic effect of the WTA replacement ratio on the matrix’s density and porosity parameters, it was found that increasing the rubber volume significantly decreased the weight of the composites (Figure 6b). The density of the reference mixture, which was 2043 kg/m^3^, dropped by 2.01% to 2002 kg/m^3^ with a 10% WTA substitution, and decreased further to 1724 kg/m^3^, representing a substantial 15.61% reduction, when the substitution ratio increased to 75%. The main reason for this structural density reduction is that the specific gravity of WTA (1.09) is much lower than that of the reference quartz sand (2.70). A notable microstructural observation is that replacing 10% of WTA decreased the apparent porosity by 16.31%, to 11.8% relative to the reference mixture. This result is attributed to the replacement of part of the water-absorbing matrix–aggregate system with non-absorbent, impermeable rubber particles, which reduces the volume of water-accessible open pores per unit volume of the composite [67], rather than to any void-filling action (the rubber particles, at 0.5–4 mm, are far coarser than capillary pores). When the WTA ratio was increased from 10% to 75% volumetric replacement, porosity began to rise, approaching the reference value and reaching 13.4%, while the water absorption rate increased from 9.1% to 10.1%. This negative physical effect of increased rubber volume is caused by the hydrophobic nature of the rubber particles, which trap more air in the fresh mixture and form a porous interfacial transition zone (ITZ) with weak microcracks between the hybrid binder matrix and the rubber surface [68,69].

Analyzing the impact of PP fiber content on composite pore structure and density reveals that fiber addition improves the matrix up to an optimal point, beyond which it harms physical properties (Figure 6c). The fiber-free group (0%) had an average oven-dry density of 1857 kg/m^3^, which increased by 3.07% to 1914 kg/m^3^ with 0.5% PP fiber. At the same time, apparent porosity slightly decreased from 13.1% to 12.7% (a 3.05% reduction). This marginal improvement is possibly associated with the well-dispersed 0.5% fibers restraining autogenous-shrinkage-induced microcracking during geopolymerisation, thereby slightly reducing the open porosity of the matrix; the fibers themselves do not densify the matrix. However, increasing fiber content to 1% and 2% reduced the density to 1813 kg/m^3^ and 1868 kg/m^3^, respectively, while apparent porosity reached a maximum of 13.6% at 1% fiber content (a 3.82% increase over the fiber-free mix). This reversal is explained by the tendency of high volumes of fibers to form agglomerates in fresh mortar, which significantly reduce workability, impede effective compaction, and trap interconnected macro-air voids within the matrix [70,71].

### 3.3. Mechanical Properties

#### 3.3.1. Compressive Strength

**Figure 7:**
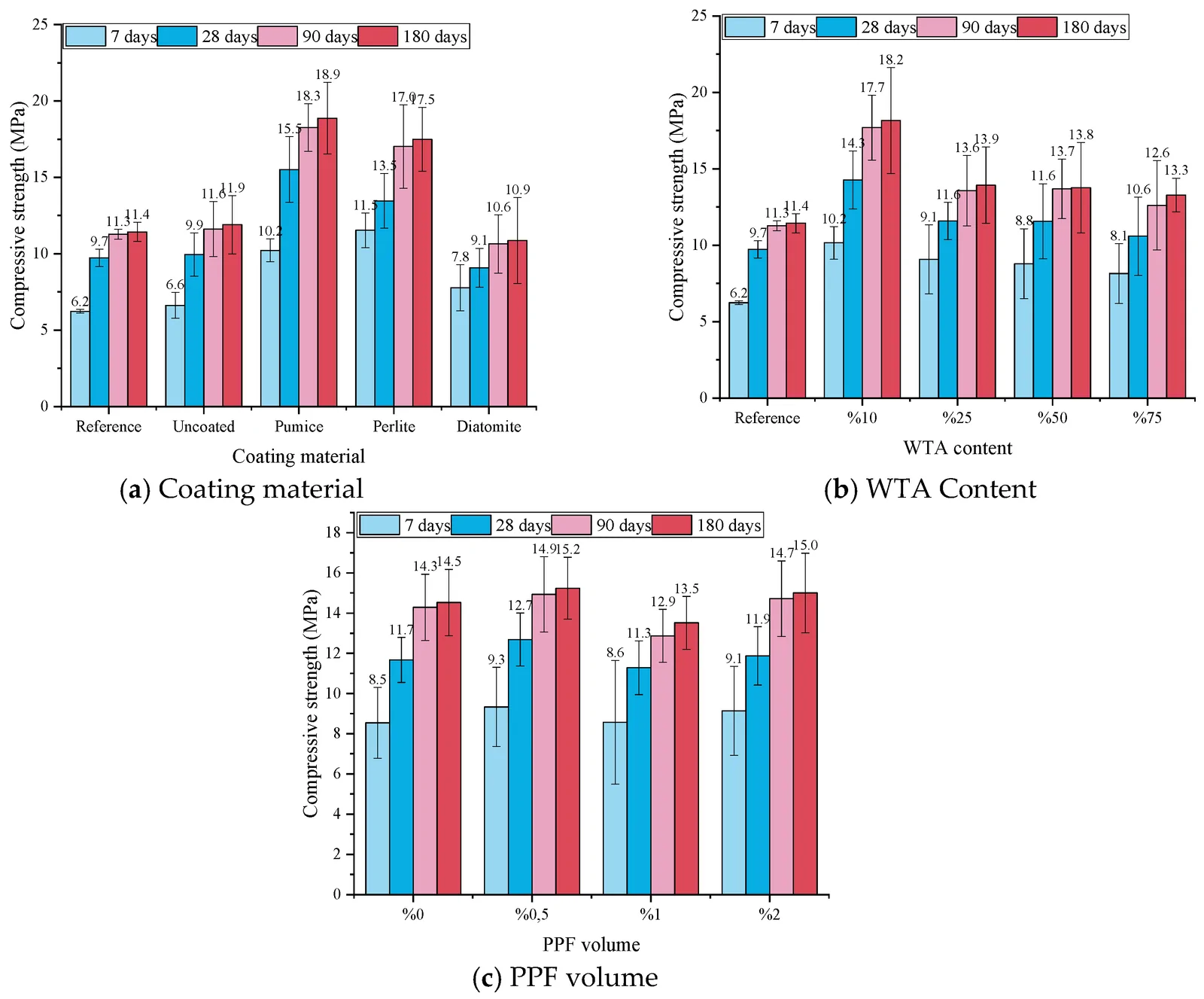
Compressive strength of hybrid geopolymer composites.

An analysis of the time-dependent compressive strength data in Figure 7 shows that the single-component hybrid geopolymer composites steadily increased in strength over curing periods from 7 to 180 days. This consistent pattern of ongoing improvement aligns with the concurrent formation of C-(A)-S-H and N-A-S-H hybrid gels, which result from alkali activation of key binders like cement and fly ash, leading to progressive densification of the matrix over time [72,73], in the reference mixture, which served as the baseline for the experimental setup and contained no WTA or PP fibers, the 28-day compressive strength was measured at 9.7 MPa. This value increased by 17.5% to 11.4 MPa after 180 days.

When analyzing the variation in the WTA replacement ratio (Figure 7b) within the mixtures, it was observed that the 28-day average compressive strength of the series with 10% WTA by volume in the L16 Taguchi orthogonal array reached 14.3 MPa. This marks a 47.4% increase over the reference mixture. The elevated strength was attributed to the concurrent main-effect contributions of the pozzolanic coatings in the 10% WTA group and of the polypropylene fibers integrated into the system, both of which enhanced the load-bearing capacity. However, calculations showed that increasing the WTA replacement ratio from 10% to 75% resulted in a consistent 25.9% decrease in 28-day compressive strength, reducing it to 10.6 MPa. This notable loss of strength at high rubber volumes is primarily due to the rubber particles’ elastic modulus being much lower than that of the cementitious matrix and to stress concentrations that form around the rubber particles under load, which cause early microcrack development [31,61]. Furthermore, the hydrophobic nature of the rubber has created weak adhesion in the interface transition zone (ITZ), thereby limiting the matrix’s load-transfer capacity and weakening the composite’s load-bearing skeleton [30,74].

Regarding 28-day compressive strength, 0.5% PP fiber was the most effective dosage among those tested (Figure 7c). However, this finding is specific to compressive performance and should not be extended to flexural strength or fracture toughness, where higher fiber contents showed better results. Whilst the 28-day compressive strength in the fiber-free (0% PPF) series was recorded as 11.7 MPa, the incorporation of 0.5% PP fiber into the mixture increased the compressive strength by 8.5%, raising it to 12.7 MPa. This specific mechanical improvement has been attributed to the fibers forming a three-dimensional network within the matrix, which bridges microcracks (fiber bridging), absorbs fracture energy, and delays brittle fracture by distributing internal stresses more uniformly throughout the system [75,76]. Conversely, increasing the fiber content to higher volumetric levels, such as 1% and 2%, resulted in compressive strengths of 11.3 MPa and 11.9 MPa, respectively. This observed reversal has been attributed to the tendency of fibers at high dosages to agglomerate within the fresh mortar, thereby creating undesirable, weak air voids and disrupting the continuity of the hybrid matrix [63,77].

When comparing different coatings to address the weak ITZ problem, the pumice-modified series showed the best performance (Figure 7a). The uncoated WTA series had a 28-day compressive strength of 9.9 MPa, which increased significantly by 56.6% to 15.5 MPa with the pumice coating. Pumice’s significant chemical and physical contributions are confirmed by its amorphous silica-rich structure, which interacts in an alkaline environment, chemically repairing the interface transition zone (ITZ) through secondary reactions that form C-(A)-S-H and N-A-S-H gels. Additionally, its rough surface enhances mechanical interlocking [78,79,80]. The perlite-coated series also showed similar pozzolanic reactivity, demonstrating effective performance with a strength value of 13.5 MPa (a 36.4% increase over the uncoated series). Conversely, the 28-day strength in diatomite-coated mixtures decreased by 8.1% compared to the uncoated series, reaching 9.1 MPa. This restrictive effect of diatomite on the mechanical structure results from the dust’s biogenic origin, extremely fine pore structure, and high specific surface area, which quickly absorbs free water—crucial for polymerization kinetics—thus impeding the matrix’s settlement and leaving unreacted, porous, weak phases within the system [81,82].

The effectiveness of pumice in terms of compressive strength mainly stems from the material’s volcanic origin and its chemical makeup, which is especially rich in amorphous silica. This amorphous silica exhibits high pozzolanic reactivity in an alkaline environment, leading to secondary reactions with cement and fly ash precursors. As a result of this activation, additional C-(A)-S-H and N-A-S-H gels form in dense layers at the interface transition zone (ITZ) between the rubber aggregate and the matrix, which is usually quite weak. These secondary hybrid gels chemically mend the weak interface caused by WTA. At the same time, the pumice’s lightweight, rough, and porous surface structure also improves mechanical interlocking with the matrix, thereby increasing load-bearing capacity.

In contrast, diatomite, although it is a silica-rich sedimentary rock, originates from biogenic sources and features an extremely fine pore network with a very high specific surface area. The main reason for the decrease in diatomite’s compressive strength is the excessive water demand resulting from its high surface area. It was observed that diatomite powders added to the system quickly absorbed free mixing water, which is essential for the reaction and alkali activation in the one-part geopolymer system. This rapid water uptake interfered with polymerization in the matrix, limiting gel formation and resulting in unreacted, weak phases in the system. Additionally, the absorption of free water by the diatomite sharply increased the viscosity of the fresh mortar, hindering effective compaction of the matrix and reducing workability. Undesirable macro-air voids became trapped within the insufficiently settled matrix, thereby increasing porosity and disrupting matrix continuity, which directly weakened the load-bearing skeleton. It has been confirmed that stress concentrations around these voids and unreacted weak phases under load trigger early microcrack formation, significantly reducing the compressive strength of the material in the diatomite-coated series.

#### 3.3.2. Flexural Strength

**Figure 8:**
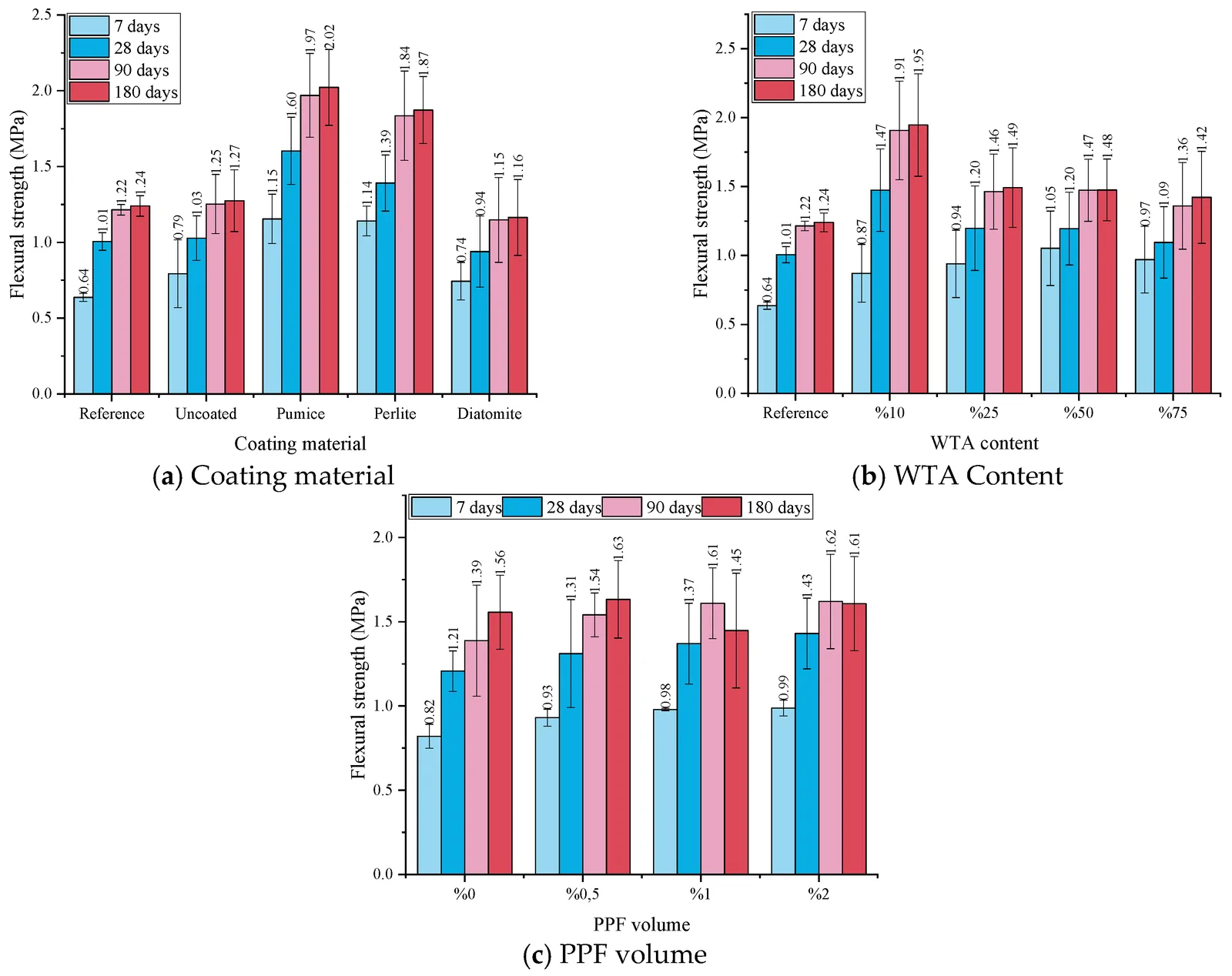
Flexural strength of hybrid geopolymer composites.

An analysis of the flexural strength data shown in Figure 8 indicates a steady increase in strength across all hybrid geopolymer composite series during curing periods from 7 to 180 days. For instance, while the flexural strength of the reference mixture was 1.01 MPa at 28 days, it rose by 22.7% to reach 1.24 MPa at 180 days. This ongoing trend of time-dependent improvement is attributed to the maturation of three-dimensional C-(A)-S-H and N-A-S-H hybrid gels within the matrix, resulting from the alkali activation of fly ash and cement precursors, which enhances the matrix’s capacity to resist tensile stresses.

Figure 8a shows that the pumice-coated series achieved the best results in addressing poor adhesion. After 28 days of curing, the flexural strength of the reference mixture was 1.01 MPa. It remained stable at 1.03 MPa in the uncoated series, but in the pumice-coated series, it increased sharply by 58.4% to 1.60 MPa. Likewise, the perlite-coated series showed a 37.6% increase in strength over the reference, reaching 1.39 MPa. This enhanced mechanical performance of the pozzolanic coatings is mainly due to the amorphous, silica-rich structures of pumice and perlite, which induce secondary reactions in an alkaline environment. These reactions fill the interface transition zone (ITZ) between the waste tire aggregate and the matrix with additional products, thereby strengthening the mechanical interlock both physically and chemically [83,84]. On the other hand, the 28-day flexural strength in the diatomite-coated series decreased by 6.9% compared to the reference, to 0.94 MPa. This decrease is explained by diatomite’s very high specific surface area, which rapidly absorbs the free water required for polymerization, resulting in unreacted, weak air voids within the matrix.

Examining the impact of the WTA replacement ratio on matrix stiffness and flexural behavior (Figure 8b) reveals that the material’s flexibility and fracture resistance improve markedly up to an optimal volumetric replacement level. Data show that replacing 10% of the aggregate with WTA increases the 28-day flexural strength by 45.5% relative to the reference mixture, to 1.47 MPa. The presence of low-elastic-modulus rubber particles acts as a crack-arresting barrier within the matrix at low, controlled volumes, enhancing the material’s energy absorption. This aligns well with microstructural findings documented in the literature [85]. However, increasing the WTA replacement ratio to 75% reduced flexural strength to 1.09 MPa, a 25.8% decrease relative to the 10% replacement series. This loss of load-bearing capacity at high rubber volumes has been linked to the increased hydrophobicity of the particle surface, which forms a weak bond (ITZ) with the matrix, and to the inability of tensile stresses generated under bending load to be transferred evenly from the matrix’s underlying fibers to the aggregate [85].

**Figure 9:**
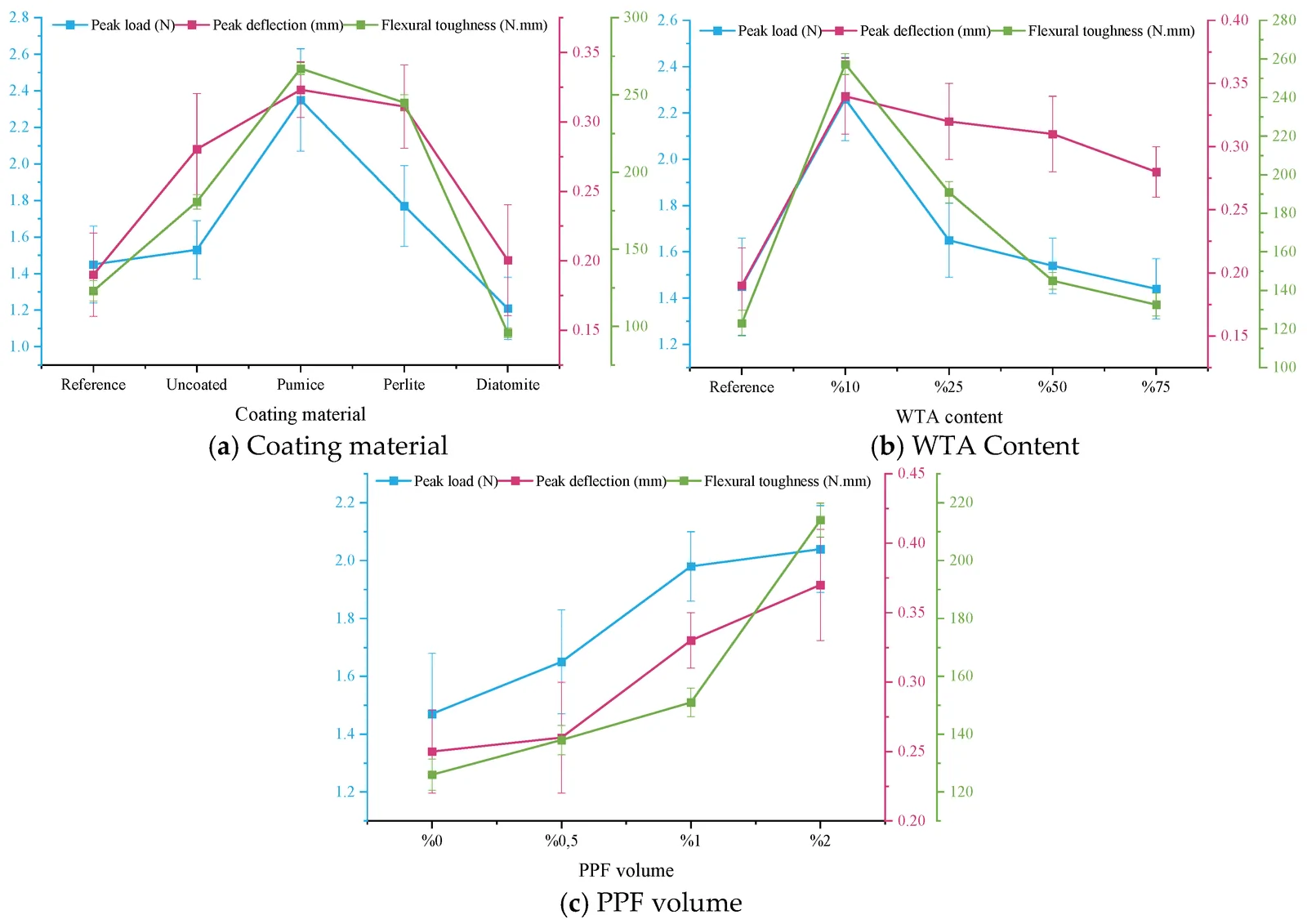
Fracture properties of hybrid geopolymer composites.

When examining the fracture characteristics (peak load, peak deformation, and flexural toughness) shown in Figure 9a, it is clear that the brittle fracture behavior of the reference mixture is significantly transformed into a more ductile, energy-absorbing behavior with the addition of WTA and pozzolanic coatings to the system. In the series with uncoated WTA, peak deformation and fracture toughness improved notably compared to the reference mixture, thanks to the rubber’s flexibility, with peak deformation reaching 180 N·mm (a 38.4% increase in toughness). In comparison, the peak load-carrying capacity remained steady at around 1.5 N due to poor interfacial contact (ITZ) mismatch. When assessing the impact of surface modifications on mechanical and fracture properties, the pumice-coated series performed best. In pumice-coated composites, peak load reached 2.35 N, peak deformation was 2.4 mm, and flexural toughness was 250 N·mm—a remarkable 92.3% increase compared to the reference. This structural advantage stems from amorphous silica-rich pumice undergoing secondary pozzolanic reactions in an alkaline environment, consistent with the formation of additional C-(A)-S-H- and N-A-S-H-type reaction products that chemically repair the interface between the rubber and the matrix, thereby enhancing load transfer [86]. Conversely, in the diatomite-coated series, fracture toughness decreased below the reference level, to 110 N·mm, due to increased porosity and high water demand, which disrupted matrix continuity.

When exploring the effects of WTA substitution ratio on the fracture toughness and load-deformation behavior of the matrix (Figure 9b), it was found that the volumetric substitution level optimizes energy absorption up to a certain point. The average flexural toughness, measured as 130 N · mm in the reference mixture, increased significantly by 84.6% with 10% WTA substitution, reaching 240 N · mm. The maximum load (2.25 N) and maximum deformation also peaked at this mixture. The primary physical mechanism behind this increased toughness is that the flexible rubber particles act as energy-absorbing barriers (crack arresters) within the matrix in controlled, small volumes, slowing down the growth of microcracks. However, it was also observed that increasing the WTA substitution to very high levels, such as 75%, systematically weakened the composite’s load-bearing capacity, with the peak load dropping to 1.45 N and the toughness to 140 N · mm. This aligns with research showing that as the hydrophobic particle surface area increases with higher rubber content, mechanical bonding at the interface transition zone (ITZ) weakens (debonds), disrupting stress transfer under bending loads and reducing fracture energy [87].

When evaluating the contribution of PP fiber content to the fracture energy and toughness parameters of the developed hybrid composites (Figure 9c), a clear and proportional increase in structural strength was observed between the volume of synthetic fibers added and the fracture properties. The peak load of 1.45 N, peak deformation of 1.35 mm, and flexural toughness of 130 N·mm in the fiber-free (0% PPF) mixture rose to peak values of 2.05 N, 1.9 mm, and 215 N·mm, respectively, when the PP fiber ratio was increased to 2%. Notably, adding 2% PP fiber increased the fracture toughness by 65.3% compared to the fiberless matrix, providing clear evidence that the material’s post-cracking load-carrying capacity was significantly enhanced. The formation of a three-dimensional scaffold network through the random distribution of high-tensile-strength polymeric fibers within the fresh matrix, combined with physical bridging (fiber bridging) and fiber pull-out mechanisms that maximize the fracture energy absorbed during bending, transformed the composite from a brittle to a highly ductile fracture phase [88,89].

#### 3.3.3. Fracture Properties

When evaluating the effect of PP fiber content on the flexural strength and crack-bridging mechanism of the composites (Figure 8c), it was observed that increasing the fiber volume enhanced mechanical performance in a steady, straightforward manner. The average flexural strength at 28 days, which was 1.21 MPa in the fiber-free group (0% PPF), rose by 8.2% to 1.31 MPa with the addition of 0.5% PP fibers; by 13.2% to 1.37 MPa with 1% PP fibers; and peaked at 1.43 MPa with an overall increase of 18.1% after incorporating 2% PP fibers. This systematic improvement in structure resulted from the high-strength synthetic fibers forming a three-dimensional, randomly oriented reinforcement network within the fresh matrix, effectively bridging microcracks formed under bending (fiber bridging) and physically preventing their propagation [90]. This enhanced stress-transfer ability of the synthetic fibers increased the fracture toughness of the hybrid geopolymer composite, transforming the matrix from brittle to more ductile and capable of load-bearing after fracture [91].

### 3.4. Transport Properties

**Figure 10:**
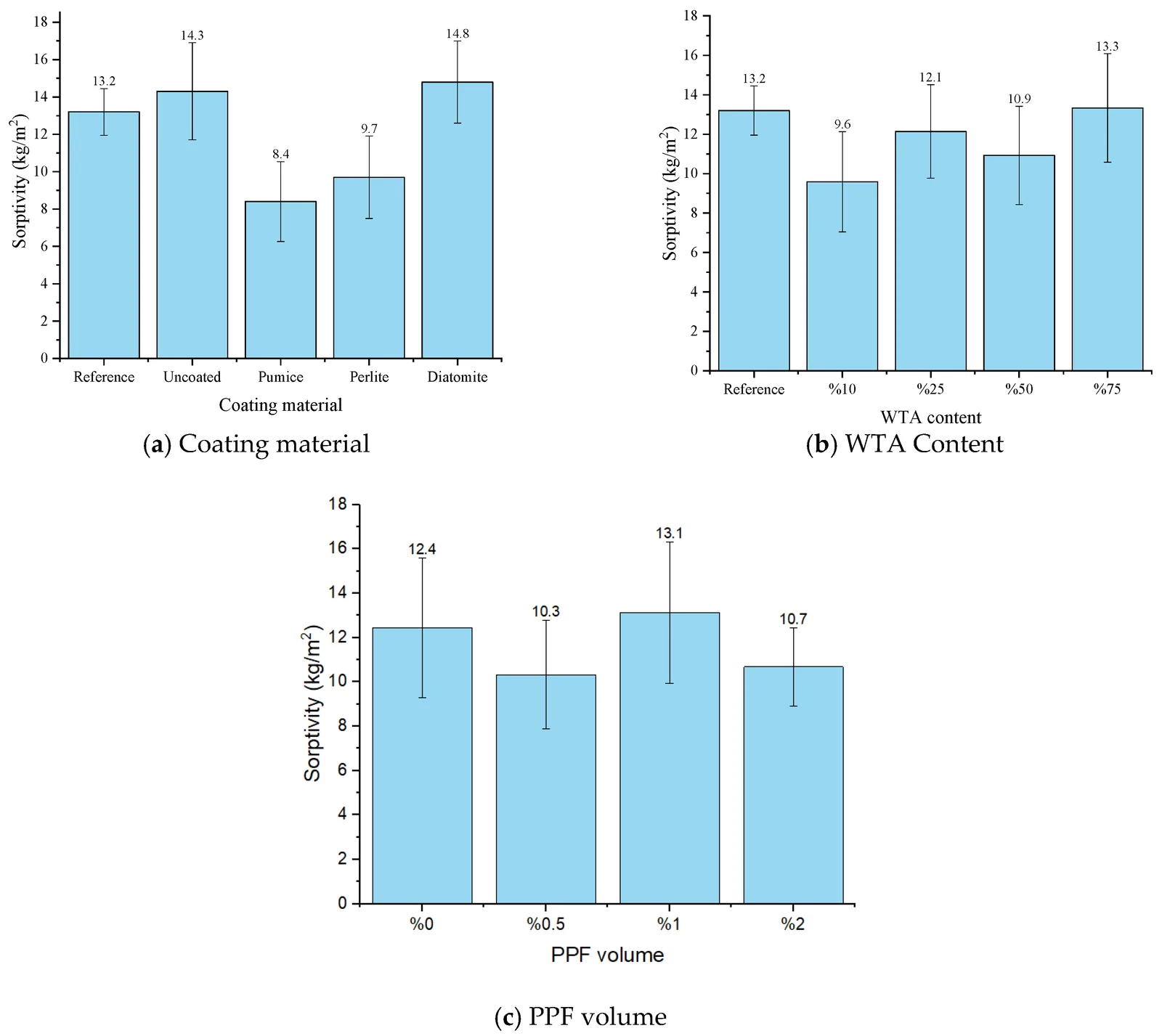
Capillary water absorption of hybrid geopolymer composites.

When examining the capillary water absorption of the developed single-component hybrid geopolymer composites (Figure 10a), it was observed that the continuity of capillary voids (pore connectivity) and the microstructural quality in the interface transition zone (ITZ) showed characteristic variations based on the types of coatings used. Experimental results indicated that the capillary water absorption for the reference mixture was 13.2 kg/m^2^. This value increased by 8.33% to 14.3 kg/m^2^ with uncoated WTA. The rise was attributed to a weak interface between the uncoated rubber particles and the hybrid binder matrix, along with microcracks in this region, which form interconnected pores that facilitate capillary water movement [87,92]. When evaluating the effects of surface modifications, the series coated with pumice and perlite exhibited the highest impermeability. The capillary water absorption value dropped significantly by 36.36%, reaching 8.4 kg/m^2^ in the pumice-coated series and by 26.51%, reaching 9.7 kg/m^2^ in the perlite-coated series, compared to the reference mixture. This improved physical property is directly linked to the fact that these natural pozzolans, rich in amorphous silica, initiate secondary reactions in an alkaline environment, consistent with the formation of additional C-(A)-S-H- and N-A-S-H-type reaction products. Consequently, by blocking capillary voids in the matrix (pore-blocking effect), they chemically densify the interface [93]. The diatomite-coated series showed a 12.12% increase in capillary water absorption compared to the reference mixture, reaching a maximum of 14.8 kg/m^2^. This rise is due to diatomite’s very fine porous structure and high specific surface area, which quickly absorb free water needed for polymerization. This process disrupts matrix density, creating weak phases or voids that are prone to capillary water absorption, consistent with earlier porosity results.

When evaluating the effect of the volumetric substitution rate of waste tire aggregate on the capillary water absorption capacity of the matrix, it was found that two opposing physical mechanisms operated at low and high additive dosages (Figure 10b). The capillary water absorption value, which was 13.2 kg/m^2^ in the reference mixture, significantly decreased by 27.27% to 9.6 kg/m^2^ with a 10% WTA substitution. This decrease was attributed to the hydrophobic (water-repellent) nature of the rubber particles, which reduced capillary tension and prevented water molecules from penetrating the matrix, acting as a physical barrier [94]. Furthermore, the filler effect of small amounts of flexible rubber particles filling the capillary voids in the hybrid matrix also contributed to this impermeability. However, as the WTA substitution rate increased from 10% to 75%, the capillary water absorption value began to rise again, reaching 13.3 kg/m^2^ (a 0.76% increase compared to the reference). The primary reason for losing the hydrophobic surface advantage at high rubber volumes is reduced adhesion at the interface (ITZ) due to increased particle surface area, leading to macro- and microcracks that form new capillary channels for water transport. It was found that increased porosity and disrupted matrix continuity accelerate capillary water movement (capillary suction), weakening the composite’s impermeability.

Among the investigated fiber levels, 0.5% PP fiber produced the lowest capillary water absorption and was therefore the preferred level specifically for this transport response. In contrast, excessive dosages can disrupt this structure. The average capillary water absorption rate, recorded at 12.4 kg/m^2^ in the fiber-free (0% PPF) series, decreased by 16.93% to 10.3 kg/m^2^ when 0.5% PP fiber was included. This improvement is attributed to the synthetic PP fibers forming a three-dimensional reinforcement network within the matrix, which limits microcrack formation caused by autogenous and drying shrinkage during geopolymerization through a fiber-bridging mechanism. Consequently, it physically interrupts the capillary crack network through which water could penetrate [95]. Increasing the fiber usage rate to 1% increased sorptivity to 13.1 kg/m^2^, a 5.64% increase compared to the fiberless series. At 2%, the sorptivity fluctuated at 10.7 kg/m^2^. The observation confirmed this increase in permeability at high PP fiber volumes, as polymeric fibers did not distribute evenly in the fresh mortar. Instead, they agglomerated, reducing workability, hindering matrix compaction, and creating new interconnected capillary air voids within the matrix.

### 3.5. Durability Properties

#### 3.5.1. High Temperature

Since the thermal exposure was applied immediately after the standard 28-day curing period, all residual strengths reported in this section are compared against the 28-day unheated strengths of the corresponding series; the comparisons therefore represent age-matched residual strength ratios, unaffected by continued curing.

**Figure 11:**
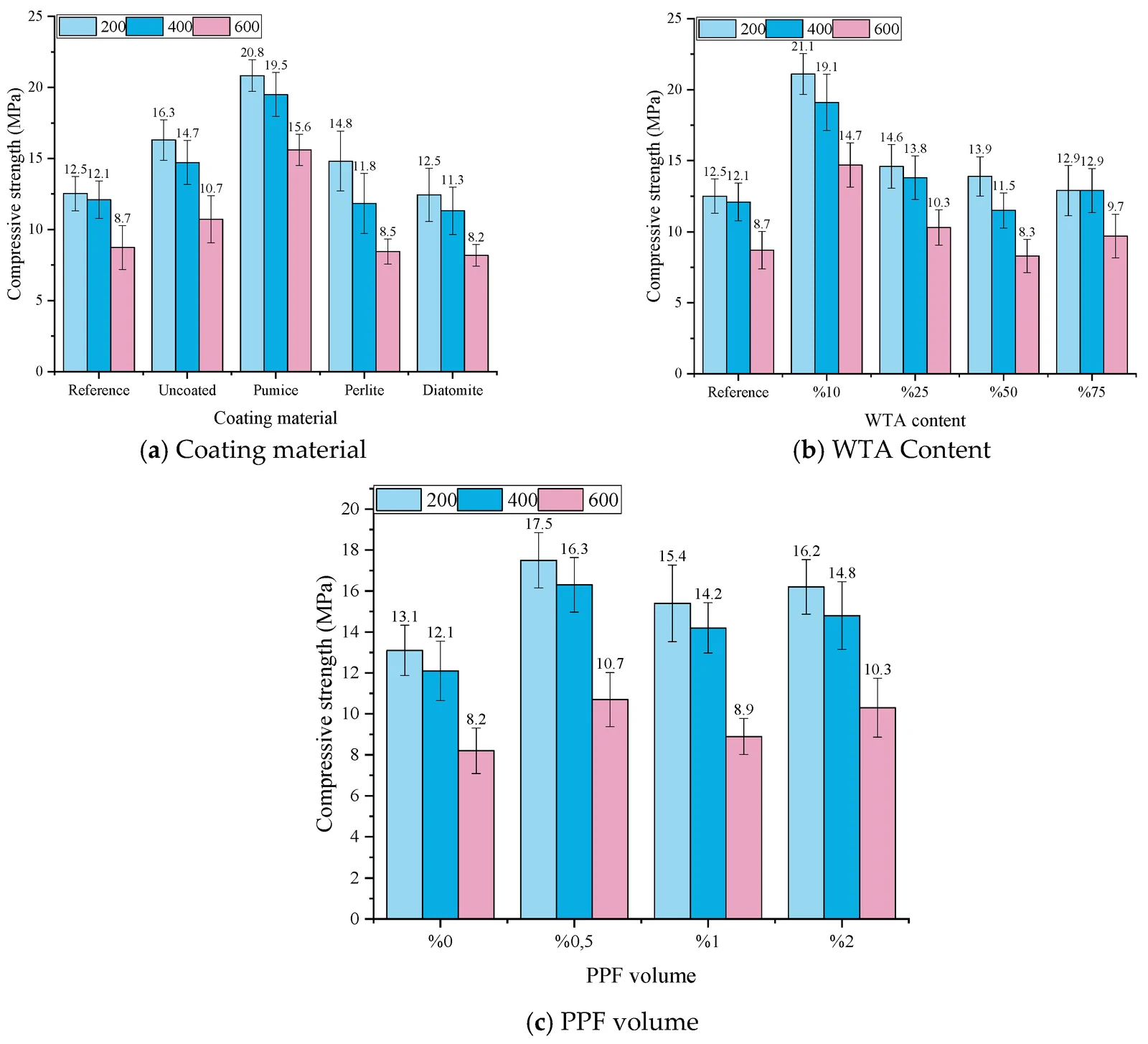
Compressive strength of hybrid geopolymer composites after exposure to high temperatures.

When analyzing the compressive strength data for the developed one-part hybrid geopolymer composites exposed to high temperatures of 200 °C, 400 °C, and 600 °C (Figure 11), characteristic phase transitions indicating the material’s thermal resistance and microstructural stability were observed. While the 28-day compressive strength of the reference mixture—which lacked WTA or PP fibers—was 9.7 MPa, it increased to 12.5 MPa at 200 °C, representing a 28.86% increase. This mechanical improvement, which aligns with similar thermal behaviors reported in the literature, is attributed to relatively low thermal exposure at 200 °C, which induces a secondary thermal curing effect within a single-component system, thereby accelerating the activation of unreacted fly ash and slag particles. At the same time, it was confirmed that the evaporation of free and weakly bound water within the matrix caused the C-(A)-S-H and N-A-S-H hybrid gel structures to become denser, which reduces the capillary voids and strengthens the supporting skeleton [96,97]. When the temperature increased to 400 °C, the strength dropped to 12.1 MPa. At 600 °C, the residual strength of the reference mixture decreased to 8.7 MPa, corresponding to a 10.31% loss relative to its 28-day value (9.7 MPa), due to the loss of chemically bound water and dehydroxylation within the gel structure.

When assessing the high-temperature performance of natural pozzolanic coatings used to address the weak interface zone (ITZ) issue caused by WTA, the pumice-coated series showed the highest thermal stability (Figure 11a). While the 28-day compressive strength in mixtures modified with pumice was 15.5 MPa, it increased by 34.19% at 200 °C and by 25.80% at 400 °C, reaching peak values of 20.8 MPa and 19.5 MPa, respectively. Moreover, even after exposure to a damaging temperature of 600 °C, the residual strength remained at 15.6 MPa, exceeding the initial 28-day strength of 15.5 MPa. The amorphous silica-rich structure and thermal stability of volcanic pumice effectively mitigated thermal incompatibility between the rubber aggregate and the geopolymer matrix, thereby limiting microcracking at the interface. Conversely, while the diatomite and perlite-coated series—known for their high water demand—showed strength increases of 37.36% (12.5 MPa) and 9.62% (14.8 MPa), respectively, at 200 °C compared to their initial strengths at 600 °C, the strength of the diatomite series decreased by 9.89% (to 8.2 MPa), and that of the perlite-coated series dropped by 37.03% (to 8.5 MPa) compared to their initial strengths. It has been determined that this sharp thermal degradation observed in perlite and diatomite modifications is caused by the high amount of water trapped within the material’s internal pores, which generates intense vaporization pressure under heat and physically disrupts the matrix integrity [98].

When analyzing how WTA, incorporated into the system in increasing volumetric proportions, affects thermal resistance, it was found that the characteristic phase transitions of rubber particles at high temperatures directly impact the residual strength of the composite (Figure 11b). In the group containing 10% by volume WTA, the 28-day compressive strength was recorded at 14.3 MPa; after exposure to 200 °C, this value peaked at 21.1 MPa, a notable increase of 47.55%. This remarkable increase in strength is attributed to the rubber particles’ flexibility at low temperatures, which absorbs thermal expansion within the matrix and balances internal stress concentrations. However, when the exposure temperature was raised to 400 °C and especially to 600 °C (beyond the rubber’s ignition and thermal degradation points), the WTA particles underwent advanced thermal decomposition (pyrolysis of the rubber phase, followed by its partial oxidation in the air atmosphere of the furnace), leaving numerous macro-pores and residual voids that weakened the supporting skeleton [99,100]. This interpretation is consistent with the void networks observed in the SEM analysis (Section 3.7.2); however, as no TGA, gas analysis, or residual char quantification was performed, complete volatilization of the rubber phase cannot be claimed. Consequently, it has been determined that the strength of 10.6 MPa at 28 days in series with the highest WTA content (75%) decreased to 9.7 MPa after exposure to 600 °C, indicating an 8.49% loss. Additionally, it has been shown that the new network of voids created by burned rubber particles within the matrix acts as a pressure-relief chamber. It safely releases high internal vapor pressure without causing the material to shatter explosively (explosive spalling). Although the load-bearing capacity declines, this feature helps the material maintain its overall form [101].

When examining the impact of PP fiber content on the high-temperature resistance and explosive crack resistance of composites, it was observed that the melting point of these fibers triggers a key release mechanism in the matrix’s rheology (Figure 11c). In the batch with 0.5% PP fibers, the 28-day strength was 12.7 MPa, rising by 37.79% to 17.5 MPa at 200 °C. However, once temperatures exceed the fibers’ melting point (around 160–170 °C) and reach 400 °C and 600 °C, the compressive strength declines sharply, reaching 10.7 MPa at 600 °C—a 15.74% reduction from the 28-day strength. Likewise, mixtures with 2% PP fibers show a 13.44% decrease (to 10.3 MPa) after exposure to 600 °C. This thermal behavior arises because organic polypropylene fibers melt and vaporize at high temperatures, thereby forming interconnected capillary channels within the hybrid matrix. These channels facilitate the release of excess water vapor pressure from closed pores without causing severe cracking. However, the resulting porosity greatly diminishes the matrix’s effective cross-sectional area and density, thereby weakening the final compressive strength, consistent with thermal degradation models in the existing literature [96].

**Figure 12:**
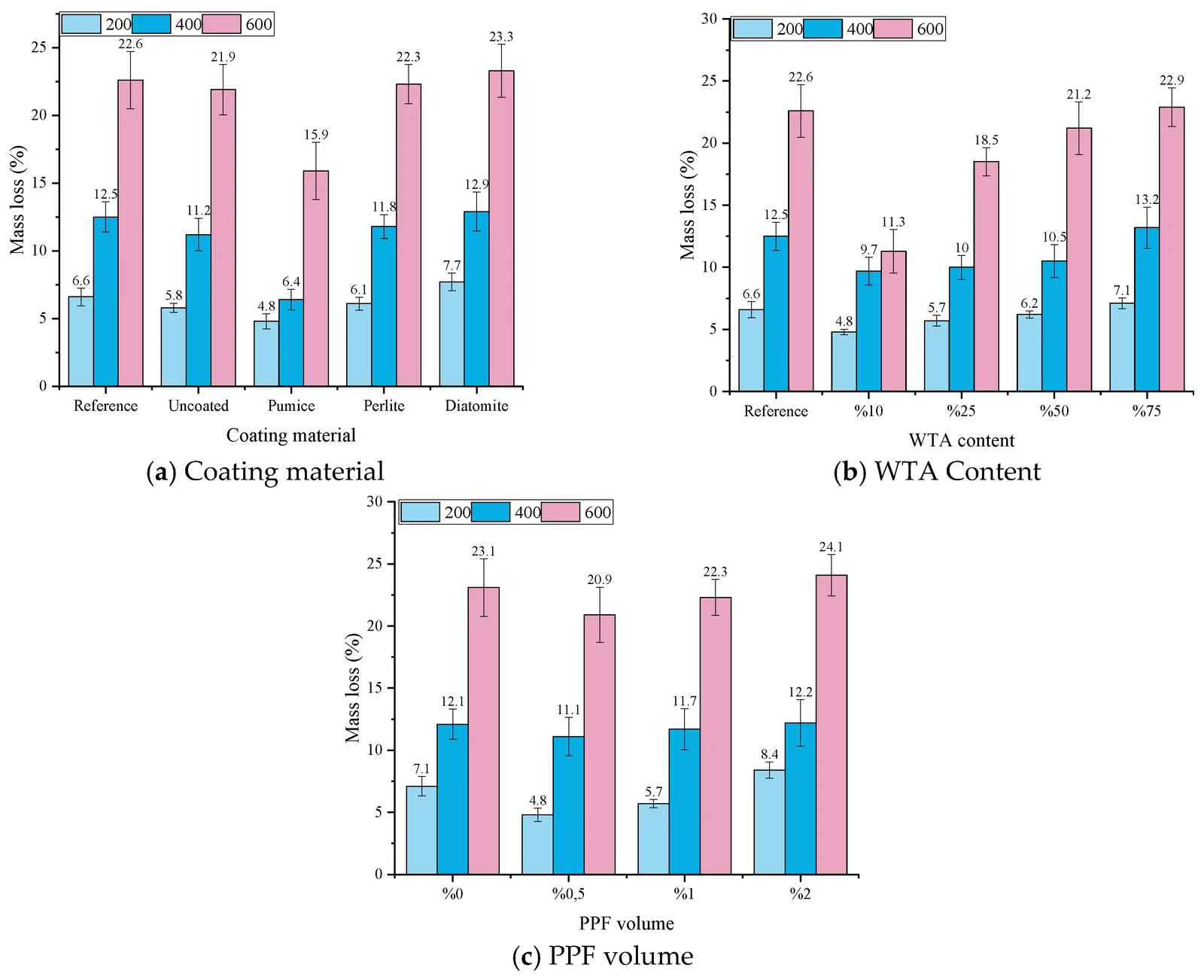
Mass loss of hybrid geopolymer composites after exposure to high temperatures.

When analyzing the mass loss profiles of the developed one-part hybrid geopolymer composites after exposure to high temperatures (200 °C, 400 °C, and 600 °C) (Figure 12), it was found that the material’s thermal degradation kinetics are directly related to increasing temperature and that the matrix typically reflects dehydration stages. In the reference mixture, which served as the baseline for the experimental data, the mass loss at 200 °C was 6.6%; it increased to 12.5% at 400 °C and further to 22.6% at 600 °C. This gradual mass loss is due to the evaporation of free and physically bound water in capillary voids at relatively low temperatures up to 200 °C. At 400 °C and 600 °C, it results from the loss of chemically bound water (hydroxyl groups) in the C-(A)-S-H and N-A-S-H gels—the building blocks of the hybrid matrix—and from the decarbonization of calcium carbonate, as supported by thermogravimetric (TGA) analyses in the relevant literature [102].

When assessing the impact of pozzolanic coating treatments on the WTA surface with respect to mass loss (Figure 12a), the highest thermal stability was observed with pumice modification. The mass loss at 600 °C in the reference mix was 22.6%, but this dropped sharply to 15.9% in the pumice-coated mixes, a decrease of 29.64%. This enhanced thermal resistance stems from pumice’s amorphous silica-rich structure, which promotes alkali reactions, densifies the interface transition zone (ITZ), and physically slows the rapid vaporization and escape of trapped water at high temperatures. Conversely, the diatomite-coated series showed a mass loss of 23.3%, only 3.09% higher than the reference. The organic-based, very fine, porous structure of diatomite traps water during polymerization, which quickly evaporates at high temperatures, resulting in a rapid decrease in the composite’s mass.

When examining how the volumetric WTA replacement ratio affects mass loss (Figure 12b), it was found that changes in rubber volume create two distinct thermal behaviors that oppose each other. Interestingly, in the series with 10% WTA, the mass loss at 600 °C decreased sharply by exactly 50.00% relative to the reference mixture, to 11.3%. It was understood that the hydrophobic surface of rubber particles at low volumes limited the amount of water initially absorbed into the matrix and, as a result, decreased the potential for free water to evaporate during heating. However, when the WTA replacement ratio increased from 10% to 75%, the mass loss at 600 °C increased by 102.65% compared to the 10% series, reaching a peak of 22.9%. The main reason for this high mass loss at high rubber content is that waste tire particles containing styrene-butadiene rubber (SBR) chains exceed their ignition thresholds; since the rubber aggregates decompose (pyrolyze) within the 325–435 °C and 470–525 °C ranges into carbon black and volatile gases, this process leaves behind macro-pores that drastically reduce the matrix’s mass—something clearly shown by previous studies [99,103].

Evaluating the impact of PP fiber content, added to enhance the composite’s toughness, on high-temperature mass loss (see Figure 12c) revealed that an optimal fiber content limits thermal degradation. The mass loss at 600 °C in the fiber-free (0% PPF) group was 23.1%, which decreased by 9.52% to 20.9% with 0.5% PP fiber added. However, increasing the fiber content to 2% resulted in a 4.32% increase in mass loss relative to the fiber-free case, bringing the total to 24.1%. This increase is because organic polypropylene fibers, which melt around 160–170 °C, melt and vaporize at high temperatures such as 600 °C. Higher fiber volumes align with thermal analysis findings, as vaporized polymer fibers reduce the composite’s mass and form capillary channels that facilitate the escape of water vapor [104].

#### 3.5.2. Freeze–Thaw Resistance

**Figure 13:**
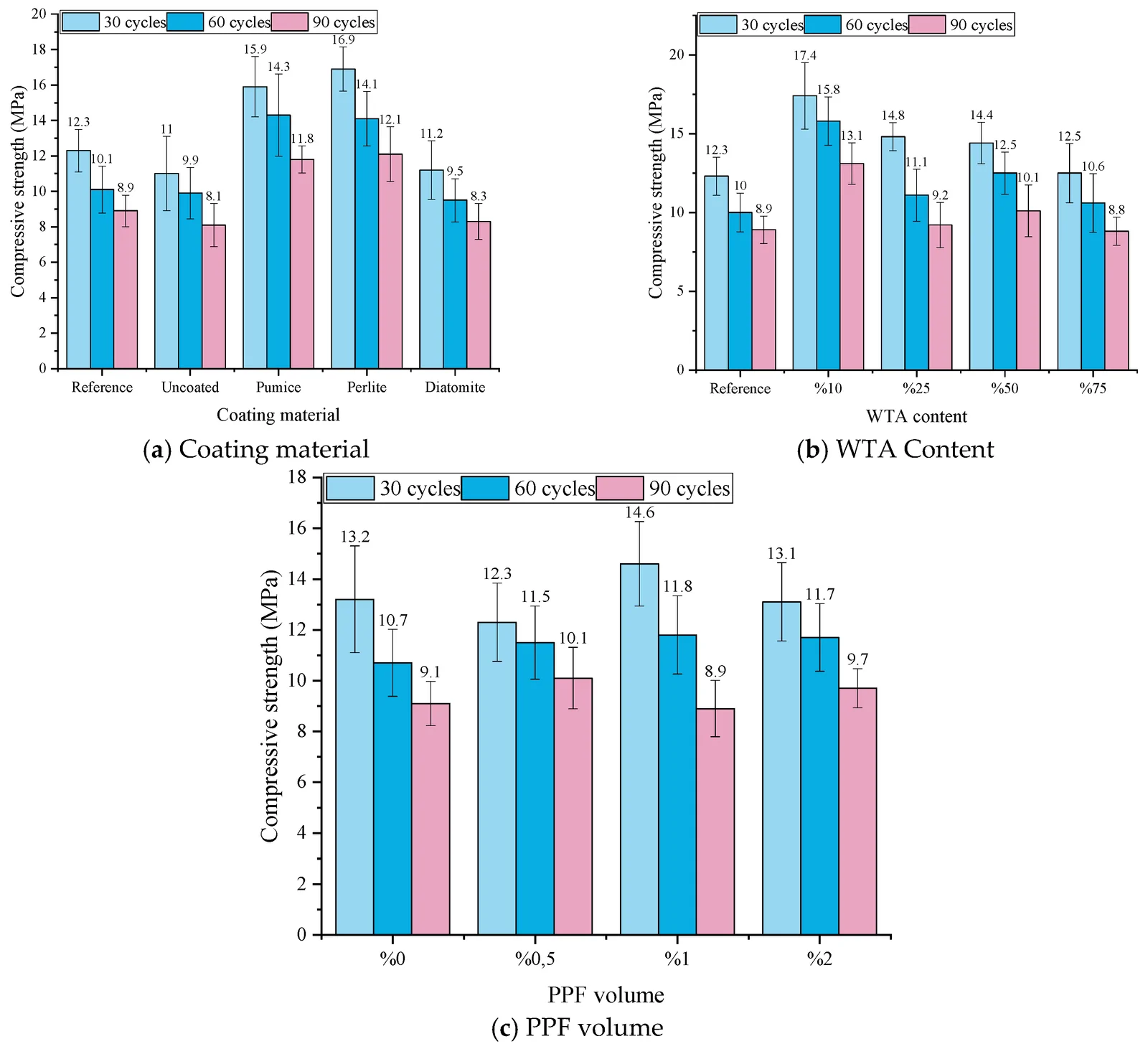
Compressive strength of hybrid geopolymer composites after exposure to freeze and thaw effect.

An analysis of the compressive strength data after freeze–thaw (F-T) cycles, shown in Figure 13a, indicates that the coating type significantly influences the freeze resistance of the hybrid geopolymer matrix. The data show that the strength, which was 12.3 MPa after 30 cycles in the reference mixture, steadily declined with more cycles, reaching 8.9 MPa after 90 cycles—a 27.6% reduction. It was found that uncoated WTA allows capillary water to penetrate due to a weak interface transition zone (ITZ) and microcracks in this area. The expansion pressure from frozen water then accelerates matrix fragmentation, decreasing the strength below the initial value (8.1 MPa after 90 cycles). For surface modifications, the best results were observed in the perlite- and pumice-coated series. After 90 cycles, the compressive strength reached 12.1 MPa for the perlite-coated series and 11.8 MPa for the pumice-coated series, representing increases of 36.0% and 32.6% over the reference mixture.

This significant structural protection results from the chemical densification of the rubber-matrix interface by amorphous silica-rich pozzolanic coatings formed through alkali activation (possibly associated with secondary C-(A)-S-H/N-A-S-H-type products). These coatings block capillary voids that could allow water to penetrate the matrix. Additionally, they create safe “micro-pressure damping chambers” that accommodate the expansion of frozen water within the spongy structures of pumice and perlite [105,106]. Conversely, in the diatomite-coated series (which reached 8.3 MPa after 90 cycles), macro air voids remained unreacted due to high water demand. These trapped voids served as weak points under cyclic ice pressure, hastening the fragmentation of the load-bearing skeleton.

The impact of volumetric WTA replacement ratio on the matrix’s F-T strength was analyzed (see Figure 13b). Results showed that using rubber in controlled amounts enhances the material’s ability to absorb internal freezing pressure. Specifically, the series with 10% WTA by volume achieved a strength of 17.4 MPa after 30 cycles and 13.1 MPa after 90 cycles, the highest among all groups. At this optimal level, the residual strength after 90 cycles was exactly 47.2% higher than the reference value of 8.9 MPa. The rubber particles’ flexible, low-modulus, and hydrophobic properties function similarly to “air-entraining agents,” trapping micro-air voids within the capillary system. These voids and the elastic rubber phase effectively absorb internal hydrostatic expansion stresses caused by setting water, consistent with scientific data [107,108]. Conversely, increasing WTA replacement to extreme levels, such as 75%, resulted in decreased strength at 90 cycles, dropping to 8.8 MPa, close to the reference. This strength reduction at high replacement ratios is mainly due to excessive constriction of the load-bearing solid skeleton by increased rubber volume and the formation of interconnected channels at the interface, which facilitate freeze–thaw damage through debonding.

When the effect of PP fiber integration into the developed hybrid composites on freeze–thaw-induced structural degradation and internal crack propagation was evaluated (Figure 13c), it was determined that the fibers acted as a mechanical barrier against cyclic ice pressure. The residual strength, recorded at 9.1 MPa after 90 cycles in the fiberless (0% PPF) series, increased to 10.1 MPa, representing an 11.0% improvement upon integrating 0.5% PP fibers into the mixture. This observed structural strength was attributed to the three-dimensional network formed by the high-tensile-capacity synthetic fibers within the matrix, which successfully bridged the two sides of the microcracks that begin to open as a result of the lateral expansion forces created by freezing water, physically stopping the growth of the crack opening [109]. However, it has been found that increasing fiber usage to 1% and 2% causes a tendency for the F-T resistance to weaken; for example, although adding 1% PP fiber results in a high early strength of 14.6 MPa after 30 cycles, the residual strength drops sharply to 8.9 MPa after 90 cycles. The agglomeration of high-volume organic fibers in the fresh geopolymer mortar, due to uneven distribution and the resulting damage from freeze–thaw cycles—caused by allowing extra water into the matrix through weak interface regions—has been identified as the microstructural cause of this sudden loss of strength.

**Figure 14:**
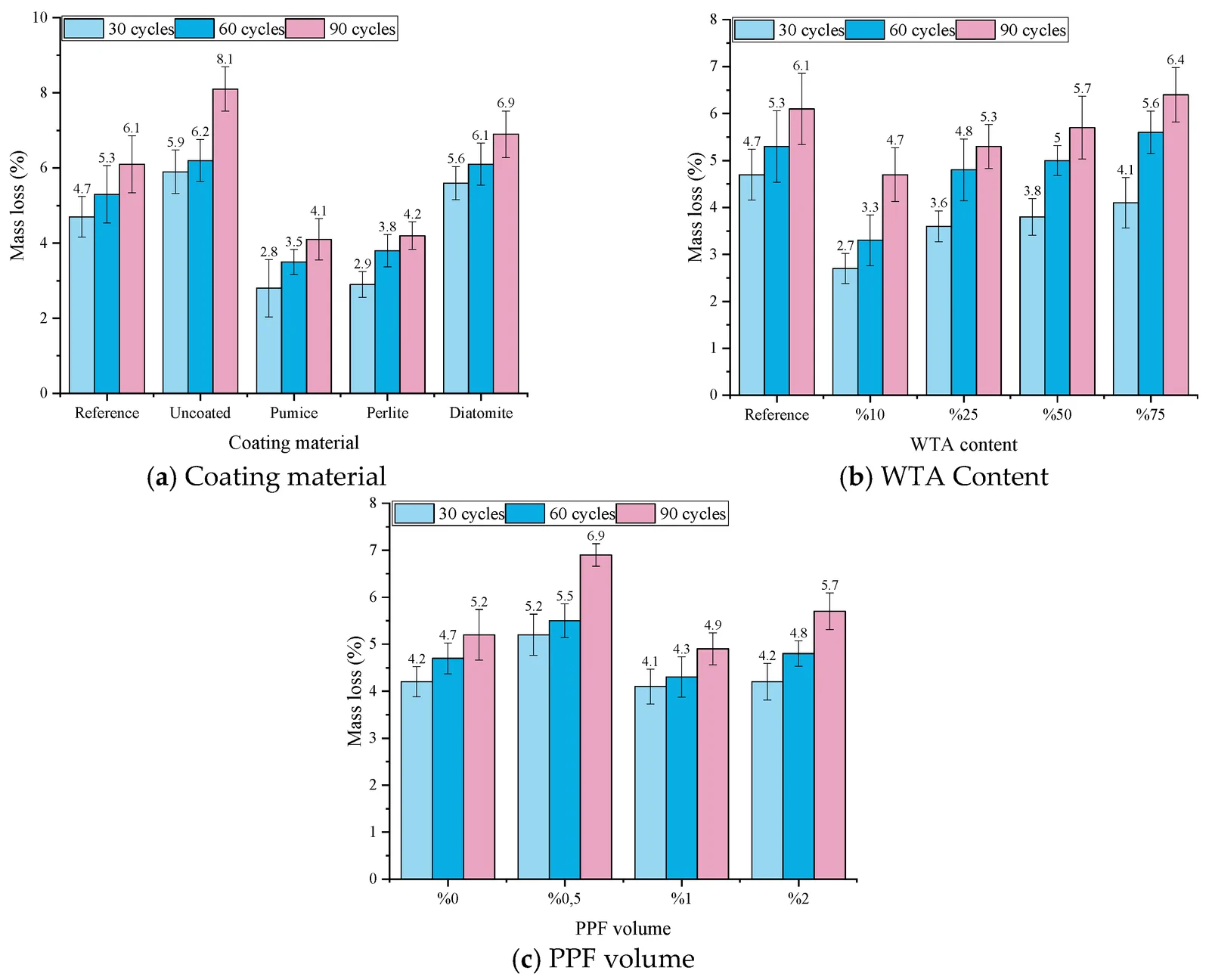
Mass loss of hybrid geopolymer composites after exposure to freeze and thaw effect.

When examining the mass-loss (weight-loss) data after freeze–thaw (F-T) cycles shown in Figure 14a, it is evident that surface degradation (spalling) and the matrix’s inherent disintegration resistance exhibit distinct characteristics depending on the type of pozzolanic coating used. While the reference mixture’s mass loss after 90 cycles was 6.1%, it increased significantly to 8.1% in the series containing uncoated WTA. This serious physical damage is explained by the hydrophobic nature of the uncoated rubber, which creates a weak bond at the interface (ITZ), and by the expansion pressure caused by the freezing of capillary water infiltrating these areas, which leads to scaling on the matrix surface [106]. When evaluating the effects of surface modifications, the series coated with pumice and perlite provides the best protection. After ninety cycles, mass loss in pumice-coated mixtures decreased by 32.8% to 4.1% compared to the reference, while in perlite-coated mixtures, it decreased by 31.1% to 4.2%. This high physical strength of pumice and perlite is due to amorphous silica-rich pozzolans chemically repairing the interface through alkali activation, improving the pore structure, and reducing the internal expansion stresses caused by freezing water. In contrast, the mass loss in the diatomite-coated series after 90 cycles reached 6.9%, which is a 13.1% increase over the reference. This is attributed to diatomite’s very high water absorption capacity, which accelerates physical disintegration by carrying a greater ice load within the matrix during freeze–thaw cycles.

When examining how the volumetric waste tire aggregate substitution ratio affects the mass damping behavior under F-T cycles (Figure 14b), it was found that low rubber additive levels optimize the composite’s physical resistance to internal freezing pressure. After 90 cycles, the mass loss in the reference mixture, which was 6.1%, was successfully reduced to 4.7% with a 10% WTA substitution, reflecting a 23.0% decrease. This specific improvement was confirmed by the observation that the flexible rubber particles, acting as air-entraining agents in the fresh mortar, trap a controlled number of micro-air voids. These voids safely dampen the hydrostatic pressure from freezing water without causing spalling on the outer surface [61,107]. It was determined that gradually increasing the WTA substitution ratio to an extreme 75% results in a renewed increase in mass loss to 6.4%, which is 4.9% higher than the reference. The main cause of increased freeze–thaw damage in high-volume rubber is the severe weakening of the binder matrix skeleton, the formation of continuous channels allowing water infiltration due to debonding at the rubber-matrix interface, and ultimately, the deterioration of the material’s physical integrity under ice expansion [61].

When evaluating the impact of PP fiber addition in the developed hybrid composites on F-T-induced mass loss and surface abrasion (Figure 14c), it was found that fiber addition, up to a specific optimal ratio, limited damage by maintaining matrix integrity. However, surpassing this threshold triggered degradation reactions. The average mass loss, which was 5.2% after 90 cycles in fiberless (0% PPF) mixtures, decreased by 5.8% to 4.9% with the inclusion of 1% PP fiber, resulting in the lowest damage rate in this group. This protective effect of synthetic fibers aligns with findings in the literature, as it results from preventing the propagation of internal microcracks caused by freezing pressure (fiber bridging). This is achieved by establishing a three-dimensional reinforcement network within the matrix that physically holds together hairline fractures (scaling) on the outer surface [109]. Conversely, increasing the fiber content to 2% led to a 9.6% increase in mass loss compared to the fiber-free mix, bringing the total to 5.7%. The microstructural reasons for this include the agglomeration of large amounts of PP fibers within the fresh geopolymer pulp, which makes placement difficult and hampers effective compaction. Additionally, physical disintegration accelerates under rising ice pressure due to trapped weak air voids (macro-pores) within the structure [110].

#### 3.5.3. MgSO_4_ Effect

**Figure 15:**
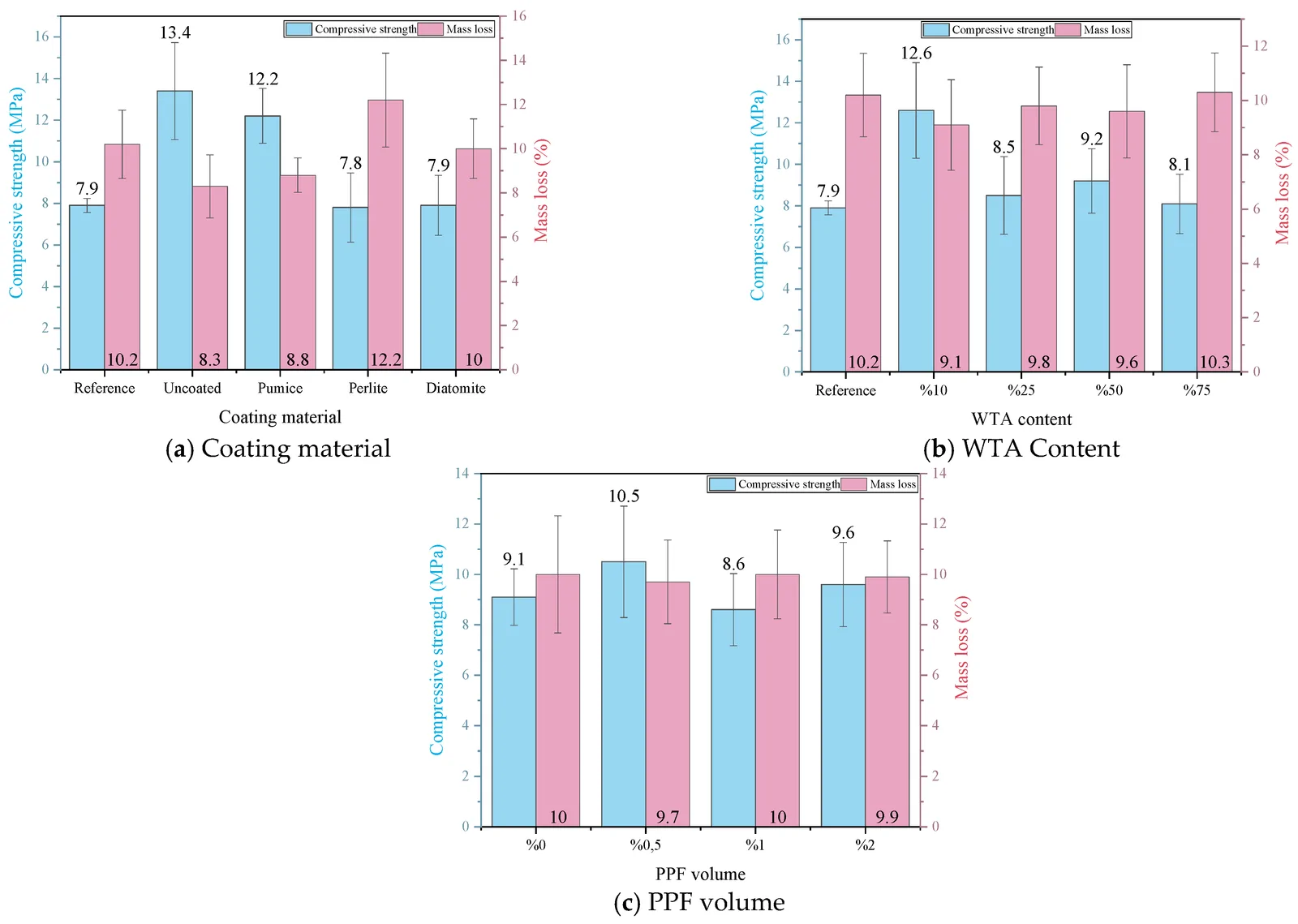
MgSO_4_ exposure and change in hybrid geopolymer composites.

When the compressive strength and mass loss data obtained by exposing the developed one-part hybrid geopolymer composites to a 5% MgSO_4_ solution for 120 days, following a standard 28-day curing process (Figure 15a), were examined, it was found that aggressive sulfate ions directly accelerate chemical degradation in the matrix. The reference mixture, which initially had a compressive strength of 9.7 MPa, decreased to 7.9 MPa (18.55% loss) after sulfate exposure and also experienced a mass loss of 10.2%. The main reason for this structural weakening is the reaction of calcium-rich C-(A)-S-H gels from fly ash and slag with magnesium ions, leading to decalcification and the formation of M-S-H (magnesium silicate hydrate) gels that lack binding ability and tend to expand volumetrically [99]. Contrary to common beliefs in the literature, a significant increase in performance was observed in series using uncoated WTA. In this series, the compressive strength increased by 35.35%, from 9.9 MPa to 13.4 MPa, while the mass loss remained the lowest among all tested coated groups at 8.3%. This remarkable improvement is attributed to the uncoated rubber’s strong hydrophobic (water-repellent) properties, which serve as a physical barrier, preventing the sulfate solution from penetrating the matrix’s capillary pores. Additionally, it was confirmed that the weak interface (ITZ) voids around the rubber particles act as expansion chambers for gypsum and ettringite crystals formed by the limited solution infiltrating the matrix. These crystals fill the voids, making the system more compact (due to Ostwald maturation/pore-blocking effects) and thereby increasing strength without internal cracks [111]. When pozzolanic coatings were examined, the strength decreased sharply in the pumice-coated series from 15.5 MPa to 12.2 MPa (a 21.29% loss) and in the perlite-coated series from 13.5 MPa to 7.8 MPa (a 42.22% loss). It was determined that the initial chemical densification of the interface by the pozzolanic coatings narrowed the void space where the expanding crystals formed from magnesium sulfate reactions could be quenched; consequently, the increased crystallization pressure directly fractured the carrier matrix from within, accelerating mass and strength losses.

When assessing how volumetric waste tire aggregate substitution ratios affect magnesium sulfate resistance and structural integrity (see Figure 15b), it was found that lower rubber volume ratios enhance the matrix’s resistance to chemical damage. The mixture with 10% WTA by volume exhibited a 28-day compressive strength of 14.3 MPa. After sulfate exposure, this strength slightly declined by 11.88% to 12.6 MPa, with a mass loss of 9.1%. The high damping capacity at low rubber volumes is attributed to the flexible tire particles acting as micro-buffers that effectively absorb hydrostatic expansion stresses from MgSO4 reactions and preserve the matrix’s overall continuity [112]. However, increasing the WTA substitution rate from 10% to 75% decreased the compressive strength from 10.6 MPa to 8.1 MPa (a 23.58% decrease relative to the reference) and increased the mass loss to 10.3%. The excessive thinning of the matrix skeleton and the increase in weak interfacial regions, along with the larger rubber volume, formed interconnected corrosion pathways that facilitated the diffusion of the sulfate solution into the material’s core, leading to increased decalcification and dissolution and causing macro-level degradation of the material.

When the effects of the volume of PP fibers integrated into the system on the toughness losses and mass degradation of composites under magnesium sulfate corrosion were investigated (Figure 15c), it was determined that the optimum fiber dosage physically limited sulfate damage. In the fiber-free (0% PPF) series, the 28-day compressive strength was 11.7 MPa, which decreased by 22.22% to 9.1 MPa after 120 days of sulfate attack. When 0.5% PP fiber was added to the system, the residual strength was measured at 10.5 MPa, corresponding to a 17.32% strength loss and a 9.7% mass loss. The literature findings are in complete agreement with the observation that, at optimal levels, synthetic fibers form a homogeneous network within the matrix, bridging the ends of microcracks caused by sulfate crystallization pressure and physically preventing the penetration of sulfate ions through the capillary crack network (crack propagation) [43]. However, increasing the PP fiber dosage to 1% and 2% had the opposite effect on sulfate corrosion resistance, resulting in strength losses of 33.33% (to 8.6 MPa) and 19.32% (to 9.6 MPa), respectively. It was determined that the agglomeration of high-volume organic fibers in fresh mortar, which impairs workability and prevents effective compaction, creates additional air voids within the matrix, allowing sulfate-bearing water to penetrate more rapidly and to greater depths, thereby accelerating the matrix’s internal disintegration.

Examining Figure 15a shows that the mass loss, which was 10.2% after 120 days in the reference mixture, decreased to 8.3% in the series with uncoated WTA, which offered the best preservation. This is because the uncoated rubber’s strong hydrophobicity (water-repellency) limits the sulfate solution’s penetration into the capillary pores, thereby acting as a physical barrier [113]. Examining the effect of pozzolanic coatings, the mass loss was 8.8% in the pumice-coated series and 12.2% in the perlite-coated series. The surface modifications initially densified the interface (ITZ), reducing voids and leaving insufficient space to accommodate the internal crystallization pressure generated by expanding crystals from magnesium sulfate reactions. This led to greater internal disintegration of the perlite-coated matrix, accelerating surface decomposition and mass loss [114].

When evaluating the impact of volumetric WTA substitution ratio on mass change (Figure 15b), it was found that lower rubber volume ratios enhance the matrix’s mass stability. The reference mixture’s 10.2% mass loss reduced to 9.1% with 10% WTA substitution. Small rubber particles, characterized by a low elastic modulus and flexibility, served as micro-buffers, absorbing hydrostatic expansion stresses induced by sulfate crystallization and preventing the material from disintegrating and flaking at larger scales [113]. However, when the WTA substitution ratio reached 75%, the mass loss rose again to 10.3%. The weakening of the matrix’s supporting structure and the reduced adhesion at the rubber-matrix interface with higher rubber content led to highly porous corrosion pathways. These pathways allowed the sulfate solution to diffuse readily into the composite’s core, accelerating the natural degradation and mass loss of the matrix [113].

When examining how the amount of PP fibers in the system affects mass degradation under MgSO4 corrosion (see Figure 15c), it was found that optimal fiber usage can partially reduce sulfate damage. The mass loss was 10.0% in mixtures without fibers (0% PPF), but it decreased slightly to 9.7% with the addition of 0.5% PP fibers. The formation of a three-dimensional network by randomly distributed synthetic fibers halted microcrack propagation caused by sulfate crystallization through a bridging mechanism, preventing fragments from detaching from the surface. However, increasing the PP fiber content to 1% and 2% did not improve protection, with mass losses of 10.0% and 9.9%, respectively. Excessive fiber volume led to agglomeration in the fresh mortar, creating weak air voids that facilitated faster, deeper sulfate penetration, ultimately reducing the material’s resistance to mass loss [43].

#### 3.5.4. Acid (H_2_SO_4_) Effect

**Figure 16:**
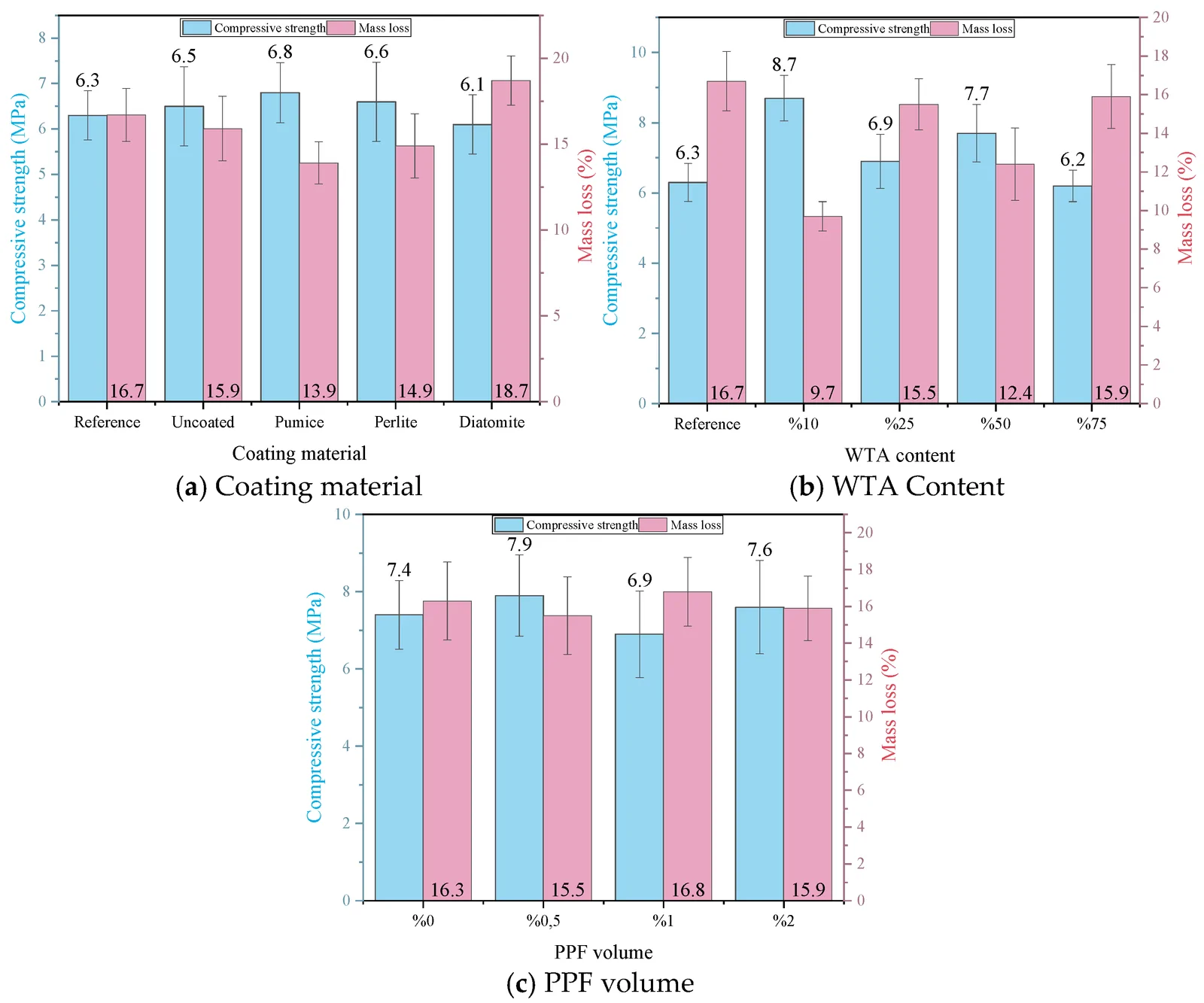
H_2_SO_4_ exposure and change in hybrid geopolymer composites.

The compressive strength and mass (weight) loss data obtained after exposing the developed one-part hybrid geopolymer composites to a 5% H_2_SO_4_ solution for 120 days (Figure 16) clearly demonstrate the dissolving and destructive effects of the acidic environment on the matrix. The primary corrosion mechanism of sulfuric acid attack involves H+ ions attacking the Si-O-Al bonds in the geopolymer network, thereby depolymerizing the aluminosilicate structure. At the same time, SO4 ions react with calcium in the matrix to form gypsum crystals that expand volumetrically. This internal expansion creates stresses that cause layers to spall from the matrix’s outer surface, leading to significant mass loss and weakening of the supporting skeleton, thereby reducing compressive strength [115].

In Figure 16a, the compressive strength of the reference mixture exposed to H_2_SO_4_ was measured at 6.3 MPa, with a mass loss of 16.7%. In the series where WTA was used without coating, the strength increased slightly to 6.5 MPa, while the mass loss decreased to 15.9%. The strong hydrophobicity of the uncoated rubber reduces mass loss by limiting penetration of the sulfuric acid solution into the matrix’s deeper regions, thereby serving as a physical barrier [116]. Surface modification evaluation revealed that pumice-coated samples exhibited the best acid resistance. Specifically, in pumice-coated mixes, the compressive strength increased by 7.9% to 6.8 MPa, and mass loss decreased by 16.7% to 13.9%. This is because pumice’s high amorphous silica content promotes the formation of secondary N-A-S-H and C-(A)-S-H gels in alkaline conditions, sealing the interface (ITZ) and capillary pores (pore-blocking), thereby preventing acid-ion infiltration. Conversely, in diatomite-coated samples, the compressive strength dropped to 6.1 MPa, and the mass loss rose to 18.7%, exceeding that of the reference. Diatomite’s high porosity and water demand hasten dissolution and exfoliation by forming channels that facilitate acid diffusion into the matrix core.

When examining the behavior of the WTA substitution ratio under H_2_SO_4_ corrosion (Figure 16b), it is clear that using low rubber ratios maximizes both mass conservation and strength. Compared to the reference mixture, the 10% WTA mixture achieves a compressive strength of 8.7 MPa, a 38.1% increase, while mass loss drops by 41.9% to 9.7%. Scientific research shows that low-volume hydrophobic rubber particles act as acid-repelling microbuffers within the matrix, slowing the penetration of H+ and SO_4_ ions. They also create internal pressure-damping chambers for sulfate-induced expanding gypsum crystals, preventing macro-level cracking and fragmentation [116]. Increasing the WTA substitution ratio to an extreme level of 75% results in a decrease in strength to 6.2 MPa and an increase in mass loss to 15.9%. Excessive rubber content weakens the matrix binder skeleton, and enhanced interfacial incompatibilities, along with the porous structure, allow the acid solution to contact larger surface areas, thereby accelerating aluminosilicate depolymerization.

When evaluating the effect of PP fiber integration on mass loss and structural degradation during sulfuric acid corrosion (Figure 16c), the optimal fiber dosage was found to maintain physical integrity. In the fiber-free series (0% PPF), the strength after acid exposure was 7.4 MPa, with a mass loss of 16.3%. Adding 0.5% PP fiber to the mixture increased compressive strength by 6.7–7.9 MPa and decreased mass loss to 15.5%. It was observed that synthetic PP fibers, by forming a uniform skeletal network within the matrix, bridged microcracks caused by acid reactions and gypsum expansion (fiber bridging), thereby physically holding together large pieces spalling from the outer surface and thus limiting mass loss [43]. Increasing the PP fiber ratio to 1% reduced the strength to 6.9 MPa and increased the mass loss to 16.8%. The accumulation of excessive amounts of synthetic fibers in fresh mortar produced weak air voids, allowing capillary absorption of an H_2_SO_4_ solution and rendering the fibers’ protective effect against corrosion ineffective.

### 3.6. Statistical Analysis and Response-Specific Taguchi Assessment

It should be emphasized that the Taguchi L16 orthogonal array with three four-level factors does not allow the resolution of two-way or higher-order interaction terms, which are pooled into the error term of the ANOVA. Consequently, all statistical interpretations and optimization conclusions in this study are restricted to main-effect trends, and no claims of synergistic or interaction effects between factors are made.

The Taguchi assessment was conducted separately for each response, without using a formal multi-criteria decision-making model. The 28-day compressive strength and flexural toughness were evaluated individually with the larger-the-better S/N criterion. In contrast, apparent porosity, capillary water absorption, and oven-dry density were assessed using the smaller-the-better criterion. No normalization, response weighting, composite objective function, grey relational grade, desirability index, or overall multi-response ranking was performed. Consequently, the results in this section should be viewed as response-specific trend analyses rather than as a formal multi-objective optimization. The optimal material configuration was chosen based on an engineering comparison of the independent response trends, explicitly considering the trade-offs among strength, toughness, compactness, and lightweight properties.

**Figure 17:**
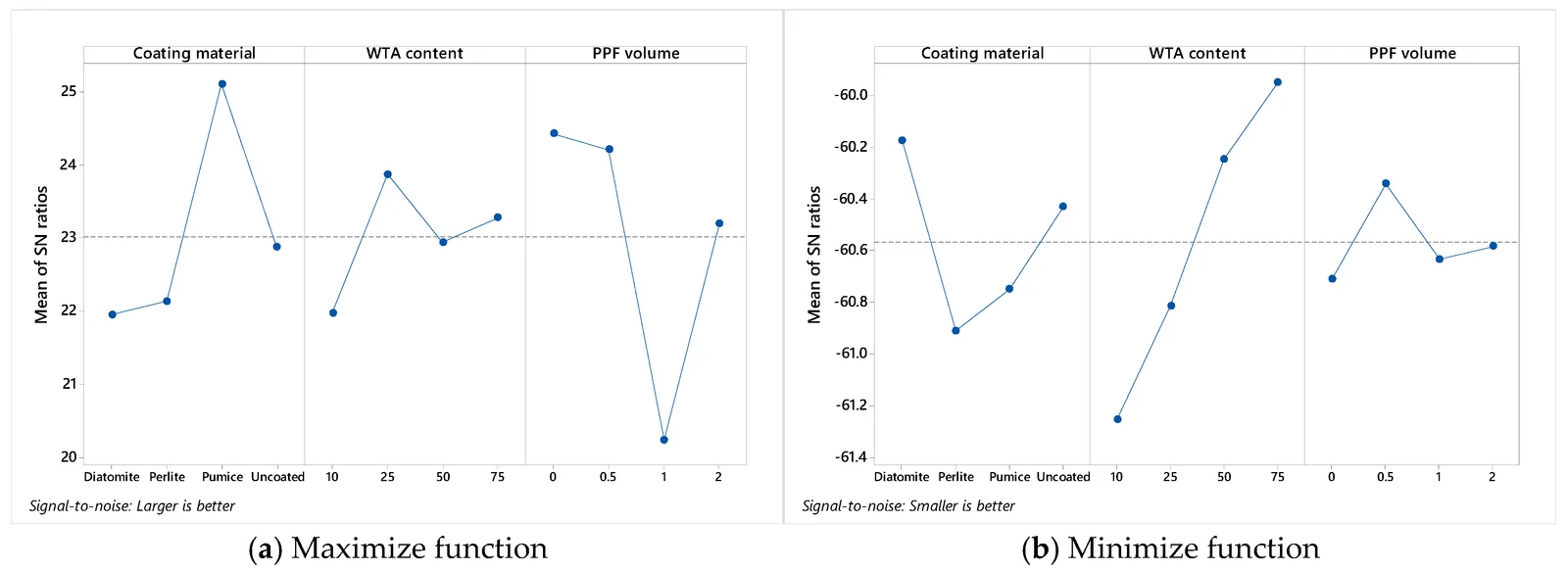
S/N ratios according to Taguchi optimization.

A response-specific Taguchi assessment process for optimizing the developed one-part hybrid geopolymer composites, 28-day compressive strength and flexural toughness were chosen as the key mechanical responses to be maximized using a “larger-is-better” strategy (Figure 17a). Including both parameters in the objective function reflects the need to balance their often inversely related and competing roles—namely, the material’s load-bearing capacity versus its ductility and energy dissipation ability.

Compressive strength is considered the most important engineering indicator, reflecting the integrity of the matrix, the capacity of the load-bearing frame, and the overall structural quality of a building material. The low elastic modulus and strong hydrophobic surface properties of WTA, when integrated into the system, weakened the interface transition zone (ITZ) between the binder geopolymer and the rubber particles, thereby decreasing the composite’s compressive strength. Therefore, to ensure that the lightweight composite system can be used as a reliable material for non-structural and semi-structural applications, the goal was to overcome this strength reduction caused by WTA and micro-voids through pozzolanic coatings, thereby maximizing compressive strength within structural limits. On the other hand, the primary scientific motivation for incorporating WTA and PP fibers into a geopolymer matrix, which is inherently very brittle, is to enhance the material’s fracture energy and toughness. It has been demonstrated that rubber particles, due to their flexible structure, act as microbuffers by damping internal stresses during deformation, and that synthetic PP fibers, by creating a three-dimensional, random skeletal network within the matrix, physically hinder the growth of microcracks under tensile stresses through a fiber-bridging mechanism. Since flexural toughness measures the total energy the material absorbs from the first crack to failure (post-cracking behavior), it was selected as the parameter in the optimization model, allowing the increased ductility provided by PP fibers and WTA to be assessed most directly and clearly. In summary, while high volumes of WTA and PP fibers enhance the composite’s flexural toughness and ductility, they can also reduce compressive strength due to interfacial issues and the risk of agglomeration. Due to this fundamental trade-off, both factors were incorporated into the optimization process, enabling a precise identification of the optimal coating type, rubber volume, and fiber dosage. This combination ensures both adequate mechanical integrity for the targeted non-structural applications and improved post-cracking energy absorption.

Analyzing the Taguchi Signal-to-Noise (S/N) ratio graph, which evaluated 28-day compressive strength and flexural toughness using a “larger-is-better” criterion, revealed the optimal levels of independent variables that simultaneously enhanced both load capacity and ductility of the matrix. The highest S/N score for the coating type was achieved with pumice coating. This is because pumice, rich in amorphous silica and with high pozzolanic reactivity, possibly contributes to the chemical repair of the ITZ between rubber particles and the geopolymer paste through the formation of C-(A)-S-H and N-A-S-H gels during alkali activation, thereby reinforcing the load-bearing structure against compression and improving mechanical interlocking under bending. When evaluating the optimization trend of the WTA substitution ratio, it was found that the S/N ratio peaked at 25% usage, then declined sharply as rubber volume increased beyond this optimal level. It was confirmed that only 25% of hydrophobic, low-elastic-modulus rubber particles provided the ideal contribution by acting as micro-buffers to improve flexural toughness (energy absorption capacity after cracking) without compromising the overall compactness and structural integrity of the matrix. The fact that high WTA volumes increased adhesion weakness and reduced the effective carrier solid cross-section, thereby decreasing mechanical capacity to unacceptable levels, was identified as the primary microstructural reason for this sharp, unavoidable drop in the S/N ratio. When examining the behavior of PP fiber content in composite optimization, it was found that the optimal level (fiber-free) produced the highest peak S/N value, effectively balancing the trade-off between compressive strength and flexural toughness.

Analyzing the Taguchi “smaller-is-better” optimization model, which aims to reduce apparent porosity, capillary water absorption, and hardened unit weight simultaneously, we identified structural limits that maximize impermeability without losing the matrix’s lightweight benefit (Figure 17b). When evaluating the coating type as an independent variable, the highest S/N optimization peak appeared in the pumice-coated series (and similar silica-rich pozzolanic modifications). This high performance is attributed to pumice triggering secondary pozzolanic reactions in an alkaline environment, consistent with the formation of additional C-(A)-S-H- and N-A-S-H-type reaction products that physically block the weak interfacial transition zone (ITZ) around rubber particles (pore-blocking effect). This process helps maintain the composite’s lightweight targets while effectively minimizing capillary water pathways that carry harmful substances from the environment, as well as unnecessary air voids.

When the Taguchi “smaller-is-better” Signal-to-Noise (S/N) optimization, which aims to simultaneously minimize apparent porosity, capillary water absorption, and hardened unit volume weight, is evaluated holistically, it is found that each independent variable integrated into the system influences the material’s physical characteristics through specific mechanisms. According to the optimization results, using WTA, which is significantly lighter than conventional silica sand, at a maximum rate of 75%, coupled with choosing a diatomite coating of biogenic origin with a spongy microstructure and high specific surface area, dramatically lowered the overall density of the material and maximized the system’s S/N score. Although the increased rubber volume and the high water demand of diatomite tend to slightly increase capillary porosity by causing interface (ITZ) weaknesses, it was determined that this lightness advantage was mathematically dominant in the Taguchi minimization algorithm and fully achieved the ultimate goal of lightening. On the other hand, when the effect of PP fiber content on physical minimization was examined, it was confirmed that a low and controlled fiber dosage of 0.5% successfully bridged microcracks caused by shrinkage, effectively blocking capillary water pathways and reducing the matrix’s water absorption tendency; however, increasing the dosage to 1% and 2% led to organic fibers agglomerating in the fresh mortar, disrupting effective compaction, and directly lowering the optimization score due to trapped macrovoids in the structure. In conclusion, it has been scientifically proven that the Taguchi algorithm, applied most effectively, identifies the optimal material configuration that balances maintaining the composite’s structural impermeability with achieving bulk reduction (low density).

When analyzing the results of the Analysis of Variance (ANOVA) for the 28-day compressive strength and flexural toughness data of one-part hybrid geopolymer composites produced according to the Taguchi L16 experimental design matrix, the statistical importance of the independent variables on load-bearing capacity and energy absorption performance was clearly identified (Table 6). For the compressive strength results, the most influential and statistically significant factor affecting load-bearing capacity was the type of surface coating, which accounted for an overwhelming 76% of the variance. 2% and a *p*-value of 0. 0.002 (*p* < 0. 05). The high statistical effect is consistent with enhanced formation of C-(A)-S-H- and N-A-S-H-type reaction products around the WTA, created by amorphous silica- rich pozzolanic coatings around the WTA, in chemically repairing the weak interfacial transition zone (ITZ). Additionally, the WTA substitution rate was also statistically significant for compressive strength, contributing 20.9. 9% with a *p*-value of 0. 045. This data confirms the direct impact of flexible rubber, with a much lower modulus of elasticity than traditional silica sand, on stress-transfer weaknesses within the matrix. Conversely, the contribution of PP fiber volume to compressive strength was limited to only 2. 2.9% and was not statistically significant (*p* = 0. 592); this aligns with the understanding that the primary mechanical function of synthetic fibers is not load- bearing under compression but enhancing post- cracking toughness under tension and bending. When examining the variance in bending toughness, the highest effect was attributed to PP fiber volume, with 48–2%. The three- dimensional network formed by synthetic fibers within the matrix improves ductility through fiber bridging. Although coating type contributed 31.2. 2% and WTA ratio 20. 6% to bending toughness, the *p*-values exceed 0.05, indicating that energy dissipation at fracture cannot be attributed to any single factor at a statistically significant level within the resolution of the L16 array.

The ANOVA analysis of oven-dry density, apparent porosity, and capillary water absorption, which reflect the composite’s physical stability, revealed that different independent variables control the bulk reduction and matrix compactness objectives. For density minimization—a physical goal—the WTA substitution ratio, with an overwhelmingly dominant additive ratio of 73.6%, was the primary factor affecting the bulk and approached statistical significance (*p* = 0.058). Increasing the volume of polymeric rubber aggregates, which have a much lower specific gravity than traditional minerals, directly reduces the system’s structure, confirming it as the main contributor to the bulk variance. When examining apparent porosity and capillary water absorption—indicators of environmental sealing—coating type was identified as the key factor influencing the pore structure. The coating’s effect on apparent porosity was highly significant at 76.4% (*p* = 0.008), and it also dominated capillary water absorption at 67.6% (*p* = 0.035). Interfacial coatings made from natural pozzolans (pumice, perlite, diatomite), due to their high reactivity, effectively blocked capillary channels and micro-voids through secondary hydration or geopolymerization products, becoming the main determinants of these durability-critical parameters. WTA content had a secondary influence, with effects of 19.8% and 20.1% on porosity and water absorption, respectively. In comparison, the presence of PP fiber (3.8% porosity and 12.3% water absorption) did not have a statistically significant impact on matrix continuity. This comprehensive statistical analysis clearly confirms that in the hybrid geopolymer matrix, coating processes govern interface and impermeability, rubber volume affects structural lightness and strength limits, and synthetic fibers influence toughness.

#### Confirmation Experiments

The validation experiments in Table 7 demonstrate that two target combinations predicted by the Taguchi approach closely align with the experimental results. The deviation between the predicted and actual values remains within approximately 2.1–6.5% across all parameters, indicating that the model’s predictive performance is satisfactory for the tested factor levels.

In the optimum mixture determined for maximization purposes, the 28-day compressive strength was estimated at 15.5 MPa; however, the verification experiment yielded 16.2 MPa. The experimental value is approximately 4.3% higher than the estimate, indicating that the model slightly underestimated the compressive strength. Nevertheless, the selected combination of factors successfully delivered the expected mechanical performance.

Similarly, the measured flexural toughness was 114.3 N·mm, surpassing the estimated 106.9 N·mm. The roughly 6.5% difference indicates that the verification mixture absorbed more energy and had greater post-fracture deformation capacity than predicted. Consequently, both mechanical responses aimed at maximum performance exceeded expectations. This confirms the chosen mixture’s effectiveness in terms of compressive strength and post-fracture energy absorption.

In the mixture selected for minimization, the apparent porosity was estimated at 12.2%, whereas it was measured at 11.5% in the validation experiment. The experimental porosity, approximately 6.5% lower than the estimated value, indicates that the validation mixture formed a more compact internal structure than expected.

The estimated capillary water absorption was 7.8 kg/m^2^, closely matching the experimental value of 7.6 kg/m^2^. This small 2.6% discrepancy indicates that the Taguchi estimate is highly accurate for capillary water transfer. The slightly lower experimental result implies that the capillary pathways were somewhat more effectively confined than the model predicted.

In terms of oven-dry density, the estimated value was 1594 kg/m^3^, compared to a measured value of 1561 kg/m^3^, with a difference of 2.1%. This is the lowest estimation error among all validation parameters. The lower experimental density indicates that the lightness target was achieved slightly more successfully than predicted.

A key observation in the validation experiments is that the results exceeded the predicted values for both optimization goals.

- Maximized compressive strength and flexural toughness were higher than the predicted values,
- Minimized porosity, capillary water absorption, and density were lower than the predicted values.

The results demonstrate that the Taguchi main effect model offers acceptable predictive accuracy and experimentally verifies the targeted performance aspects of the chosen factor combinations. However, these validations apply to two separate design objectives. The mixture optimized for maximization should be assessed for mechanical capacity and toughness, whereas the mixture optimized for minimization should be evaluated for compactness, water transport, and lightness; they do not produce the same overall “general optimum.”

### 3.7. Microstructure Studies

The SEM images were not employed to validate the numerical marginal mean for any individual factor level or to isolate the coating type’s effect. Instead, they served exclusively as qualitative evidence of the overall microstructural state of chosen high-WTA formulations, helping to determine whether the observed morphologies aligned with the general macroscopic main-effect trends.

It should be noted that the microstructural interpretations in this section are based on SEM morphological observations; no EDS point analysis, elemental mapping, XRD or FTIR was performed on the hardened specimens. Therefore, references to C-(A)-S-H- or N-A-S-H-type phases should be understood as proposed mechanisms that are consistent with the observed morphology, the measured macroscopic trends and the established literature on alkali-activated fly ash/cement systems, rather than as direct phase identifications.

The SEM investigation focused on the 75% WTA series (M4, M8, M12 and M16), in which the interfacial and microstructural defects are most pronounced and therefore most clearly distinguishable, together with the reference mixture M17. It is acknowledged that, owing to the L16 orthogonal array, these mixtures also differ in PP fiber content (2%, 1%, 0.5% and 0%, respectively); consequently, the SEM-based comparisons are interpreted as qualitative indicators of the combined mixture state rather than as isolated evidence of the coating effect alone. Microstructural conclusions were drawn conservatively and cross-checked against the macroscopic property trends, with which they were found to be consistent.

It should be emphasized that the strengthening mechanism proposed in this section is supported not only by the qualitative SEM observations but also by the systematic quantitative macro-level evidence—namely, the statistically significant reductions in apparent porosity and water absorption and the density variations reported in Section 3.2 and confirmed by ANOVA (Table 6). Quantitative pore-area or crack statistics derived from SEM images were not pursued, as single two-dimensional fracture fields cannot representatively characterize the three-dimensional pore network of the composite; the SEM observations are therefore used herein as qualitative, complementary evidence only.

#### 3.7.1. The Microstructure of Thermally Cured Samples

**Figure 18:**
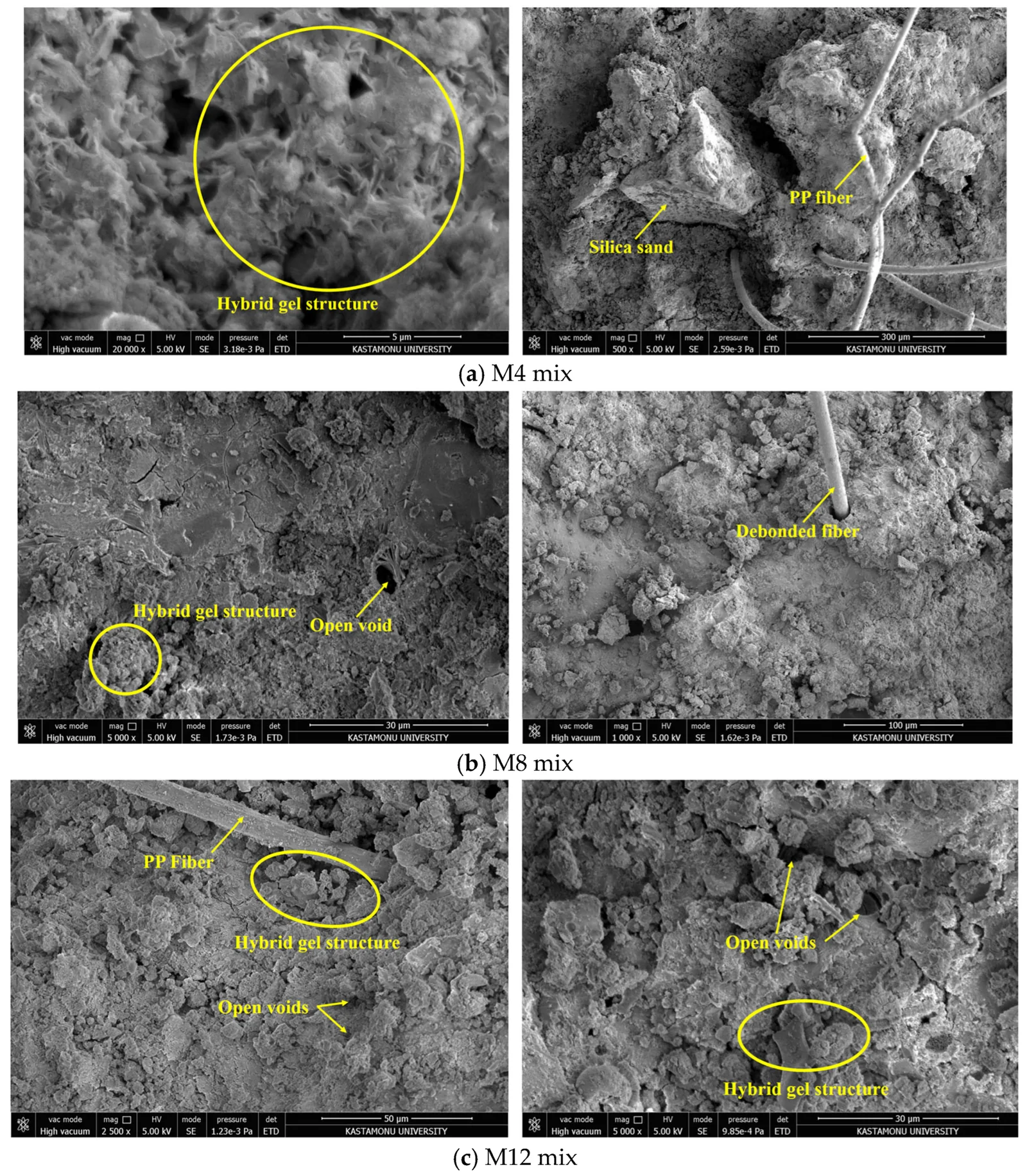

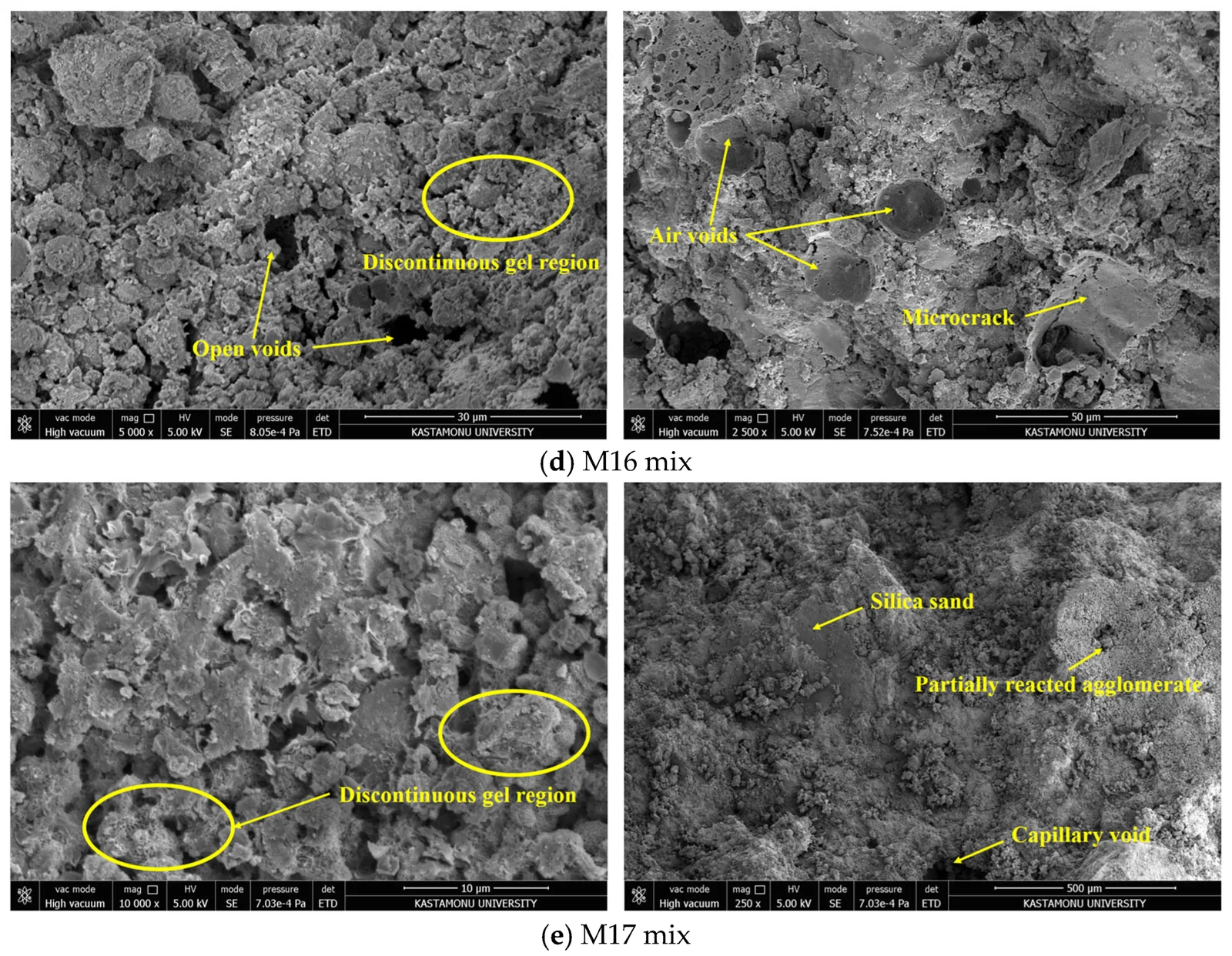
SEM images of the samples at 28 days.

Sample M4 (uncoated WTA, 75% WTA, 2% PP fiber) exhibits a distinctly heterogeneous and discontinuous matrix (Figure 18a). The images show that a hybrid gel structure has developed in some areas, but open voids, weak interfaces, and loss of adhesion around the fibers persist. Considering the uncoated WTA’s formation of a weak ITZ, the high WTA ratios’ disruption of matrix continuity, and the 2% PP fiber’s significant reduction in fresh workability, this microstructure is consistent with the limited mechanical development, high capillary water absorption, and weak zones due to fiber agglomeration observed in the uncoated series. Therefore, the open voids and debonded fiber morphology observed in M 4 result from poor placement and low compaction caused by the high rubber volume and high fiber content. In sample M8 (WTA coated with pumice, 75% WTA, 1% PP fiber), a denser and more cohesive hybrid gel matrix is observed compared to M4 (Figure 18b). The amorphous silica content of the pumice coating promotes the formation of secondary reaction products in the ITZ and limits pore connectivity. However, at the 75% WTA level, the structure is not entirely compact; local open voids and fiber-matrix separation remain. This observation is consistent with experimental results showing that, although the pumice-coated series exhibits the lowest porosity/water absorption and the highest compressive-flexural strength, a very high WTA ratio still leads to interfacial weakness. In summary, the coating effect in M8 is predominantly beneficial, but a high WTA content remains a limiting factor. In sample M 12 (perlite- coated WTA, 75% WTA, 0. 0.5% PP fiber), the hybrid gel phase exhibits a more balanced appearance than in M4, though it is not as dense as in M8 (Figure 18c). Although open voids are still present, the more controlled distribution of the 0. 0.5% PP fiber prevents the pronounced distortion around the fibers observed in M 4. The improvement of the ITZ by the perlite coating and the consolidation of the matrix by the 0. 0.5% PP fiber at an optimal dosage explain the development of a relatively more balanced microstructure in this mixture. However, due to the 75% WTA content, the porous structure has not completely disappeared. This SEM image is consistent with the perlite series exhibiting better flexural strength and lower sorptivity than the reference. It indicates that the 0.0% and 0.5% PP fiber levels are the most suitable fiber contents from both mechanical and physical perspectives.

Sample M16, coated with diatomite and containing 75% WTA and no PP fiber, shows one of the weakest microstructures (Figure 18d). Clear features such as discontinuous gel regions, open voids, air pockets, and microcracks are visible. The lack of fibers removes the crack-bridging mechanism, while diatomite’s very fine, high-surface-area structure appears to consume free mixing water quickly, hindering proper gel formation. This aligns with the experimental data, as the diatomite series exhibits the smallest spread diameter, the highest porosity and sorptivity, and the lowest compressive-bending strength. The observed microstructural features—discontinuous gel, voids, and cracks—are direct results of insufficient compaction and weak matrix continuity. In contrast, the reference mixture without WTA or PP fibers exhibits a more uniform, hybrid binder matrix (Figure 18e). Although capillary voids and partially reacted agglomerates are seen, there is no fiber-induced separation or interface weakness as in M4 and M16. Nonetheless, the reference matrix is not fully dense; hybrid gel continuity is limited, and capillary voids remain. While more ordered internally than problematic multi-phase systems, it is less compact than the pumice- and perlite-coated series, consistent with its moderate strength and higher porosity and sorptivity.

SEM images showed that the microstructural quality after 28 days of thermal curing was highest in M8, followed by M12 and M17, whereas M4 and especially M16 exhibited weaker, more discontinuous matrix structures. Coatings with pumice and perlite enhanced the ITZ, promoting the formation of a denser hybrid gel. In contrast, diatomite resulted in weaker phases and a porous structure due to its high water demand. Moreover, a high WTA ratio reduced interfacial continuity across all mixtures; however, PP fiber strengthened the matrix only at an optimal dose. Overall, the SEM results confirm the key trends observed in the mechanical, physical, and transport properties.

#### 3.7.2. The Microstructure of Samples Exposed to 600 °C

**Figure 19:**
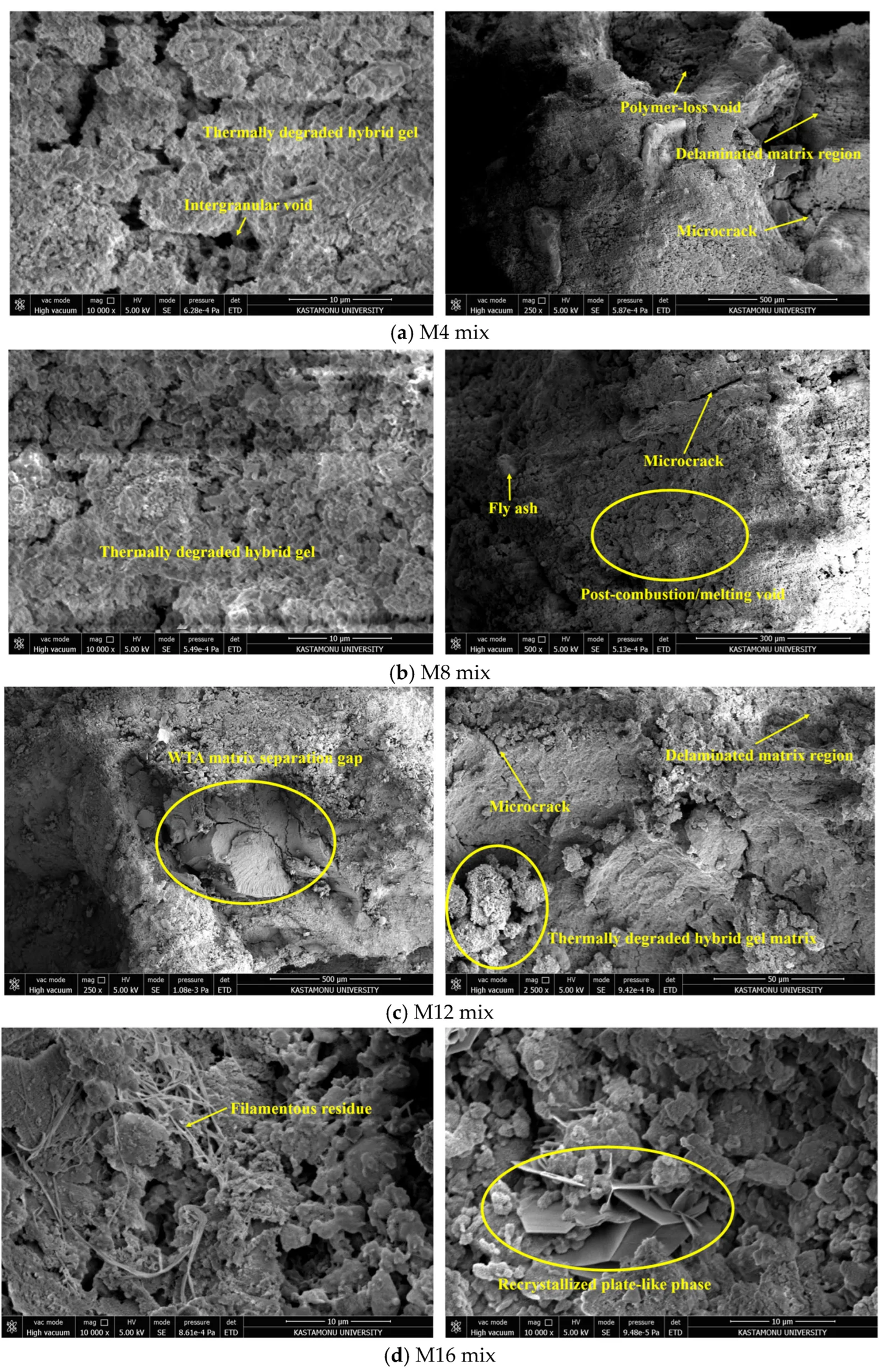

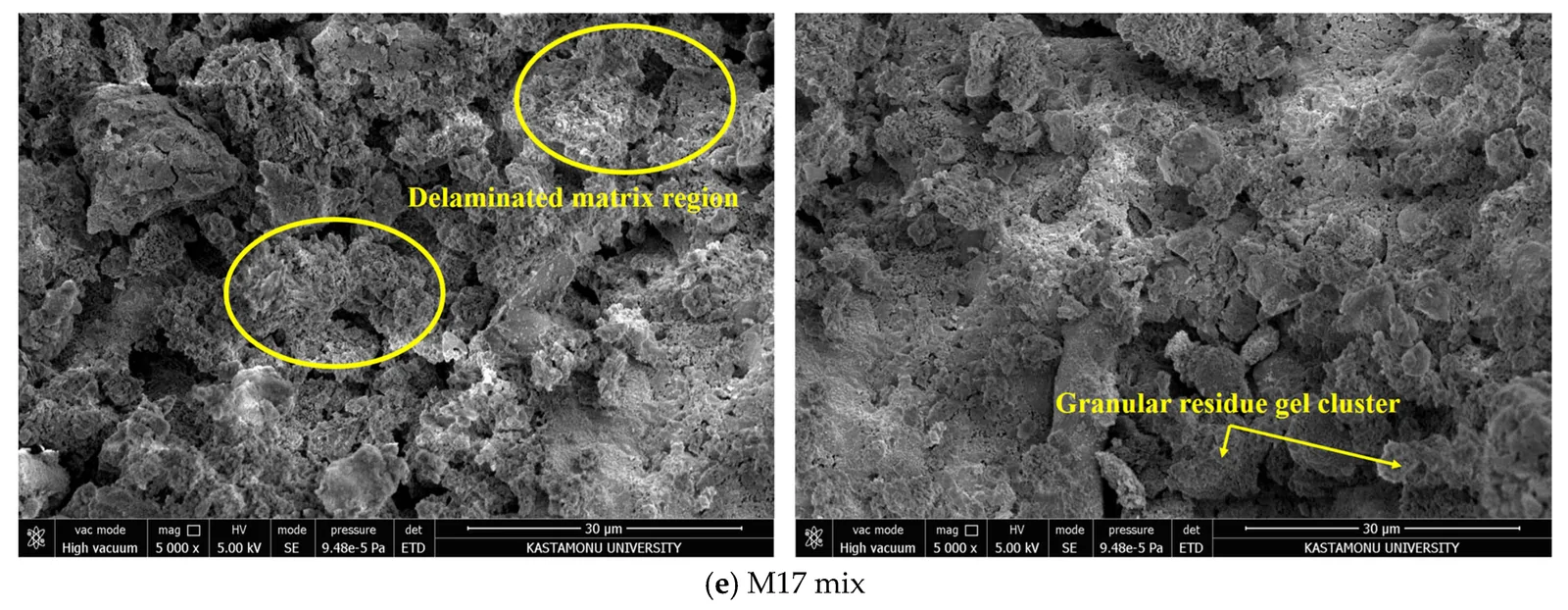
SEM images of the samples after 600 °C.

Figure 19a (M4 mixture). SEM images of the M4 sample exposed to 600 °C show significant thermal degradation of the matrix, which has fragmented the previously more continuous hybrid gel structure into a discontinuous form. The observed hybrid gel breakdown and intergranular voids are due to dehydration, shrinkage, and loss of gel continuity in the C-(A)-S-H/N-A-S-H-based binder phases. Additionally, voids labeled as “polymer-loss voids” result from degradation of the polymer phase due to a high WTA ratio (75%) and a 2% PP fiber content, forming an internal void network. At 75% WTA, compressive strength decreases to 9.7 MPa, and mass loss reaches 22.9% at 600 °C. Increasing PP fiber content to 2% raises mass loss to 24.1% and limits residual strength to 10.3 MPa. This suggests that melting and vaporization of PP fibers, combined with WTA degradation, create new capillary channels and macro-voids in the matrix. The delaminated matrix region and microcracks observed in the right image further support this: they indicate that damage following high-temperature exposure primarily involves gel breakdown, loss of the polymer phase, interfacial separation, and crack growth. Figure 19b (M8 mixture). SEM images of the M8 sample exposed to 600 °C show that, thanks to the pumice coating, the matrix remained more intact compared to M4; however, significant structural damage still occurred due to the high WTA (75%) and thermal degradation. The marked area of thermally degraded hybrid gel indicates that the hybrid binder phase became partially discontinuous following thermal dehydration and shrinkage. Additionally, the post-combustion/melting voids and microcracks indicate the formation of new voids and crack paths within the matrix, resulting from WTA combustion and the loss of the polymer phase. The pumice-coated series demonstrates the best high-temperature performance at 600 °C, with a compressive strength of 15.6 MPa and a mass loss of 15.9%, suggesting that the pumice coating helps limit thermal degradation by enhancing the ITZ. Nonetheless, because M8 contains 75% WTA, porosity could not be entirely prevented despite the beneficial effects of the pumice coating. Figure 19c (M12 mixture). Despite the pearlite coating, the matrix integrity of the M12 sample has notably weakened after exposure to 600 °C. SEM images show debonding gaps, microcracks, and delaminated regions, indicating that interface separation, delamination, and crack growth are prevalent under thermal stresses. The areas marked as “thermally degraded hybrid matrix” suggest that the hybrid gel phase has fractured and become discontinuous. This morphology aligns with the observed decrease in compressive strength to 8.5 MPa and the increase in mass loss to 22.3% in the perlite-coated series at 600 °C. Although a 0.5% PP fiber content is generally considered to provide some improvement in the literature and in experiments, the thermal resistance of this mixture remains limited due to the high void volume resulting from 75% WTA and the perlite’s internal porosity.

Figure 19d (M16 mixture). The M16 sample exposed to 600 °C exhibits one of the weakest microstructures at high temperatures. The filamentous residue structures in the images represent fine residual formations left behind after the thermal decomposition of the organic phase. In contrast, the regions labeled as “recrystallized plate-like phase” indicate plate-shaped residual phases formed by recrystallization at high temperatures. These structures reveal that the gel continuity in the diatomite-coated system has been lost, and the matrix has transformed into a looser, more porous residual phase. Experimentally, the diatomite-coated series exhibits the lowest compressive strength, 8.2 MPa at 600 °C, and one of the highest mass losses, 23.3%, supporting this observation. The very fine, high-surface-area structure of diatomite initially led to greater water retention and higher vapor pressure during heating; this, in turn, accelerated cracking, porosity formation, and structural dissolution within the matrix. Figure 19e (M17 reference mixture). SEM images of the reference mixture after 600 °C show that there are no macro-voids due to polymer loss in the system lacking WTA and PP fibers; conversely, the binder matrix has still undergone significant thermal degradation. The delaminated matrix regions visible in the images indicate that the gel phase has separated into layers; the granular residue gel cluster structures, on the other hand, reveal that the binder phase remains as granular residue clusters. This observation is consistent with the reference mixture having a compressive strength of 8.7 MPa and a mass loss of 22.6% at 600 °C. In other words, although there is no polymer-phase-induced voiding in the reference system, the matrix’s integrity has been significantly compromised due to dehydration, shrinkage, and granularization of the hybrid gel structure.

In summary, SEM images at temperatures above 600 °C show that the most stable microstructure forms in the pumice-coated M8 mixture. In contrast, voiding, layering, and interfacial separation become more significant in the M12 and especially the M16 mixtures. Since the M17 reference mixture lacked a polymer phase, it did not develop combustion-induced macrovoids. Instead, it formed a granular residual matrix resulting from the thermal degradation of the hybrid gel structure. Thus, the SEM results align with the trends observed in compressive strength and mass loss during high-temperature tests.

#### 3.7.3. The Microstructure of Samples Exposed to H_2_SO_4_

**Figure 20:**
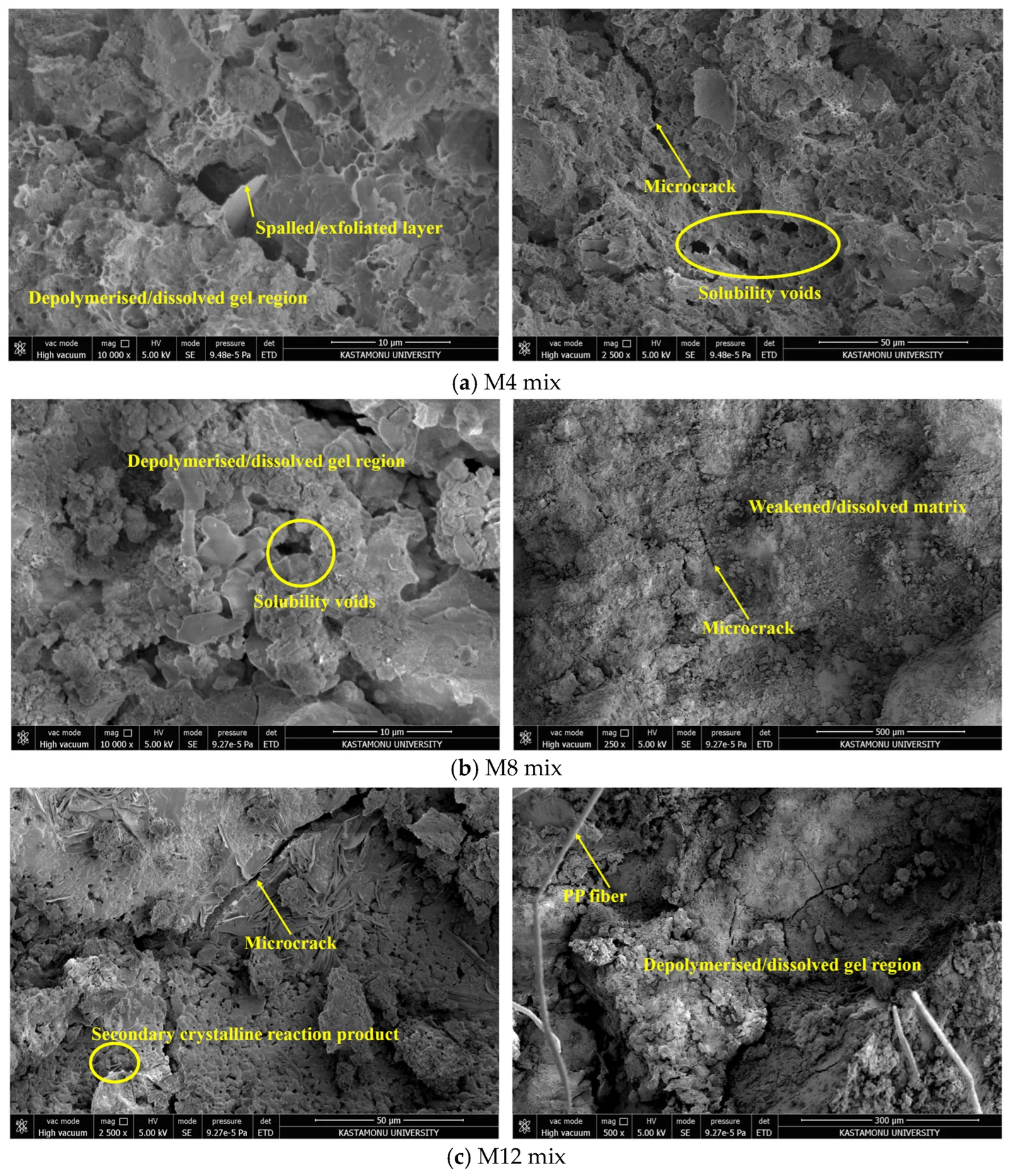

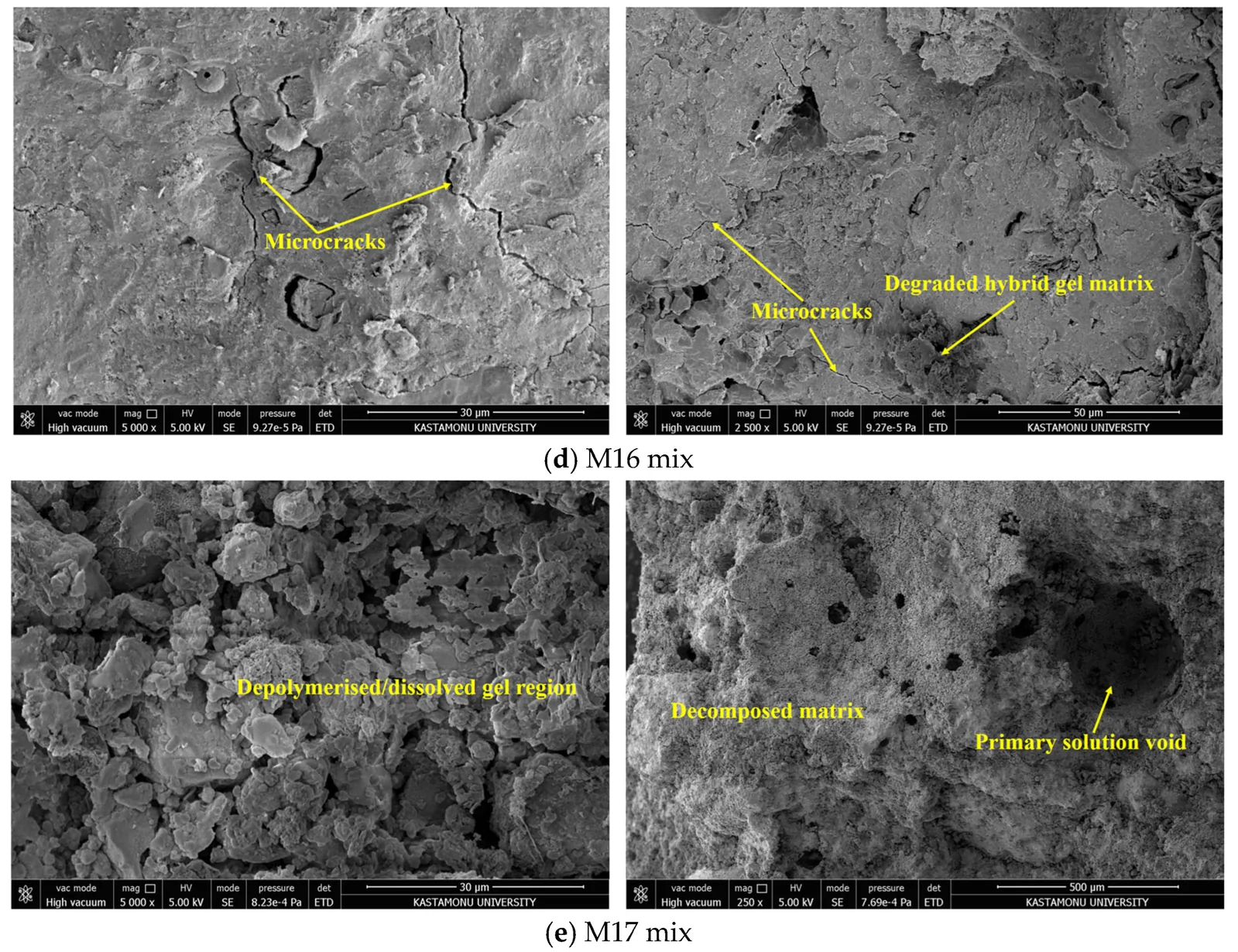
SEM images of the samples after exposure to H_2_SO_4_.

Figure 20a shows the M4 mixture. In the H_2_SO_4_-exposed M4 sample, the matrix has significantly depolymerized; the binder phase has dissolved, resulting in loss of continuity and surface-spalled or exfoliated layers. Microcracks and solubility voids indicate increased dissolution voids and crack networks resulting from acid attack. This aligns with calcium-containing phases reacting with H_2_SO_4_ to form expanding products that cause outer-layer detachments. The high WTA content (75%) in the M4 weakens the carrier matrix, accelerating dissolution and delamination; at 75% WTA, the compressive strength decreases to 6.2 MPa, and the mass loss reaches 15.9%. Overall, Figure 20a shows that in the uncoated system with high rubber content, acid exposure primarily causes gel dissolution, surface peeling, and the development of cracks and voids. Figure 20b (M8 mixture): While the pumice coating helps the M8 sample maintain a more cohesive matrix compared to M4—thanks to its high amorphous silica content—the images still show depolymerized or dissolved gel regions, solubility voids, a weakened or dissolved matrix, and microcracks. This suggests that the pumice coating enhances the ITZ and capillary voids to some degree, yet the 75% WTA and 1% PP fiber content still create significant weak zones under acid exposure. Experimental results confirm that the pumice-coated series exhibits the best acid resistance, with compressive strength increasing to 6.8 MPa and mass loss decreasing to 13.9%. Nevertheless, due to the high WTA levels in M8, some dissolution voids persist, the matrix remains weakened in certain areas, and cracks continue to develop. Therefore, Figure 20b illustrates that while the pumice coating mitigates acid attack, the high rubber content and fibers limit its overall effectiveness—see Figure 20c (M12 mixture). In the M12 sample, when the structures labeled as microcracks, secondary crystalline reaction products, PP fibers, and depolymerized/dissolved gel regions are considered together, it is evident that both matrix dissolution and the formation of localized secondary crystalline structures occur under acid influence. Here, a 0.5% PP fiber content appears to be the optimal dosage that limits the complete propagation of microcracks; however, due to the 75% WTA ratio, the porous structure with weak interfaces cannot be eliminated. Indeed, in the H_2_SO_4_ results, it was noted that with the addition of 0.5% PP fiber, the compressive strength increased to 7.9 MPa and the mass loss decreased to 15.5%, indicating that the fiber forms a protective skeleton to a certain extent. Conversely, the high WTA ratio facilitated the acid solution’s access to larger surface areas and accelerated the dissolution of the gel phase. Therefore, Figure 20c demonstrates that the acid effect in M12 progresses alongside microcrack development, partial fiber bridging, and the dissolution of the gel phase.

Figure 20d (M16 mixture). The M16 sample shows some of the weakest resistance to acid attack. Microcracks and evidence of a degraded hybrid gel matrix in the images clearly indicate that the binder phase has lost its structural integrity, with widespread cracking resulting from acid exposure. The lack of fibers removes the crack-bridging mechanism, while the highly porous diatomite structure allows acid to penetrate more easily. Experimentally, the diatomite-coated series had the lowest compressive strength (6.1 MPa) and the highest mass loss (18.7%) under H_2_SO_4_, supporting this conclusion. Thus, Figure 20d illustrates that acid primarily caused widespread gel degradation, crack formation, and matrix loosening, thereby impairing its load-bearing capacity. Figure 20e (M17 reference mixture). Since this mixture contains no WTA or PP fibers, the acid effect is more directly visible on the binder matrix. The images show a depolymerized/dissolved gel region, decomposed matrix, and primary solution voids, indicating that the hybrid gel network dissolves and breaks down under H_2_SO_4_, creating primary dissolution voids. The reference sample’s compressive strength of 6.3 MPa and mass loss of 16.7% align with this microstructural deterioration. Although there is no polymer-related interfacial issue in the reference system, the acid weakens the load-bearing structure by directly dissolving the binder. Therefore, Figure 20e demonstrates that H_2_SO_4_ primarily causes gel depolymerization, matrix degradation, and dissolution void formation.

When the SEM images taken after H_2_SO_4_ exposure are evaluated together, the common damage mechanisms across all samples include gel depolymerization/dissolution, microcrack formation, dissolution voids, and surface delamination. In terms of coating type, the pumice-coated M8 exhibited the most balanced microstructure, whereas the diatomite-coated M16 showed the most pronounced dissolution and crack damage. M12 provided partial crack control with the addition of 0.5% PP fiber; however, due to the 75% WTA, it still exhibited a porous, weak structure. These findings are consistent with the H_2_SO_4_ test results, in which the pumice coating performed best and the diatomite coating performed worst, and with the mechanical and mass-loss results, which indicate that a lower fiber dosage is more advantageous.

#### 3.7.4. The Microstructure of Samples Exposed to MgSO_4_

**Figure 21:**
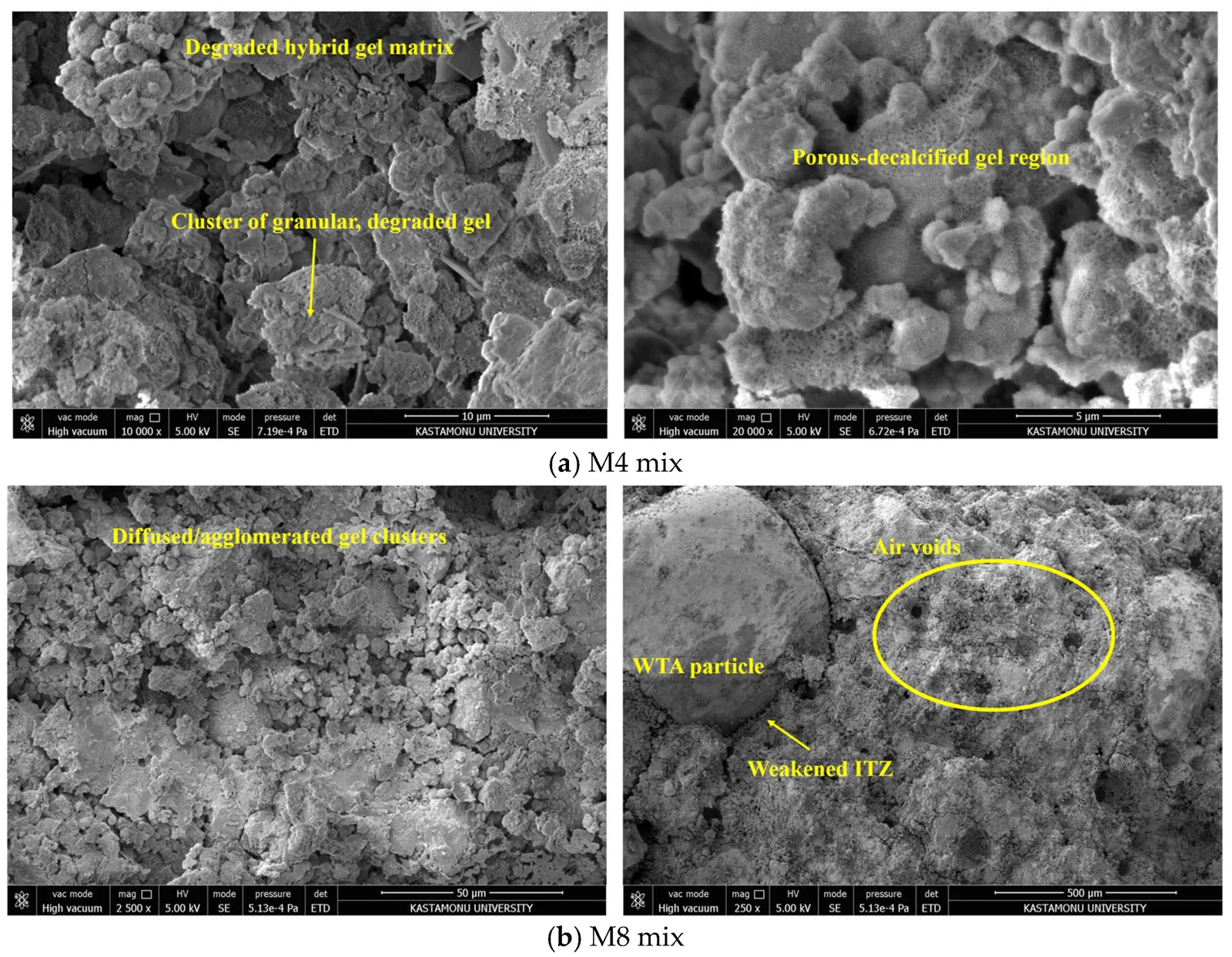

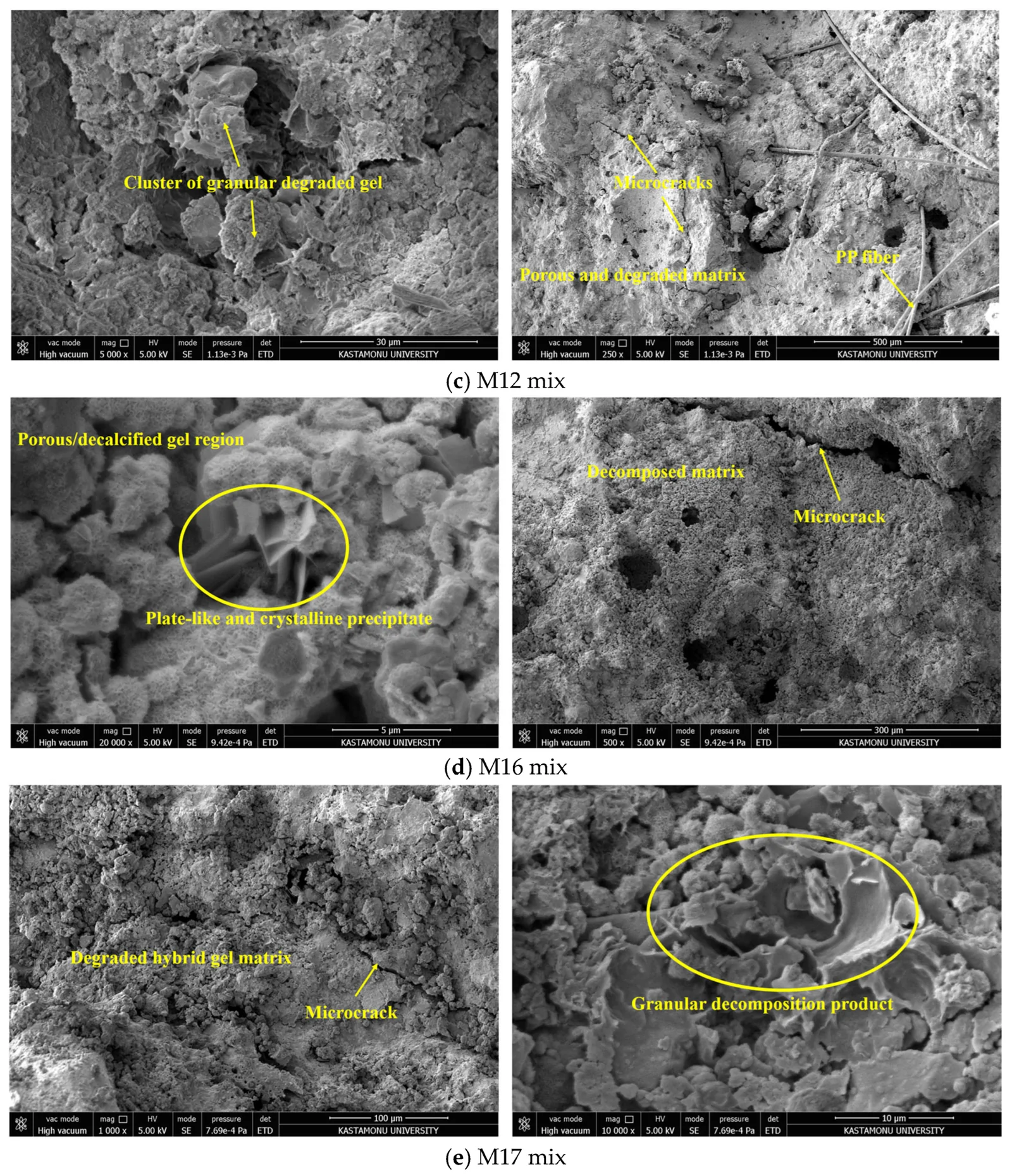
SEM images of the samples after exposure to MgSO_4_.

In the M4 sample, the matrix becomes granular after sulfate treatment, disrupting the hybrid gel’s continuity and forming porous, decalcified regions (Figure 21a). While the hydrophobic WTA coating limits sulfate ingress somewhat, the mixture containing 75% WTA and 2% PP fiber results in weak interfaces and loose gel clusters. This is consistent with the observed reduction in tensile strength to 8.1 MPa and an increase in mass loss to 10.3% at high WTA levels, despite limited sulfate damage at low rubber contents. Additionally, experimental results show that a high fiber volume promotes sulfate ingress by causing agglomeration and creating weak air voids in the fresh state. The M8 mixture shows that, despite the pumice coating, images reveal dispersed or agglomerated gel clusters, air voids, a weakened ITZ, and a clear WTA particle perimeter (Figure 12b). Although the pumice coating generally offers the best sulfate resistance, combining 75% WTA with 1% PP fiber has once again compromised the matrix’s continuity. While the pumice-coated sample performed best at 12.2 MPa, with an 8.8% mass loss, the high WTA content and fiber level facilitated the migration of sulfate ions to the interfacial regions. Thus, the pumice coating’s benefits persisted in M8, but a fully compact structure was unattainable due to the rubber’s large volume. The M12 sample’s SEM images show PP fibers, granular degraded gel clusters, microcracks, and a porous, fragmented matrix (Figure 12c). Here, 0.5% PP fibers seem optimal for limiting crack propagation; however, because of the looser internal structure with 75% WTA and perlite coating, the matrix remains porous and brittle. Experimentally, adding 0.5% fibers increased the compressive strength to 10.5 MPa and reduced the mass loss to 9.7%, whereas the perlite-coated group exhibited poorer sulfate resistance, with a compressive strength of 7.8 MPa and a mass loss of 12.2%. Therefore, while fibers partly bridge cracks in M12, the structure with perlite and a high W/C ratio cannot fully maintain integrity under sulfate attack.

M16 mixture. The M16 sample exhibits one of the most pronounced microstructural degradations under the influence of MgSO_4_ (Figure 21d). The images reveal porous/decalcified gel regions, microcracks, a disintegrated matrix, and localized plate-like and crystalline precipitates. These precipitates are consistent with Mg-rich secondary products formed under the influence of MgSO_4_ that lack binding properties, further weakening the gel’s integrity. The high specific surface area and porous structure of diatomite facilitated the transport of sulfate ions into the interior, and the absence of fibers also eliminated the crack-bridging mechanism. This scenario is consistent with a drop in compressive strength to 7.9 MPa and a 10.0% mass loss in the diatomite-coated group. M17 reference mixture. Since the reference mixture contains no WTA or PP fibers, the effect of sulfate is observed more directly on the binder matrix (Figure 21e). The images clearly show a degraded hybrid gel matrix, microcracks, and granular disintegration products. Thus, although there are no polymer-induced interfacial voids in the reference system, the matrix integrity has been compromised by decalcification and granularization of the hybrid gel phase induced by MgSO_4_. This appearance is consistent with the reference mixture’s compressive strength of 7.9 MPa and mass loss of 10.2%. Consequently, M17 exhibits a relatively more homogeneous yet distinctly dissolved and weakened binder skeleton under sulfate conditions.

The common damage mechanism observed across all samples after exposure to MgSO_4_ can be summarized as follows: decalcification/dissolution of the hybrid gel phase, formation of granular degradation products, development of microcracks, and increased pore connectivity. In this context, the pumice-coated M8 exhibits the most balanced microstructure, whereas M12 and especially M16 show a looser matrix that is more susceptible to sulfate attack. Although the hydrophobic effect of uncoated WTA in the M4 mixture provided some protection, the high WTA and fiber content limited this advantage. M17, on the other hand, exhibited a purer degradation morphology because it lacked a polymer phase. These SEM findings are in direct agreement with the experimental results, which show an inverse relationship between compressive strength and mass loss.

#### 3.7.5. The Microstructure of Samples Exposed to the F-T Effect

**Figure 22:**
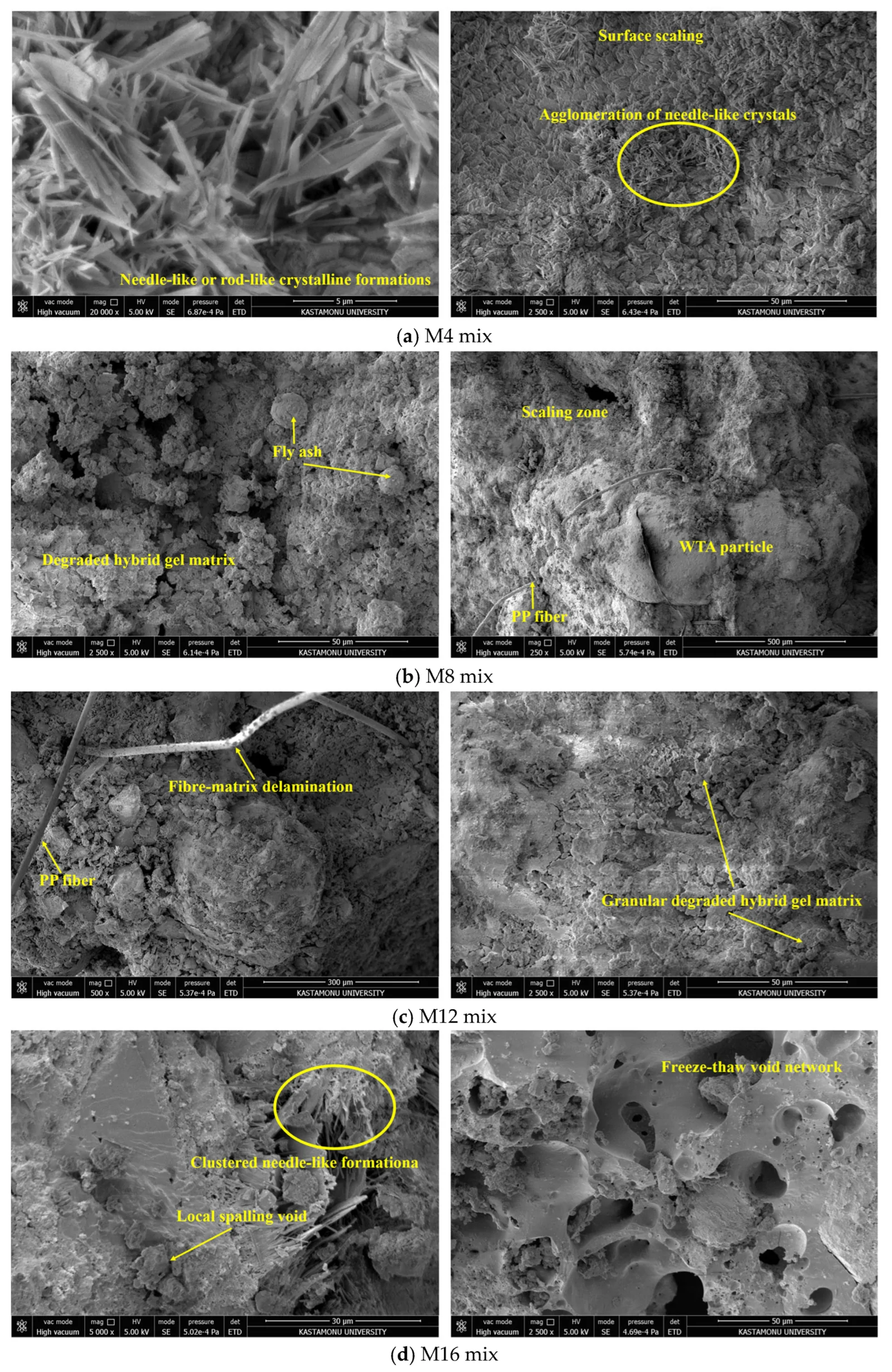

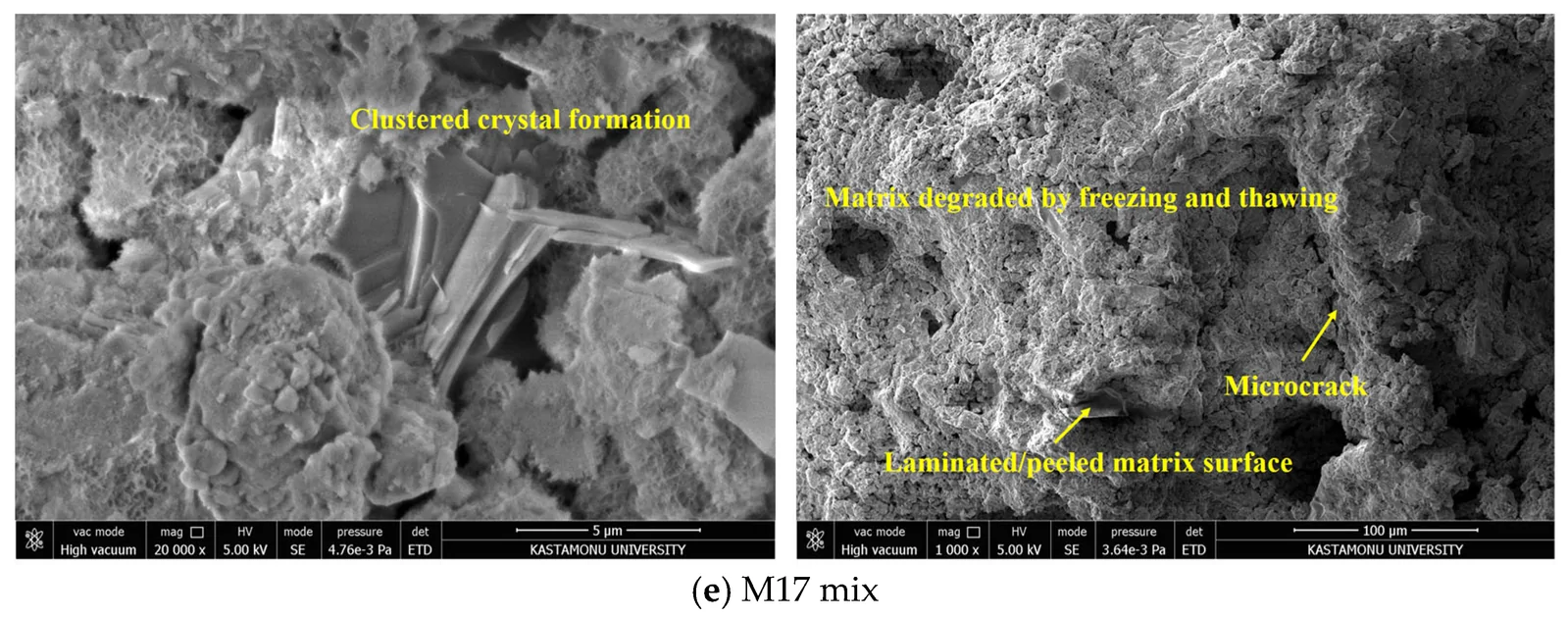
SEM images of the samples after exposure to the F-T effect.

SEM images of sample M 4 show that the matrix has been significantly degraded after freeze–thaw cycles, with needle- and rod-like crystalline formations clustering on the surface and surface scaling (Figure 22a). In particular, regions labeled “agglomeration of needle-like crystals” indicate the development of secondary crystalline formations around voids and cracks. Additionally, the high 75% WTA ratio and 2% PP fiber content have weakened matrix continuity, increased void connectivity, and accelerated surface peeling. This appearance is consistent with experimental trends in uncoated, high-fiber systems under freeze- thaw cycles, which show microcrack development, surface delamination, and void coalescence. In the M 8 sample, although a more balanced internal structure was maintained compared to M 4 due to the pumice coating, SEM images still reveal a degraded hybrid gel matrix, a scaling zone, WTA particles visible, and PP fibers (Figure 22b). This indicates that while the pumice coating improves the ITZ and enhances matrix integrity, freeze- thaw damage cannot be completely prevented because the sample still contains 75% WTA. Surface scaling and localized gel degradation suggest that freeze–thaw-induced internal stresses remain significant even in the pumice-coated group at high WTA levels. This observation reveals that, despite the pumice-coated series demonstrating the best freeze–thaw performance, damage still accumulates in the structure due to the high rubber content. The most striking finding in the M 12 specimen is fiber-matrix delamination developing around the PP fiber, accompanied by a granular, degraded hybrid gel matrix (Figure 22c). This morphology indicates that repeated expansion and contraction during freeze–thaw cycles result in a loss of adhesion at the fiber-matrix interface. However, since M 12 contains 0.0.5% PP fiber, the crack-bridging effect has not completely disappeared; that is, the fiber limits microcrack propagation to some extent. Nevertheless, the 75% WTA level partially limited the fiber’s positive effect by increasing void connectivity within the matrix. Therefore, the structure observed in M 12 supports the experimental trend that a low/medium fiber dosage yields a more balanced freeze–thaw behavior. Yet, a high WTA still tends to exacerbate damage.

Sample M16 exhibits one of the weakest microstructures under freeze–thaw conditions (Figure 22d). The images clearly show clustered needle-like formations, localized spalling voids, and, in particular, an extensive freeze–thaw void network. Furthermore, due to the absence of fibers, the crack-bridging mechanism has been eliminated; in this diatomite-coated system with high WTA, voids have more readily coalesced to form a continuous network. Consequently, freeze–thaw damage in M16 has primarily progressed through the development of the void network, local spalling/peeling, and matrix loosening. This microstructure is directly consistent with experimental results showing that the diatomite-coated series exhibits higher porosity and lower compressive strength. SEM images of the reference mixture reveal structures such as clustered crystals, a matrix degraded by freezing and thawing, microcracks, and a laminated/peeled matrix surface (Figure 22e). Since M17 contains no WTA or PP fibers, damage is more directly observed in the binder matrix. In other words, although there is no specific weakness at the rubber-matrix interface, delamination, peeling, and microcracks have developed on the matrix surface due to internal pressures from water in saturated voids during freezing and thawing. Therefore, while M17 exhibits a more homogeneous damage morphology than systems containing WTA, it still shows distinct freeze–thaw degradation, particularly in surface delamination and microcrack development.

When evaluating the SEM images in Figure 22, it is apparent that the common damage mechanisms after 90 freeze–thaw cycles include loosening or degradation of the gel matrix, microcrack development, surface scaling and spalling, increased porosity, and the formation of secondary crystalline structures in some areas. Among the coating types, pumice-coated M8 exhibits a more stable internal structure, whereas diatomite-coated M16 shows a more extensive void network and localized delamination. M12 shows partial crack control due to the low PP fiber content, though fiber-matrix separation is still observed. In M4, the absence of coating on WTA and high fiber content led to more significant crystalline clustering and scaling. These SEM results clearly highlight the significant influence of coating type, WTA ratio, and PP fiber dosage on residual strength, mass loss, and internal structural stability during freeze–thaw cycles.

## 4. Conclusions

This study showed that single-part hybrid geopolymer composites incorporating coated waste-tire aggregates can be effectively produced using a fixed-matrix Taguchi L16 design. Their performance depends on the main effects of coating type, WTA replacement ratio, and PP fiber dosage (note: the L16 array does not resolve factor interactions):

1. The results confirm that the performance of one-part hybrid geopolymer composites containing waste tire aggregate is primarily governed by the quality of the rubber–matrix interface, the WTA replacement level, and the PP fiber dosage. Within this framework, pumice was the most effective coating material, consistently providing the best overall balance of fresh-state behavior, strength, transport resistance, and durability.
2. Among the tested WTA levels, 10% WTA yielded the most optimal responses in direct factor-level assessments for compressive strength, flexural strength, toughness, capillary water absorption, and various durability metrics. Increasing the WTA content to 75% tended to weaken the matrix overall, primarily due to a decrease in load-bearing solid volume, poorer interfacial continuity, and increased pore connectivity.
3. The effect of PP fiber varied significantly with response. A 0.5% PP fiber content resulted in the highest 28-day compressive strength, along with overall good matrix integrity and durability responses. Conversely, 2% PP fiber achieved the highest flexural strength and toughness but significantly lowered workability and raised concerns about fiber clumping and trapped voids. Thus, neither level of fiber can be considered the best in all aspects.
4. High-temperature exposure showed a dual response. Moderate heating produced a secondary curing effect in several mixtures, whereas heating to 600 °C caused marked deterioration via gel dehydration, PP fiber melting, and WTA pyrolysis. Under these conditions, pumice-coated mixtures exhibited the highest thermal stability, while low WTA and PP fiber contents were more effective at limiting mass loss and preserving residual strength.
5. Under freeze–thaw, MgSO_4_, and H_2_SO_4_ exposures, the same governing trend held: mixtures with a denser matrix and a better-engineered ITZ performed more favorably. In this regard, pumice coating exhibited the most stable durability response. In contrast, diatomite generally produced the weakest performance due to its excessive water demand, high specific surface area, and tendency to form porous, weak phases.
6. The Taguchi assessment tailored to specific responses did not find a universal optimum for all performance metrics. Pumice remained the most consistently preferred coating material, while 10% WTA showed the strongest positive trend across multiple mechanical and transport responses. The optimal PP fiber dosage varied based on the application: 0.5% favored compressive strength and durability aspects, whereas 2% was better for flexural strength and energy absorption after cracking.
7. It should be acknowledged that the proposed production route involves energy- and chemical-intensive steps- including the 12 M NaOH pretreatment, the 75 °C conditioning of the coated aggregates, the 45 °C thermal curing, and the use of 400 kg/m^3^ Portland cement, 120 kg/m^3^ sodium metasilicate and 40 kg/m^3^ PCE—whose environmental impacts may partly offset the benefits of waste tire valorization and natural pozzolan use. A comprehensive life-cycle assessment (or at least an inventory analysis) quantifying these impacts was beyond the scope of the present study and is identified as an essential next step before the material can be classified as low-carbon or environmentally preferable.
8. The 28-day compressive strengths obtained in this study (approximately 9–15.5 MPa) define the realistic application window of the developed composites. These values do not meet the requirements of structural load-bearing concrete; however, they exceed the minimum compressive strength requirements for non-load-bearing concrete masonry units (typically ≥4.1 MPa per ASTM C129 [117]) and are comparable to those of commercial lightweight blocks, partition panels, and insulating elements. Considering their low density (down to ~1570 kg/m^3^), ductility-enhanced fracture behavior, and impact/energy dissipation capacity provided by the rubber phase, the most suitable target applications for the developed composites are non-structural masonry blocks, interior partition panels, thermal insulation units, and vibration- or impact-damping layers. Any structural use would require a substantial increase in strength beyond the levels achieved here.
9. Choosing the right PP fiber dosage depends on the specific application. Low fiber amounts are better when the main goals are compressive strength, matrix density, and durability. In contrast, higher fiber levels benefit situations that need improved flexural performance, crack bridging, and energy absorption. Therefore, PP fiber content should be considered an adjustable feature in design, not simply good or bad.

**Limitations and future work:** Several limitations of this study should be acknowledged. (i) The Taguchi L16 orthogonal array does not allow the resolution of factor interactions; all conclusions are therefore restricted to main-effect trends, and the reported factor-level values represent marginal means rather than directly comparable formulations. (ii) The partition of the coating powder between the adhered layer and free fines, and the resulting effective water-to-solids ratio, could not be quantified exactly; fresh densities and air contents of the mixtures were not systematically measured. (iii) The microstructural evidence is qualitative and based on SEM morphology only: no EDS/elemental mapping, XRD or FTIR analyses were performed on the hardened composites, no particle-level SEM/EDS characterization of the coated aggregates was carried out, and the SEM mixtures (75% WTA series) also differed in fiber content. (iv) The freeze–thaw test was a modified protocol rather than a standard ASTM C666 [55] test, and the relative dynamic modulus and durability factor were not measured; the pH of the chemical exposure solutions was not monitored. (v) The fracture tests were terminated at a 10% post-peak load drop, so the reported toughness values are lower-bound comparative estimates. (vi) No TGA/gas analysis was performed to verify the thermal decomposition of the rubber phase. (vii) No life-cycle or inventory analysis was conducted; therefore, the environmental impacts of the additional chemicals, heat, and water required by the coating and curing processes could not be quantified, and environmental claims are restricted accordingly. Future work will address these limitations through matched confirmation mixtures, EDS/XRD/FTIR-based interface characterization, particle-level coating analysis, and a comprehensive life-cycle assessment of the proposed production route.

## Figures and Tables

**Table 1:** Chemical properties of binding and coating materials.

Materials	CaO	SiO_2_	Al_2_O_3_	Fe_2_O_3_	MgO	SO_3_	Na_2_O	K_2_O	LOI *
CEM II/B-S	48.1	30.8	7.3	1.8	5.2	2.5	0.3	0.8	0.9
UK	4.3	61.3	22.6	6.2	0.3	0.5	0.4	0.6	1.3
Pumice	1.5	67.8	11.7	1.8	0.9	0.1	0.4	1.1	0.9
Perlite	0.8	72.7	13.8	0.6	0.2	0.2	0.3	0.9	2.8
Diatomite	2.8	82.5	6.5	0.9	1.4	0.2	0.3	0.9	8.9

* Loss on ignition.

**Table 2:** Chemical composition of sodium metasilicate.

SiO_2_	Na_2_O	Purity (%)	Moisture Content (%)	Silica Module (MS)
47.6	48.9	>95	<2	0.97

**Table 3:** Technical specifications of PPF.

Specific Gravity	Length (mm)	Diameter (µm)	Tensile Strength (MPa)	Elastic Modulus (MPa)	Elongation at Break (%)	Melting Point (°C)
0.91	12	24	400	9000	70	175

**Table 4:** Variables according to the Taguchi L16 experimental design matrix.

Variables	Level 1	Level 2	Level 3	Level 4
Coating type	Uncoated	Pumice-coated	Perlite-coated	Diatomite-coated
WTA content (%)	10	25	50	75
PP fiber content (%)	0	0.5	1	2

**Table 5:** Mixing ratios and material quantities.

Mix ID	Mixing Ratio (%)			Amounts of Materials (kg/m^3^)							
	Coating Type	WTA Content	PP Fiber Content	UK	Cement	Water	PCE	Alkali Activator	Silica Sand	WTA	PP Fiber
M1	Uncoated	10	0	400.0	400.0	280.0	40.0	120.0	789.0	32.0	0.0
M2	Uncoated	25	0.5	400.0	400.0	280.0	40.0	120.0	688.0	84.3	4.6
M3	Uncoated	50	1	400.0	400.0	280.0	40.0	120.0	451.9	166.1	9.1
M4	Uncoated	75	2	400.0	400.0	280.0	40.0	120.0	219.1	241.7	18.2
M5	Pumice	10	0.5	400.0	400.0	280.0	40.0	120.0	825.7	37.1	4.6
M6	Pumice	25	0	400.0	400.0	280.0	40.0	120.0	698.2	94.1	0.0
M7	Pumice	50	2	400.0	400.0	280.0	40.0	120.0	438.3	177.3	18.2
M8	Pumice	75	1	400.0	400.0	280.0	40.0	120.0	225.9	274.1	9.1
M9	Perlite	10	1	400.0	400.0	280.0	40.0	120.0	813.4	43.2	9.1
M10	Perlite	25	2	400.0	400.0	280.0	40.0	120.0	657.4	104.7	18.2
M11	Perlite	50	0	400.0	400.0	280.0	40.0	120.0	465.5	222.5	0.0
M12	Perlite	75	0.5	400.0	400.0	280.0	40.0	120.0	229.3	328.8	4.6
M13	Diatomite	10	2	400.0	400.0	280.0	40.0	120.0	788.9	38.7	18.2
M14	Diatomite	25	1	400.0	400.0	280.0	40.0	120.0	677.8	99.7	9.1
M15	Diatomite	50	0.5	400.0	400.0	280.0	40.0	120.0	458.7	202.4	4.6
M16	Diatomite	75	0	400.0	400.0	280.0	40.0	120.0	232.7	308.0	0.0
The	Without WTA	0	0	400.0	400.0	280.0	40.0	120.0	931.0	0.0	0.0

**Table 6:** ANOVA analysis according to Taguchi optimization.

Parameters	Source	DF	Adj SS	Adj MS	F-Value	*p*-Value	Effect (%)
28-day compressive strength	Coating material	3	108.812	36.271	18.3	0.002	76.2
	WTA content	3	29.822	9.941	5.02	0.045	20.9
	PPF volume	3	4.087	1.362	0.69	0.592	2.9
	Error	6	11.891	1.982	24.01		
	Total	15	154.612				
Flexural toughness	Coating material	3	58,589	19,530	0.62	0.627	31.2
	WTA content	3	38,229	12,743	0.41	0.755	20.6
	PPF volume	3	90,659	30,220	0.96	0.469	48.2
	Error	6	188,617	31,436			
	Total	15	376,094				
Oven dry density	Coating material	3	54,176	18,059	1.32	0.352	22.1
	WTA content	3	181,360	60,453	4.42	0.058	73.6
	PPF volume	3	10,557	3519	0.26	0.854	4.3
	Error	6	82,048	13,675			
	Total	15	328,141				
Apparent porosity	Coating material	3	34.034	11.3445	10.79	0.008	76.4
	WTA content	3	8.809	2.9362	2.79	0.132	19.8
	PPF volume	3	1.687	0.5623	0.53	0.675	3.8
	Error	6	6.308	1.0513			
	Total	15	50.837				
Capillary water absorption	Coating material	3	104.76	34.918	5.62	0.035	67.6
	WTA content	3	30.96	10.32	1.66	0.273	20.1
	PPF volume	3	19.13	6.375	1.03	0.445	12.3
	Error	6	37.27	6.211			
	Total	15	192.11				

**Table 7:** Verification experiment results.

Parameters		Optimum Mix-1 (Maximize)	Optimum Mix-2 (Minimize)
Compressive strength (MPa)	Predicted	15.5	
	Experimental	16.2	
	Difference (%)	−4.3	
Flexural toughness (N.mm)	Predicted	106.9	
	Experimental	114.3	
	Difference (%)	−6.5	
Apparent porosity (%)	Predicted		12.2
	Experimental		11.5
	Difference (%)		6.5
Capillary water absorption (kg/m^2^)	Predicted		7.8
	Experimental		7.6
	Difference (%)		2.6
Oven-dry density (kg/m^3^)	Predicted		1594
	Experimental		1561
	Difference (%)		2.1

## Data Availability

The original contributions presented in this study are included in the article; further inquiries can be directed to the corresponding author.
